# Ortholog-specific Spatial Expression of Vanins in the Respiratory Tract of Horses

**DOI:** 10.1369/00221554261474565

**Published:** 2026-08-31

**Authors:** Katharina Landmann, Florian Bartenschlager, David Meierhofer, Andreas Spree, Caroline Pisch, Annette Zeyner, Christiane L. Schnabel, Lars Mundhenk

**Affiliations:** Institute of Veterinary Pathology, Faculty of Veterinary Medicine, Freie Universität Berlin, Berlin, Germany; Institute of Veterinary Pathology, Faculty of Veterinary Medicine, Freie Universität Berlin, Berlin, Germany; Max Planck Institute for Molecular Genetics, Berlin, Germany; Institute of Veterinary Pathology, Faculty of Veterinary Medicine, Freie Universität Berlin, Berlin, Germany; Institute of Agricultural and Nutritional Sciences, Martin Luther University Halle-Wittenberg, Halle, Germany; Institute of Agricultural and Nutritional Sciences, Martin Luther University Halle-Wittenberg, Halle, Germany; Institute of Immunology, Faculty of Veterinary Medicine, Leipzig University, Leipzig, Germany; Institute of Veterinary Pathology, Faculty of Veterinary Medicine, Freie Universität Berlin, Berlin, Germany

**Keywords:** airways, asthma, horses, pantetheinase, Vanin

## Abstract

Equine asthma (EA) is the most prevalent chronic respiratory disease in horses, but molecules thought to be relevant in its pathogenesis are not yet fully understood. Recently, increased Vanin-1 (VNN1) levels were found in bronchoalveolar lavage fluid from horses with severe EA. Human VNN1 is known as a pantetheinase, which is associated with respiratory diseases such as asthma, but the equine vanins (eVNNs) are uncharacterized. We identified the domain architecture of eVNN in silico and analyzed its tissue expression in healthy horses using RT-PCR (*n*=5) and in situ hybridization (*n*=3). Recombinant eVNNs were expressed in HEK293 cells to assess cellular transport and glycosylation via Endo H and PNGase F treatment. Three putative functional *eVNNs* were identified on chromosome 10, each containing canonical Nitrilase superfamily domains. All genes showed a broad tissue expression, but distinct airway localization: *eVNN1* and *eVNN2* were predominantly expressed in respiratory epithelium, whereas *eVNN3* was localized in submucosal glands. eVNN1 and eVNN3 were secreted, while eVNN2 remained cell associated. Pantetheinase activity of recombinant eVNN1 was confirmed by mass spectrometry. The three eVNNs appear to cover different functional niches in equine airways, enabling future research into their putative role in EA.

## Introduction

Asthma is a ubiquitous, non-infectious disease characterized by chronic inflammation of the lower respiratory tract affecting both humans and horses worldwide.^1,2^ Up to 80% of horses are estimated to be affected by pathological respiratory findings associated with equine asthma (EA).^3,4^ As the condition shares key pathological features with human asthma and is considered a comparative model for the human disease,^5^ the nomenclature has been adapted: the formerly known inflammatory airway disease is now referred to as mild to moderate EA (MEA), and the previously known recurrent airway obstruction is now termed severe EA (SEA).^6^ Clinically, airway inflammation and excessive mucus production are common features of both phenotypes.^2,7^

Recently, the global proteomic composition of equine airway mucus was analyzed, revealing increased levels of Vanin-1 (VNN1) in bronchoalveolar lavage fluid from horses diagnosed with SEA.^8^ VNN1, which has been studied in humans and mice, is a member of the *Vanin* gene family, encoding a pantetheinase enzyme that hydrolyzes pantetheine into cysteamine and pantothenic acid (vitamin B5), a precursor for Coenzyme A.^9^ In humans and mice, this enzyme is widely expressed across tissues, and its role in diseases, including asthma, has often been associated with the regulation of signaling pathways leading to oxidative stress and inflammation.^10–13^ In murine asthma models, elevated *Vnn1* levels activated the PI3K/Akt/NF-κB pathway, leading to an increase in reactive oxygen species (ROS) and to airway inflammation.^10^ In addition, its enzymatic product, cysteamine, and its oxidized form, cystamine, have been suggested to increase ROS production in the liver through inhibition of glutathione peroxidase and γ-glutamylcysteine synthetase, respectively, which has not yet been proven in the lung.^14–17^ On the other hand, cysteamine has also proven to significantly attenuate airway hyperresponsiveness in mice, inhibiting asthma development, and exerted a positive therapeutic effect by decreasing inflammatory cells and chemokines in murine asthma models.^18^ Furthermore, human and murine VNN1 seem to be sensitive markers for corticosteroid responsiveness in asthma.^19^

Three human *VNN* genes have been identified; however, only human *VNN1* and *VNN2* encode functional proteins, while human *VNN3* represents a pseudogene.^20–22^ In contrast, the murine genome contains functional *Vnn1* and *Vnn3* genes but lacks an ortholog of *VNN2*.^20,21,23^ Thus, marked interspecies variations of this gene family are apparent. While the *VNN* family has been extensively studied in humans and mice, less is known about their molecular characteristics and functions in other species, including horses.

Our study aimed for molecular characterization and investigation of expression patterns of the equine *VNN* (*eVNN*) gene family. The domain organization of eVNN proteins was analyzed in silico. Moreover, equine orthologs of the *VNN* gene family were cloned to investigate their cellular transport and the enzymatic activity of *eVNN1*. Subsequently, their mRNA tissue and cellular expression patterns were identified in healthy horses. These findings contribute to a deeper understanding of the gene family’s characteristics and potential functions in equine airways.

## Materials and Methods

### Animals and Tissue Processing

Tissue samples were obtained from a total of 10 adult warmblood horses of both sexes, with ages ranging from 4 to 30 years and body weights ranging from 250 to 600 kg (Supplemental Table 1 of S1). Samples of healthy horses included in this study were either part of another research project with permission from the local authorities (State Office of Health and Social affairs, Saxony, Germany; approval number TVV63/21; H1, H2, H3, H4, H5, H6) or were sampled with written consent of the owners during routine pathological diagnostics conducted by the FU Berlin for other purposes than respiratory disease (State Office of Health and Social Affairs Berlin StN 010/24; H7, H8, H9, H10). Tissue samples of the respiratory tract (Supplemental Table 2 of S1) of five different horses included cranial (T1), middle (T2), and caudal (T3) trachea, large bronchi (LB), medium-sized bronchi (MB), small bronchi (SB), and cranial (L1) or caudal pulmonary parenchyma (L2). Cranial or caudal sections of the trachea were defined as the cranial or caudal third of the trachea, respectively. Lung samples (L1 and L2) were taken from the left lung lobes, while bronchial samples (LB, MB, and SB) originated from the right lobes. Samples of large bronchi were taken close to the bifurcation, whereas medium-sized and small bronchi were obtained from the third to fourth and fifth to sixth bronchial generations, respectively. In addition, extra-respiratory tissue samples (Supplemental Table 3 of S1) from four different horses were collected: skin, skeletal muscle, conjunctiva, liver, spleen, kidney, urinary bladder, adrenal gland, pancreas, glandular stomach, duodenum, jejunum, caecum, colon, rectum, aorta, right ventricle, right atrium, thyroid gland, cerebrum, cerebellum, and pituitary gland. All tissue samples were collected within 1–3 hr post-mortem to ensure sample integrity and were either snap-frozen after short immersion in 2-Methylbutane and stored at −80C or fixed in 4% neutral buffered formaldehyde for 24–48 hr before being embedded in paraffin.

All horses appeared clinically healthy according to a general clinical health assessment and underwent standard pathological examination. The horses were only included in this study if any gross or histopathological changes in the analyzed tissues were absent.

### In Silico Analyses of Equine, Human, and Murine VNN Genes and Proteins

Nucleotide mRNA sequences, as well as genomic location and exon-intron boundaries of all equine, human, and murine *VNN* gene isoforms, were retrieved from the GenBank DNA database (Assembly: TB-T2T GCF_041296265.1; Accession release: RS_2024_12)^24^ of the National Center for Biotechnology Information (NCBI) as well as the Ensembl database.^25^ The gene sequences were further compared with each other using the NCBI Basic Local Alignment Search Tool (BLAST)^26^ and Clustal Omega^27^ to verify homology. Putative protein domains and glycosylation sites were identified using SOSUI,^28^ PredGPI,^29^ NetNGlyc,^30^ and SignalP 5.0.^31^ Open reading frames (ORFs) of equine, human, and murine *VNN* genes were aligned using the Multiple Universal Sequence Comparison by Log-Expectation (MUSCLE) algorithm^32^ implemented in MEGA 11,^33^ and phylogenetic relationships were inferred using the maximum likelihood method implemented in MEGA 11.

### Molecular Cloning

Total RNA was extracted from respiratory tissue using the NucleoSpin RNA Kit (Macherey-Nagel, Düren, Germany), digested with DNase I (Thermo Fisher Scientific, Waltham, MA), and reverse transcribed with oligo(dT) primers using the RevertAid H Minus First Strand cDNA Synthesis Kit (Thermo Fisher Scientific) following the manufacturer’s protocol. Gene-specific primers (Supplemental Table 4 of S2) were designed for the amplification of the ORF of *eVNN1* (based on the reference sequence with the NCBI accession number XM_023651108.2), *eVNN2* (XM_070223887.1), and *eVNN3* (XM_001504346.5). The primer sequences included restriction sites for KpnI or XbaI and lacked the stop codon. The ORF of each gene was amplified with the Q5-Hot Start High-Fidelity 2X Master Mix (New England Biolabs, Ipswich, Massachusetts) according to the manufacturer’s protocol. Polymerase chain reaction (PCR) products were purified using the PCR Clean-Up and Gel Extraction Kit (Macherey-Nagel). The ORFs of *eVNN* orthologs were then inserted into vectors bearing a carboxyl (C)-terminal hexahistidine–Twin-Strep tag. *eVNN1* was cloned into a custom-designed, modified pcDNA3.1 vector (GeneArt, Thermo Fisher Scientific; S3), and *eVNN2* or *eVNN3* was cloned into a custom-designed, modified pcDNA3.4 vector (GeneArt, Thermo Fisher Scientific; S3). Three positive cloned plasmids of each gene were analyzed by Sanger sequencing and compared with the corresponding NCBI GenBank reference sequences. In addition, PCR products of the amplified ORF of each *Vanin* gene from an additional horse were sequenced to verify sequence accuracy. For recombinant protein expression and purification, eVNN1 and equine Chloride channel accessory 1 (eCLCA1), the latter previously cloned in another study,^34^ were subcloned into pDSG102 expression vectors (IBA Lifesciences GmbH, Göttingen, Germany) using the direct transfer cloning method according to the manufacturer’s instructions. These constructs encoded recombinant eVNN1 with a C-terminal hexahistidine-Twin-Strep tag and recombinant eCLCA1 with a C-terminal Twin-Strep tag (S3).

### Transient Transfection of Adherent HEK293 Cells for Cellular Transport Analyses

HEK293 cells were cultured in six-well plates in Dulbecco’s modified Eagle’s medium (DMEM; Thermo Fisher Scientific) supplemented with 10% heat-inactivated fetal calf serum (Bio&Sell, Feucht, Germany), 1% *N*-2-hydroxyethylpiperazine-*N*′-2-ethane sulphonic acid (HEPES; Biowest, Nuaillé, France), and 1% penicillin/streptomycin (Biowest) at 37C and 5% CO_2_ until they reached 80–90% confluency. Cells were transfected with the *eVNN1*, *eVNN2*, or *eVNN3* encoding plasmids using the Turbofect transfection reagent kit (Thermo Fisher Scientific) according to the manufacturer’s protocol. Vectors lacking the e*VNN* inserts were used as mock controls. Forty-eight hours after transfection, the supernatant was removed, and the cells were washed with prewarmed phosphate-buffered saline (PBS; Biowest) before adding 500 µl of fresh DMEM for 6 hr. For extracellular protein analysis, the supernatant was centrifuged at 11,000 *×* g for 2 min to remove cells and subsequently precipitated with ethanol (Carl Roth). After centrifugation at 14,000 *×* g for 30 min at 4C, the resulting pellet was resuspended in lysis buffer (25 mM Tris-HCl pH 8.0, Thermo Fisher Scientific; 50 mM NaCl, Carl Roth, Karlsruhe, Germany; 0.5% Triton-X-100, Carl Roth; 0.5% Na-Deoxycholate, Carl Roth; 1 tablet/50 ml cOmplete Protease Inhibitor Cocktail, Roche, Basel, Switzerland) and incubated on ice for 30 min. Cellular protein was extracted by incubating the cells with lysis buffer for 30 min on ice and centrifugation at 14,000 *×* g for 15 min at 4C to remove cell debris. The resulting protein extract was used to determine protein concentration with the Micro Bicinchoninic Acid (BCA) Protein Assay Kit (Thermo Fisher Scientific) according to the manufacturer’s protocol.

### Endoglycosidase Treatment

The glycosylation pattern of proteins in both supernatant and cell lysate was analyzed by deglycosylation using Peptide-*N*-Glycosidase (PNGase F, New England Biolabs) to remove all asparagine (N)-linked oligosaccharides from glycoproteins, or Endoglycosidase H (Endo H, New England Biolabs) to specifically cleave only high-mannose N-linked glycans, respectively. The samples were incubated with the respective enzymes according to the manufacturer’s protocol or left untreated. Subsequently, the samples were analyzed via immunoblotting.

### Immunoblotting

Untreated and deglycosylated cell lysates as well as supernatants were analyzed via immunoblotting (4 µg/sample). Samples were mixed with Laemmli buffer (6% SDS, 30% glycerin, 150 mM DTT, 150 mM Tris-HCl, pH 6.8 and 0.02% bromophenol blue; all obtained from Carl Roth) in a 1:5 ratio and boiled for 10 min before being separated by a 10% SDS-PAGE acrylamide gel (Mini-PROTEAN TGX; Bio-Rad Laboratories Inc., Hercules, California) in Tris-glycine buffer (Carl Roth). Proteins were then transferred onto a polyvinylidene difluoride membrane (0.45 µm pore size, Biozym Scientific GmbH, Hessisch Oldendorf, Germany) and blocked with 5% non-fat milk in Tris-buffered saline with Tween-20 (TBS-T; 137 mM NaCl; 2.7 mM KCl, Carl Roth; 24.8 mM Tris base, Carl Roth; 0.1% Tween-20, AppliChem GmbH, Darmstadt, Germany) for 1 hr at room temperature (RT). The membranes were subsequently incubated overnight on a 3D shaker at 4C with a primary Anti-Strep antibody (Strep Tag II Monoclonal Antibody, CSB-MA000316, Cusabio Biotechnology, Houston, TX) diluted 1:1000. After washing three times with TBS-T for 15 min each, the membranes were incubated with a horseradish peroxidase–conjugated secondary goat anti-mouse antibody (115-035-003, Jackson ImmunoResearch Laboratories, Inc., West Grove, PA) at a 1:10,000 dilution for 1 hr at RT on a 3D shaker. Following three additional washes with TBS-T, the membranes were incubated with SuperSignal West Pico chemiluminescent substrate (Thermo Fisher Scientific), and protein bands were visualized using the Azure 600 Western Blot Imaging System (Biozym Scientific GmbH).

### Expression and Affinity Purification of Recombinant Proteins for Enzymatic Activity Analysis

Recombinant eVNN1 was produced to evaluate its enzymatic activity. In parallel, recombinant equine Chloride channel accessory 1 (eCLCA1), a matrix metalloproteinase without pantetheinase activity, was generated as a negative control for the enzymatic assay. eVNN1 and eCLCA1 were expressed in MEXi-293E cells (IBA Lifesciences) according to the manufacturer’s recommendations, carrying a C-terminal hexahistidine-Twin-Strep-tag or a C-terminal Twin-Strep-tag, respectively. Briefly, MEXi-293E cells were transiently transfected with the *eVNN1* or *eCLCA1* containing pDSG102 plasmids using polyethyleneimine (25 kDa, linear, 1 mg/ml, pH 7.0, Polysciences, Warrington, PA) in MEXi-TM medium (IBA Lifesciences). After 4 hr, MEXi-CM medium was added, and the cell culture supernatant was collected after 8 days of incubation at 37C and 5% CO_2_ on a 19-mm orbital shaker (Thermo Fisher Scientific) at 125 rpm. Cells were removed from the supernatant by centrifugation at 300 *×* g for 10 min at 4C. To block endogenous biotin, the received supernatant was supplemented with 10% (v/v) 10× Buffer W (IBA Lifesciences) and incubated for 20 min with BioLock biotin blocking solution (IBA Lifesciences) at RT and centrifuged at 10,000 *×* g for 20 min at 4C. After confirming that the pH of the modified supernatant was between 7.0 and 8.0, it was filtered through a 0.2 µm PTFE filter (Carl Roth) and degassed.

The recombinant proteins were subsequently purified by affinity chromatography using Strep-TactinXT 4Flow FPLC columns (IBA Lifesciences) on an ÄKTA start chromatography system (Cytiva, Marlborough, MA) according to the manufacturer’s protocol. The column was equilibrated with 10 column volumes (CV) of 1× Buffer W. The cartridge was loaded with the supernatant at 1 ml/min and washed with 10 CV 1× Buffer W. The recombinant proteins were eluted with 10 CV 1× Buffer BXT (IBA Lifesciences) and concentrated using Vivaspin 15 R 10 kDa cut-off columns (Sartorius, Göttingen, Germany). Protein concentrations were determined fluorometrically using the Qubit Protein Assay Kit (Thermo Fisher Scientific) and a DeNovix QFX fluorometer (DeNovix, Wilmington, DE).

### Analysis of Pantetheinase Activity of eVNN1

Purity and identity of both recombinant proteins were assessed by immunoblotting of the purified proteins as described above, using Strep Tag II monoclonal primary antibodies (CSB-MA000316, Cusabio, diluted 1:1000) and total protein staining using the Amersham quickstain kit (Cytiva). Both proteins were subsequently dialyzed against Dulbecco’s PBS (Biowest) using Slide-A-Lyzer MINI dialysis devices with 10 kDa cutoff (Thermo Fisher Scientific). The concentrations of both proteins were determined fluorometrically as described and adjusted to the desired concentrations of 1 mg/ml. Enzymatic assays were performed by incubating eVNN1 (10 nM) with pantetheine (Sigma-Aldrich, St. Louis, MO) in various concentrations (0 µM, 200 µM, 500 µM, and 1 mM). Reaction conditions were adapted from Ruan et al.^35^ In short, each reaction was carried out in potassium phosphate buffer (100 mM, pH 8.0; Biowest) containing dithiothreitol (DTT; 5 mM; AppliChem), bovine serum albumin (BSA; 0.01%; Carl Roth), and Brij-35 (0.0025%; Carl Roth) for 1 hr at 37C. Controls either lacked recombinant eVNN1 or consisted of equally concentrated eCLCA1 instead of eVNN1. Positive controls contained commercially obtained pantetheine, pantothenic acid, and cysteamine (100 µM; all obtained from Sigma-Aldrich) in the same buffer.

### Liquid Chromatography With Tandem Mass Spectrometry

The potential pantetheinase activity of eVNN1 was evaluated using a targeted mass spectrometry approach, which directly measures the quantities of the substrate, pantetheine, and its product, pantothenic acid. First, a multiple reaction monitoring-based method was created by tuning pure compounds of cysteamine, pantetheine, and pantothenic acid, identifying the three most abundant transitions per compound and their optimized instrument settings, their ion ratios, and the retention time on a reverse-phase column.

Samples of the enzymatic assays, including the positive and negative controls, were prepared for liquid chromatography with tandem mass spectrometry (LC-MS/MS) analysis as follows: 200 µl of each sample was used, and protein precipitation was achieved by adding four volumes of ice-cold (−20C) acetone. The samples were then vortexed briefly and stored overnight at −20C before being centrifuged at 20,000 *×* g for 10 min at 4C. The supernatants were concentrated in a vacuum concentrator (RC1010; Thermo Fisher Scientific) until dryness and reconstituted in 50 µl of MS-grade water containing 0.1% formic acid and the internal standard valine-D8 to a final concentration of 20 pmol. The samples were transferred to microvolume inserts, and 12 µl was injected for chromatographic separation on a Reprosil-PUR C18-AQ column (1.9 µm, 120 Å, 150 × 2 mm ID; Dr. Maisch, Ammerbuch, Germany) in technical duplicates. A 1290 series UHPLC (Agilent, Santa Clara, CA) coupled online to a QTrap 6500 triple quadrupole hybrid ion trap mass spectrometer (Sciex, Foster City, CA) was used as an LC-MS instrument, applying electro spray ionization in positive mode. Metabolites were separated using a linear gradient over 10 min. The mobile phase consisted of 0.1% formic acid in water (A) and 0.1% formic acid in acetonitrile (B). Metabolite intensities were quantified using MultiQuant Software v.2.1.1 (Sciex), with manual verification of all peaks. Measurements were normalized using the internal standard.

Area under the curve (AUC) values were baseline-corrected and analyzed using R Studio (version 4.4.1). Experimental groups were defined as “eVNN1,” “eCLCA1,” and “without enzyme,” depending on the enzyme added to the reaction. Pantetheine concentrations were treated as categorical variables (0, 200, 500, and 1000 µM). AUC values were log_10_-transformed before statistical testing. For each pantetheine concentration, pairwise comparison between groups (“eVNN1” vs “without enzyme” and “eVNN1” vs “eCLCA1”) was performed using Welch’s two-sample *t*-test. Data visualization was performed, displaying mean log_10_(AUC) values, ± standard error, and significance in grouped bar plots.

### Analysis of mRNA Tissue Expression Via RT-qPCR

Total RNA was isolated from all respiratory and extra-respiratory tissue samples (S1) using the NucleoSpin RNA Mini Isolation Kit (Macherey-Nagel) according to the manufacturer’s instructions. RNA purity and concentration were determined using Nanodrop (Thermo Fisher Scientific). Five hundred nanograms of isolated RNA was reverse transcribed using the iScript cDNA Synthesis Kit (Bio-Rad) as recommended by the manufacturer. Specific exon–exon boundary spanning primers were designed for each *eVNN* using the NCBI Primer-BLAST tool.^36^ Each primer (Supplemental Table 4 of S2) consisted of 20–21 nucleotides, with a guanine-cytosine content of less than 55%, and was designed to produce a single product consisting of up to 200 bp. PCR efficiency was assessed as previously described by Bartenschlager et al.^37^ Sequencing of the PCR product confirmed amplification of the target gene. SYBR green qPCR assay (Bio-Rad) was run, including inter-run calibrators to normalize the data between plates, as well as no-template controls (NTC) and the reference gene *Elongation Factor 1α* (EF1α).^38^ The Maestro CFX Manager software (Bio-Rad) was used for data acquisition. Technical duplicates with a standard deviation <0.5 quantification cycles (Cq) were performed for each sample. Cq values of each sample were visualized using R Studio.

### Analysis of Cellular Expression Pattern Via In Situ Hybridization

The cellular expression pattern of all *eVNN* genes was analyzed using the RNAScope 2.5 HD Assay—Red Kit (Advanced Cell Diagnostics, Newark, CA) on 4 µm formalin-fixed, paraffin-embedded (FFPE) sections of the respiratory tract, specifically T2, LB, MB, and SB. Specific probes were designed for each *eVNN* ortholog, based on the reference sequences: *eVNN1*, targeting mRNA section 151–1670; *eVNN2*, targeting mRNA section 279–1748; and *eVNN3*, targeting mRNA section 283–1800. In addition, a probe targeting equine Ubiquitin C (EcUBC; Cat No. 512131; Advanced Cell Diagnostics), a widely expressed housekeeper gene, served as a technical positive control as well as RNA integrity control. A probe targeting bacterial dihydrodipicolinate reductase (DapB; Cat No. 320871; Advanced Cell Diagnostics) was used as negative control. Deparaffinization of FFPE-tissue, target retrieval, and hybridization were performed as recommended by the manufacturer, with the exception of an extended incubation time of 50 min for amplification reagent 5 (AMP 5) for all evaluated tissues. Mayer’s hematoxylin (Bio-Rad) was used as counter stain. All slides were scanned with the Aperio AT2 scanner (Leica Biosystems, Nussloch, Germany) within 2 weeks.

### Quantification of In Situ Hybridization Signal

RNAscope signal quantification was conducted using QuPath version 0.5.0.^39^ Scanned whole-slide images were imported into the software. Color deconvolution was applied uniformly across all images, using consistent values for “Hematoxylin,” “ISH-Signal,” and “Background.” Whole tissue annotations were created manually for regions of respiratory epithelium and submucosal glands and classified according to the tissue type. Epithelial annotations of trachea, bifurcation, medium-sized bronchi, small bronchi, and bronchioles were defined as the area from the basement to the apical membrane. Signal detection in the alveoli was assessed by randomly placing 10 equally sized, squared regions (631,829.5 µm^2^) within pulmonary parenchyma (PP) without interstitial tissue, blood vessels, or excessive air spaces. Cell detection was performed using different parameter settings for glandular and epithelial cells or parenchymal cells (Groovy script including all parameters in S4). A pixel classifier was trained on 15 representative images from the same dataset to detect RNAscope signals. This classifier was then applied to each annotation and the corresponding individual cells, allowing quantification of both the percentage of positive pixels within each whole tissue annotation and the proportion of positive cells per annotation. To account for nonspecific hybridization and technical background, RNAscope signal values were baseline-corrected by subtracting the mean percentage of positive pixels measured in DapB-negative controls for each anatomical annotation and each horse. Percentages of positive pixels were analyzed using R Studio. For each anatomical annotation (respiratory epithelium and submucosal glands for T2, LB, and MB, respiratory epithelium for SB and bronchioles, and alveoli for pulmonary parenchyma), the median and interquartile range of positive pixels were calculated. Data were log(x+1) transformed to reduce skewness and improve visualization and plotted as bar plots for each gene, displaying the distribution of RNAscope signals across anatomical regions.

### Utilization of Large Language Models

Large language models were utilized to support both image analysis scripting and manuscript preparation. Specifically, ChatGPT (GPT-4o, Open AI) was used to assist in the generation and refinement of Groovy scripts for QuPath (S4), enabling the development of an automated and standardized image analysis workflow that was uniformly applied to all images. All generated scripts (S4) were carefully reviewed, tested, and validated before use to ensure analytical accuracy and reproducibility. In addition, ChatGPT was employed to improve the clarity of the manuscript text and to correct grammatical and spelling errors. Scientific interpretations, data analysis decisions, or result generation were not performed by the language model.

## Results

### The Equine Genome Contains Three VNN Genes Located on Chromosome 10

**Figure 1.**
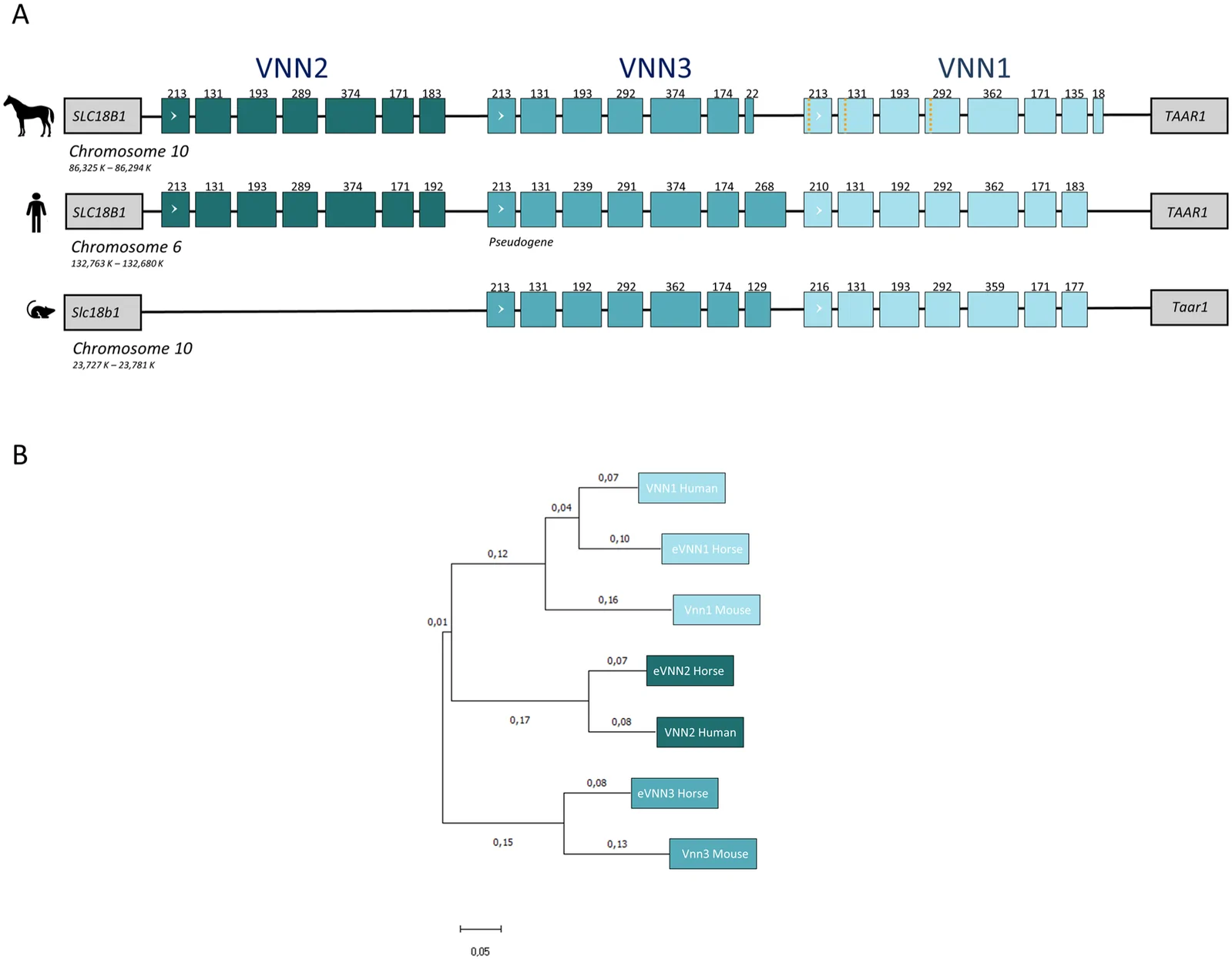
Three putative functional *VNN* members exist in horses. (A) Graphical alignment of *VNN* orthologs and homologs. Three functional *VNN* genes were identified in horses, in contrast to humans and mice. In all species analyzed, the *VNN* genes share the same transcriptional orientation and are flanked by the *SLC18B1* and *TAAR1* genes. Exons are depicted as boxes. Numbers above each box represent the nucleotide length of the corresponding exon. Yellow dotted lines indicate single-nucleotide polymorphisms (SNPs) of sequenced eVNN1 ORFs. Light teal represents *VNN1*, dark teal *VNN2*, and medium teal *VNN2.* (B) Maximum likelihood tree based on MUSCLE alignment of equine, human, and murine *VNN* coding sequences. The *VNN* orthologs of each species cluster together. Evolutionary distances are indicated by numbers.

In silico analyses revealed three e*VNN* genes in horses, which is similar to humans but in contrast to mice. *eVNN1*, *eVNN2*, and *eVNN3* clustered on chromosome 10 in the same transcriptional orientation (Fig. 1A). As in humans and mice, the *eVNNs* are flanked by the *Solute Carrier Family 18 Member B1 (SLC18B1)* and *Trace Amine Associated Receptor 1* (TAAR1) genes (Fig. 1A). In contrast to humans, all three e*VNN* genes encode putative functional proteins. All *VNN* family members in horses, humans, and mice typically consist of seven exons, with an exception of *eVNN1*, which includes an additional eighth exon. In total, five *eVNN1* transcript variants, with differences in protein coding sequences, were listed in the database (XM_023651105.2, XM_070223882.1, XM_023651108.2, XM_070223884.1, and XM_023651110.2), ranging from 1473 to 1545 bp in length. These variants differ only in the length of the 3′ end nucleotides of the seventh or eighth exon. For *eVNN2*, only one transcript of 1554 bp was identified (XM_070223887.1). For *eVNN3*, two transcript variants with 1299 bp and 1536 bp were identified (XM_001504346.5 and XM_005596934.4, respectively), with the first variant exhibiting shorter sixth and seventh exons compared with the second. Comparison of the sequenced *eVNN1* ORFs from two horses to the reference sequence of the NCBI GenBank identified three single-nucleotide polymorphisms (SNPs; S3): two synonymous variants at c.37C>T and at c.228C>T and a non-synonymous variant at position c.655A>G, resulting in a substitution of p.(Asn219Asp). No SNPs were identified when comparing the sequences of the amplified *eVNN2* and *eVNN3* ORFS with the respective reference sequences. The maximum likelihood phylogenetic analysis revealed three clades corresponding to the paralogous *VNN1*, *VNN2*, and *VNN3* genes. Within each clade, sequences grouped according to orthology, with human and horse sequences clustering more closely together than with mouse sequences (Fig. 1B).

### eVNNs Share Canonical Domains of the Nitrilase Superfamily

**Figure 2.**
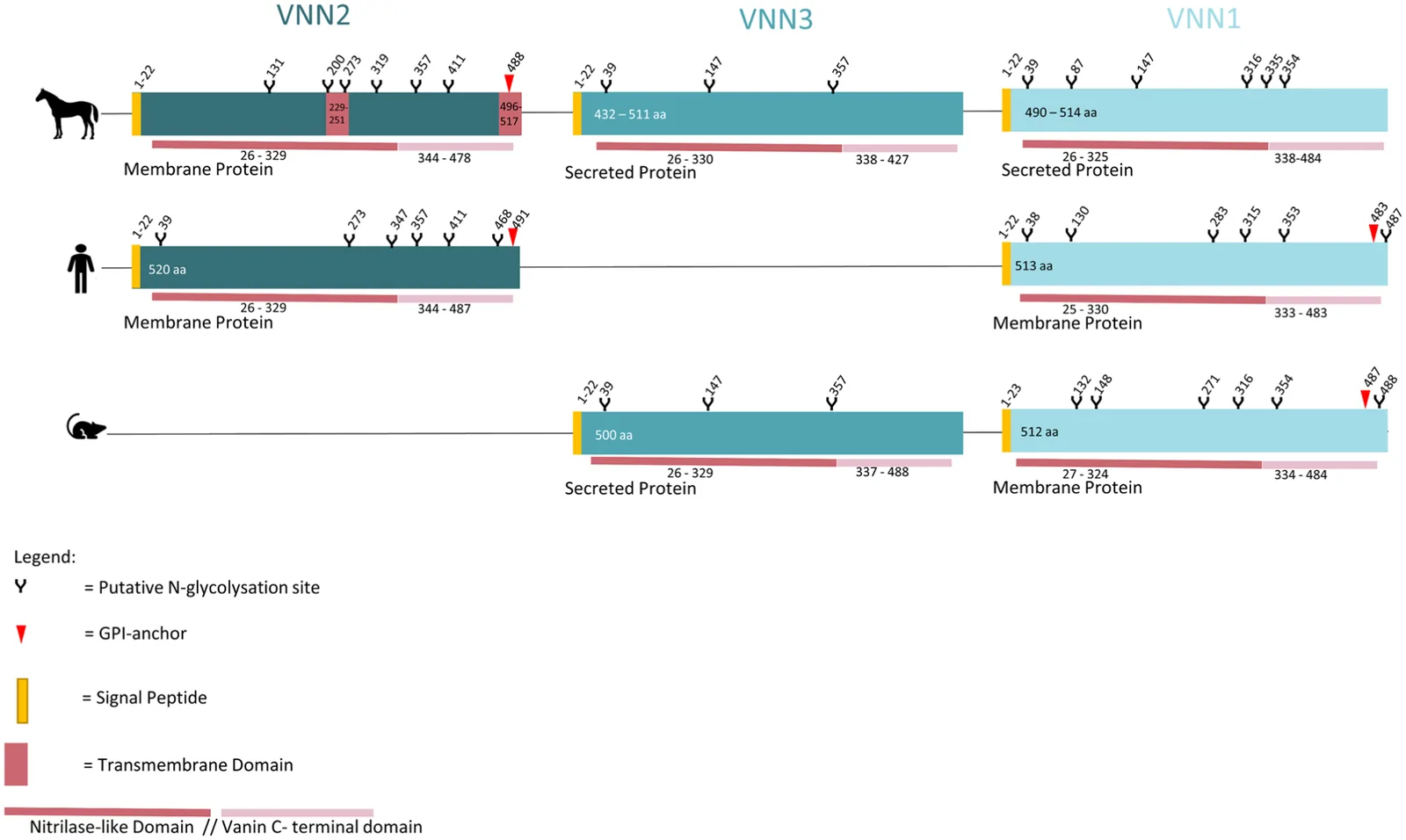
In silico protein structure of *VNNs.* Domain organization, predicted site of activity, and putative post-translational modifications of equine, human, and murine VNN1, VNN2, and VNN3 proteins. Putative N-glycosylation sites (γ) and predicted GPI-anchor sites (red arrowheads) are indicated. Protein length in amino acids (aa) is shown within the scheme. A range of aa are provided to encompass all NCBI-listed protein isoforms of each gene. Predicted site of activity (secreted vs membrane-associated) is based on the presence or absence of a predicted GPI-anchor or putative transmembrane domain, as indicated below each protein. Interspecies differences can be seen in the site of activity of VNN1, with eVNN1 being a secreted protein and its human and murine orthologs being GPI-anchored membrane proteins.

All eVNN proteins, like their human and murine orthologs, contain an amino (N)-terminal nitrilase-related domain that is commonly found in other members of the nitrilase superfamily (Fig. 2). In addition, a C-terminal Vanin domain, characteristic of each VNN paralog, was identified in all eVNN proteins (Fig. 2).

### eVNN1 and eVNN3 Were Secreted Whereas eVNN2 Remained Cell-Associated

In silico analyses revealed that all VNNs, including the equine members, were predicted to share a 22–23 amino acid (aa) signal peptide at the N-terminus, for entry into the endoplasmic reticulum (Fig. 2). In contrast to its human and murine homologs, no GPI-anchor or transmembrane domain (TMD) was predicted for eVNN1, which suggests it as a fully secreted and soluble protein. For eVNN2, a hydrophobic segment spanning residues 229–251 was predicted as a TMD. A second highly hydrophobic region, spanning residues 496–517 in the C-terminal part of eVNN2, was also predicted as a putative TMD. However, in silico analyses indicated that this C-terminal hydrophobic segment contains a GPI-anchor attachment site. A similar feature was observed in human VNN2. It contains consensus sequence characteristics typical of GPI anchoring, including a cleavage site followed by a spacer and a hydrophobic stretch, suggesting that this region more likely functions as a GPI-anchor signal rather than a TMD per se.^23,40^ Identical to Vnn3 and eVNN1, eVNN3 is predicted to be a soluble, fully secreted protein (Fig. 2). Potential N-linked glycosylation sites were found in all aa sequences of the eVNNs: eVNN1 is predicted to possess six, eVNN2 seven, and eVNN3 three glycosylation sites (Fig. 2).

**Figure 3.**
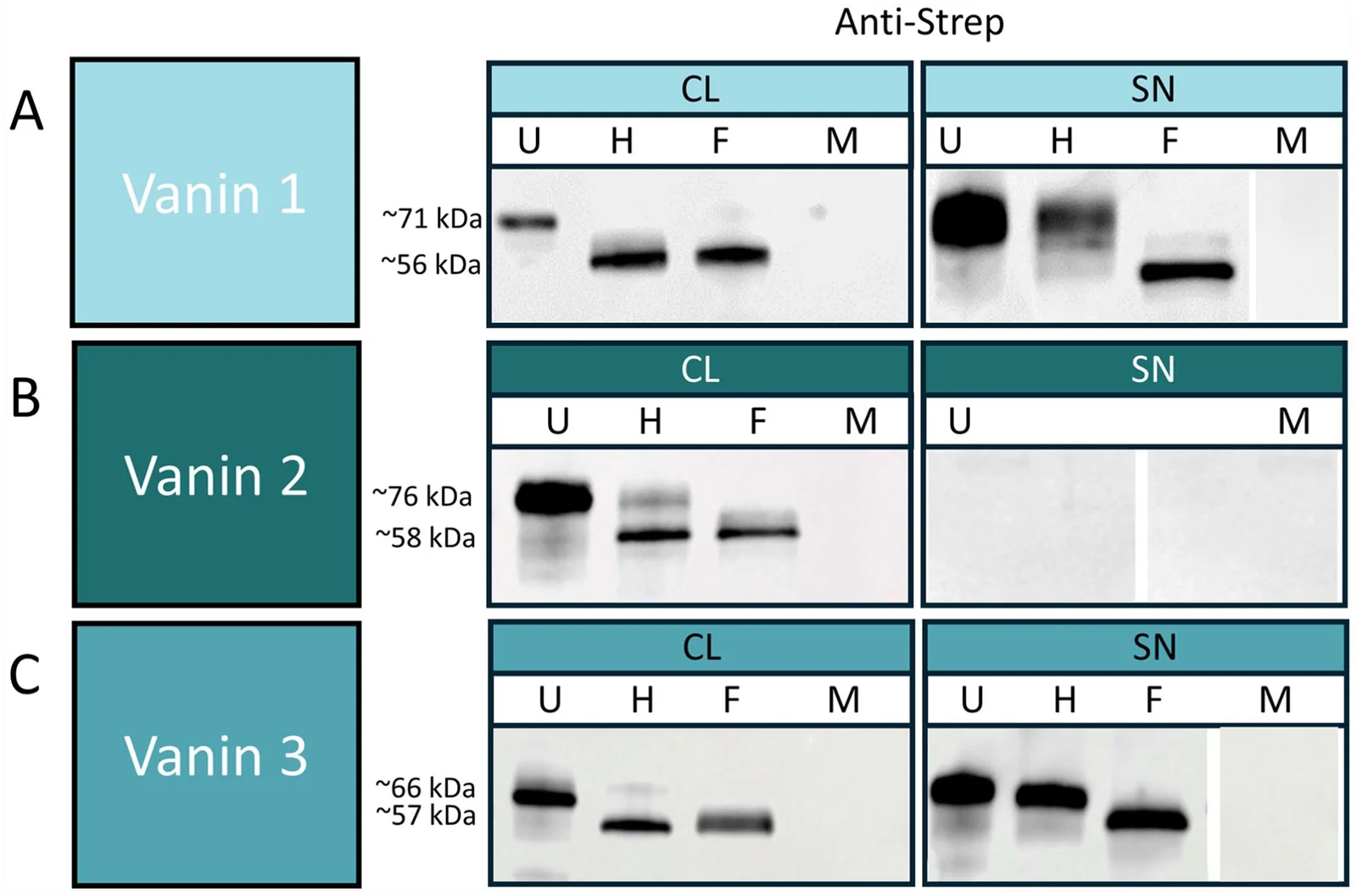
Posttranslational modification and cellular transport of the equine Vanin proteins. (A) eVNN1, (B) eVNN2, and (C) eVNN3. Immunoblot analyses of cell lysates (CL) and supernatants (SN) from HEK293 cells transfected with eVNN1, eVNN2, and eVNN3 or mock plasmids (M). Four micrograms of protein lysates were left untreated (U) or treated with endoglycosidases Endo H (H), PNGase F (F). Complex glycosylation, indicative of a mature protein, can be seen for eVNN1 (A) and eVNN3 (C) in the SN, and for eVNN2 (B) in the CL. Original immunoblots are presented in the Supplementals (S8).

To validate these predictions for the eVNNs experimentally, the cellular transport, processing, and glycosylation pattern of the recombinant proteins were analyzed in cell lysates and supernatants using immunoblotting as a read-out. Deglycosylation with Endo H removes high mannose and some N-linked glycans, indicating ER or early Golgi glycosylation, whereas PNGase F cleaves nearly all N-linked glycans, suggesting complex glycosylation in the medial Golgi. For eVNN1, a ~71 kDa protein band was detected in the lysate of transfected cells (Fig. 3A). Both Endo H- and PNGase F-treated samples resulted in a shift to ~56 kDa, indicating an immature, intracellular, high-mannose glycosylation pattern. A slightly larger band was identified in the corresponding supernatant. The supernatant-derived protein was resistant to Endo H but showed a downward shift in molecular weight to ~56 kDa after treatment with PNGase F, indicating further processing to complex glycosylation in the medial Golgi (Fig. 3A).

For eVNN2, a ~76 kDa band was detected in cell lysates without further processing into the supernatant, supporting its cell association as predicted in silico. Enzymatic digestion revealed PNGase F sensitivity as well as a mix of Endo H-resistant (~76 kDa) and Endo H-sensitive bands (~58 kDa) in the cell lysate, suggesting the presence of both mature and immature forms in the cell. eVNN2 was not detected in the culture supernatant, indicating that eVNN2 is not released as a soluble, secreted protein under these in vitro conditions (Fig. 3B).

eVNN3 showed a similar cellular transportation pattern as eVNN1. In the cell lysate, a ~66 kDa band corresponding to the glycosylated form was observed. After Endo H digestion, a predominantly Endo H-sensitive band appeared at ~57 kDa, along with a minor Endo H-resistant fraction remaining at ~66 kDa. PNGase F digestion also caused a downward shift to ~57 kDa, suggesting the protein is largely modified with high-mannose N-glycans. In the supernatant, a slightly larger glycosylated band was detected. Following PNGase F digestion, the supernatant-derived protein exhibited a downward shift to ~57 kDa but remained resistant to Endo H, suggesting a complex, mature glycosylation pattern in the supernatant (Fig. 3C).

In summary, these findings support the in silico predictions: eVNN1 and eVNN3 are secreted glycoproteins, whereas the mature eVNN2 protein remains cell-associated.

### Distinct Extra-Respiratory Tissue Distribution Pattern of eVNN Paralogs

**Figure 4.**
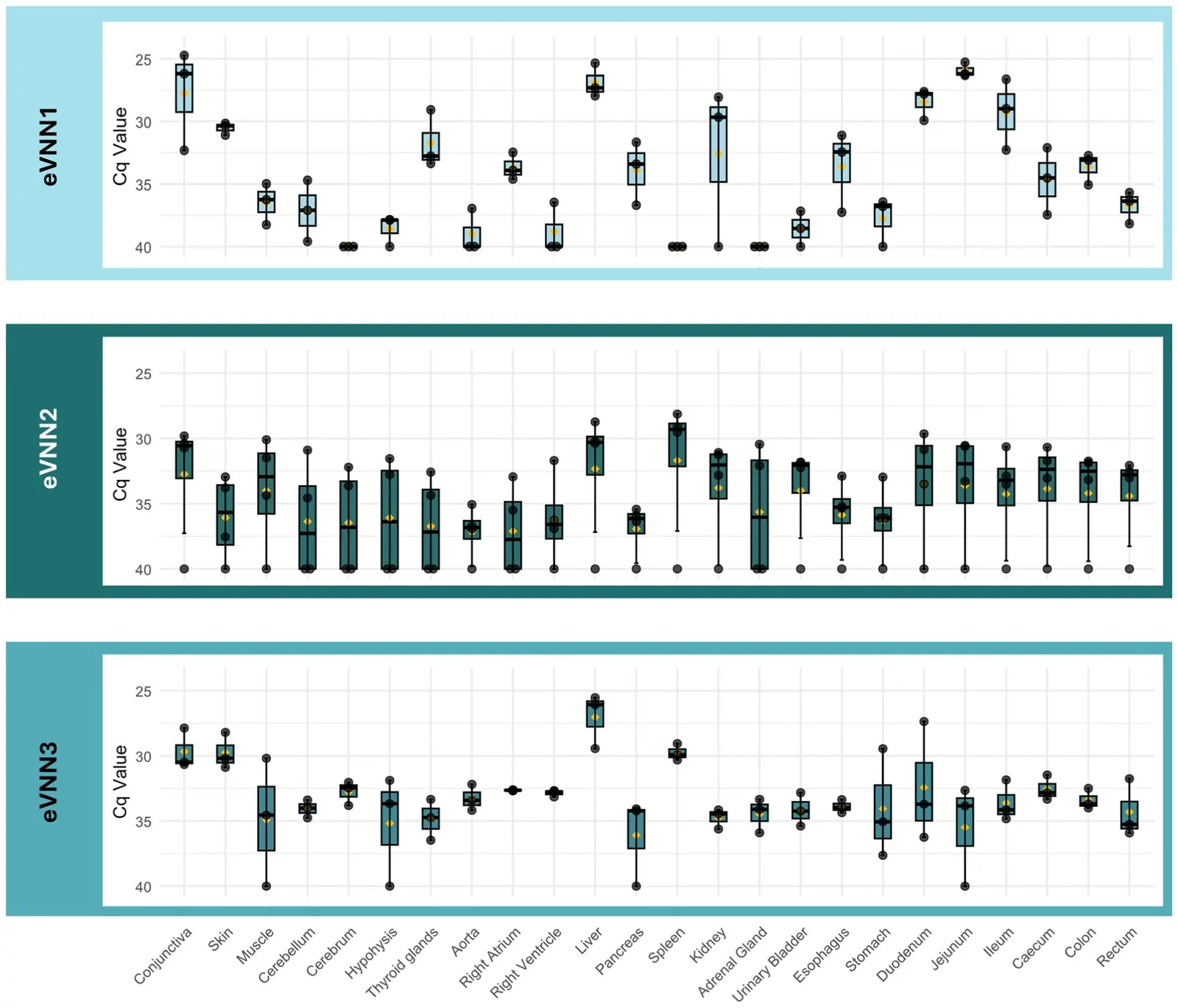
Equine Vanin mRNA paralogues exhibit broad organ distribution. mRNA expression across various tissues of *eVNN1*, *eVNN2*, and *eVNN3* detected by quantitative RT-PCR. Dots represent the mean Cq value of technical duplicates of each individual horse (*eVNN1* and *eVNN3: n*=3; *eVNN2: n*=4). Boxes represent the interquartile range (IQR, Q1–Q3), with the median indicated by the horizontal line inside each box. Whiskers (error bars) extend to the most extreme non-outlier values, defined as Q1 − 1.5 × IQR and Q3 + 1.5 × IQR. The yellow diamond indicates the mean Cq value for each location. The y-axis is reversed, so lower Cq values appear higher on the plot, reflecting higher relative transcript abundance.

*eVNNs* were detected in all investigated tissues with various mRNA expression levels, as reflected by differing Cq values (Fig. 4). Of all organs, the liver showed the lowest Cq values for all three *eVNN* paralogs (mean Cq values: *eVNN1 =* 26.8, *eVNN2* = 29.8, *eVNN3* = 27), indicating comparatively high *eVNN* levels. The lowest Cq value for each individual gene was observed in the jejunum for *eVNN1* (mean Cq = 25.9), the spleen for *eVNN2* (mean Cq = 28.9), and the liver for *eVNN3* (mean Cq = 27). *eVNN1* mRNA was not detected in the cerebrum, spleen, and adrenal gland. Notably, individual horses showed only low levels or a complete absence of *eVNN1*, *eVNN2*, or *eVNN3* in specific organs. Furthermore, one animal (H9) did not show any detectable *eVNN2* mRNA across all examined extra-respiratory tissues, highlighting considerable inter-individual variability in *eVNN2* expression (Fig. 4).

### All eVNNs Are Expressed in the Respiratory Tract of Horses With Different Cellular Expression Patterns

**Figure 5.**
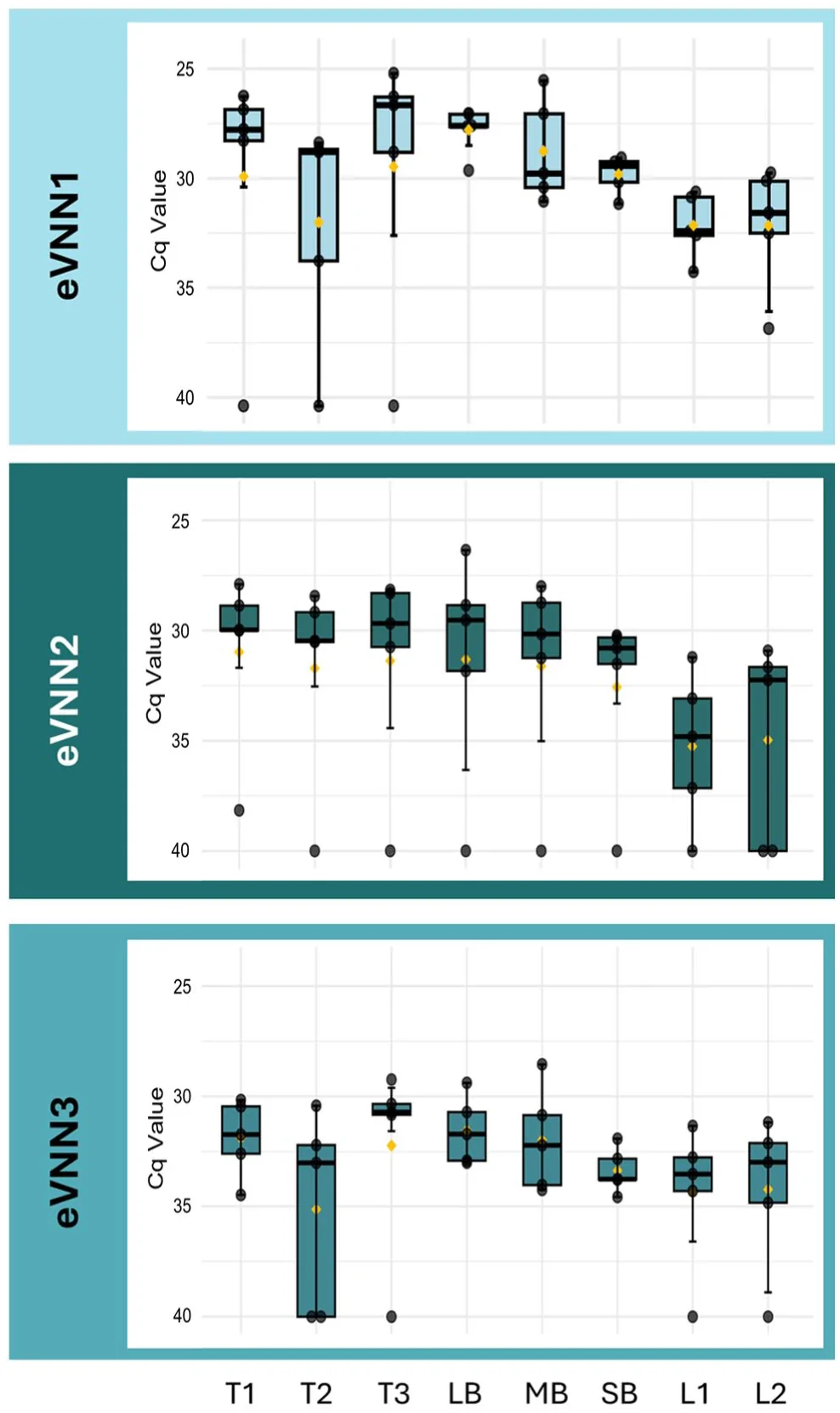
*eVNN* mRNA paralogs within respiratory tissue. mRNA expression of *eVNN1*, *eVNN2*, and *eVNN3* within respiratory tissue detected by quantitative RT-PCR. Tissue samples of the respiratory tract included cranial (T1), middle (T2), and caudal (T3) trachea, large bronchi (LB), medium-sized bronchi (MB), small bronchi (SB), and cranial- (L1) and caudal-pulmonary parenchyma (L2). Dots represent the mean Cq value of technical duplicates of each individual horse (*n*=5). Boxes represent the interquartile range (IQR, Q1–Q3), with the median indicated by the horizontal line inside each box. Whiskers (error bars) extend to the most extreme non-outlier values, defined as Q1 – 1.5 × IQR and Q3 + 1.5 × IQR. The yellow diamond indicates the mean Cq value for each location. The y-axis is reversed, so lower Cq values appear higher on the plot, reflecting higher relative transcript abundance.

All *eVNN* paralogs were expressed in all investigated sections of the respiratory tract (Fig. 5). The variations in eVNN expression levels between different respiratory tissue locations were smaller than those between extra-respiratory tissues. Notably, the Cq values for all *eVNNs* were generally lower in the bronchial mucosa (LB-SB) compared with pulmonary parenchyma (L1–L2). For *eVNN1*, the lowest mean Cq value was detected in LB (mean Cq = 27.4), while the highest Cq values were found in L2 (mean Cq = 31.8) and L1 (mean Cq = 31.8). The lowest mean Cq values of *eVNN2* were identified in T1 (mean Cq = 31.0), with the highest values again in L2 (mean Cq = 35.3) and L1 (mean Cq = 34.9). *eVNN3* exhibited the lowest overall mRNA expression among the *eVNN* family in the respiratory tract. Its lowest Cq values were detected in the LB (mean Cq = 31.5), while its highest was in T2 (mean Cq = 35.1).

**Figure 6.**
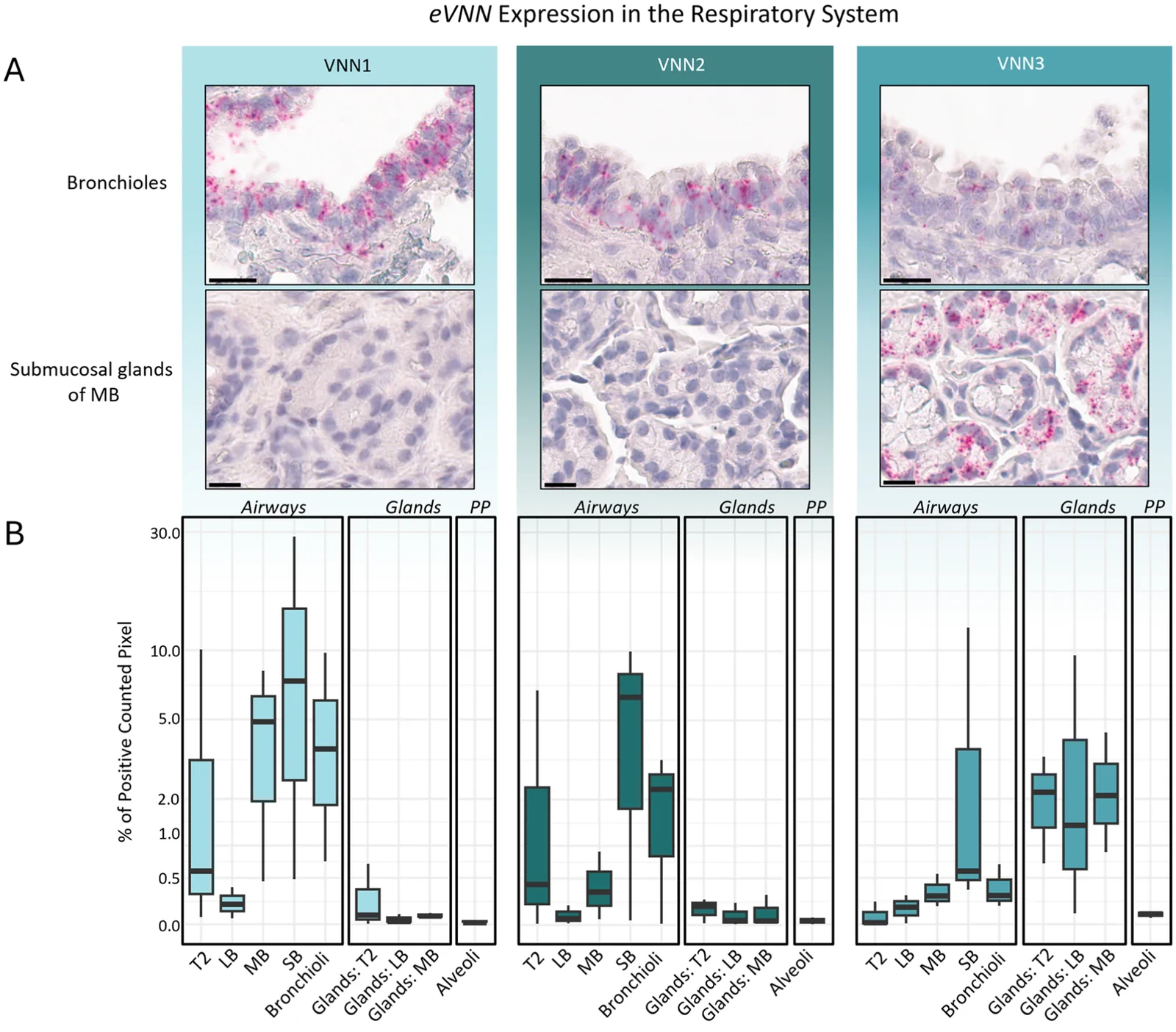
Cellular expression pattern of *eVNN* homologs in the airways. Cellular mRNA expression across various localizations of the airways, submucosal glands, and pulmonary parenchyma (PP) of *eVNN1, eVNN2*, and *eVNN3* was assessed by in situ hybridization (ISH). Analyzed regions of the airways included epithelium of the middle of the trachea (T2), large bronchi (LB), medium-sized bronchi (MB), small bronchi (SB), and bronchioli. Submucosal glands were analyzed for T2, LB, and MB. Regions predominantly containing alveoli were analyzed for pulmonary parenchyma. (A) Representative images of the cellular expression patterns are shown for bronchioles and submucosal glands of (MB). ISH signal in red, hematoxylin counterstaining. Scale bars: 20 µm. (B) Data were quantified using a pixel classifier in QuPath. Percentages of positively detected pixels were plotted using a log_(x+1)_ transformation for visualization purposes on the y-axis. Boxes represent the interquartile range, and horizontal lines indicate the median (*n*=3).

The *VNN*-expressing cell types in the respiratory tract were identified using in situ hybridization (ISH) and mRNA was semi-quantified using the QuPath software (Fig. 6). *eVNN1* and *eVNN2* signals were predominantly detected in respiratory epithelium, including ciliated epithelial cells and goblet cells of T2, LB, MB, SB, and bronchioles. In contrast, submucosal glands across all analyzed regions exhibited markedly lower levels of *eVNN1* and *eVNN2. eVNN3*, on the other hand, showed the highest expression in submucosal glands throughout the entire respiratory tract. Compared with *eVNN1* and *eVNN2*, its expression in respiratory epithelial cells was low (Fig. 6).

Interestingly, scattered *eVNN2* signals were found in intravascular monocytes (S5). However, due to their low frequency, these signals fell below the quantification limit. Type I and type II pneumocytes, smooth muscle cells, and fibrocytes did not show detectable expression of any *eVNN*. These findings are consistent with RT-qPCR analyses, which indicated higher *VNN* mRNA expression in samples composed primarily of respiratory mucosa compared with samples composed primarily of pulmonary parenchyma. Consistently across both methods, one horse (H2) showed no detectable *eVNN2* expression by RT-qPCR and only minimal expression by ISH.

### Pulmonary eVNN1 Is a Functional Pantetheinase

**Figure 7.**
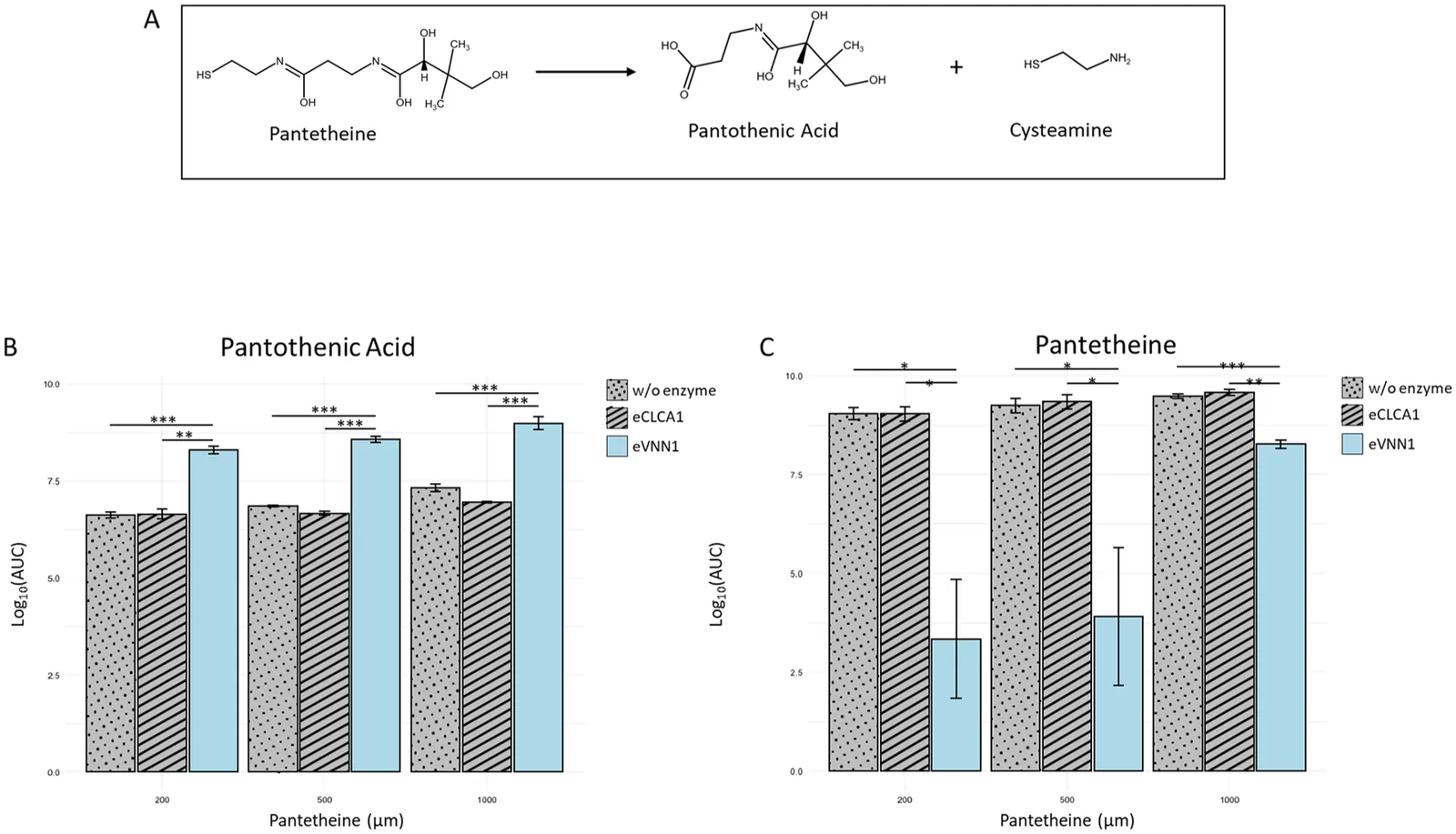
Pantetheinase activity of *eVNN1*. (A) Schematic representation of the enzymatic hydrolysis of pantetheine to pantothenic acid and cysteamine. Pantothenic acid (B) and pantetheine (C) concentrations measured by LC-MS/MS. Enzymatic hydrolysis of pantetheine to pantothenic acid was measured by LC-MS/MS. eVNN1 (blue bar), eCLCA (hatched bar), or reaction without (w/o) enzyme (stippled bar) were incubated with 0 µM, 200 µM, 500 µM, or 1 mM of pantetheine. Incubation of eVNN1 with pantetheine resulted in a decrease in pantetheine (C) and a corresponding increase in pantothenic acid (B), suggesting hydrolytic activity of eVNN1. In the reactions incubated with eCLCA1 or without any enzyme, no significant decrease in pantetheine (C) or increase in pantothenic acid (B) was observed. Asterisks indicate significance (***p<0.001, **p<0.01, *p<0.05) between reactions with and without eVNN1.

Next, we tested whether airway-expressed recombinant eVNN1 encodes a functional pantetheinase, hydrolyzing pantetheine to produce pantothenic acid and cysteamine (Fig. 7A). Recombinant, affinity-purified eVNN1 was incubated with its substrate pantetheine. LC-MS/MS analysis revealed a significant increase of 1.65 *log_10_ AUC* to 2.03 *log_10_ AUC* in pantothenic acid concentrations in reactions containing eVNN1, depending on the concentration of its substrate pantetheine, compared with both control conditions (Fig. 7B). Correspondingly, a significant decrease of 1.21 *log_10_ AUC* to 5.7 *log_10_ AUC* in pantetheine levels was observed in these samples, consistent with enzymatic conversion of pantetheine to pantothenic acid (Fig. 7C). The absolute values of each sample are presented in Supplementary Table S6. Cysteamine was not detectable, which may be attributed to its known chemical instability and a poor pharmacokinetic profile (*T*_1/2_ = 1.75 hr).^41^ Taken together, the observed substrate depletion and product accumulation demonstrate that the eVNN1 homolog is a functionally active pantetheinase.

## Discussion

This study provides the first comprehensive molecular and expression analysis of the equine *VNN* family, focusing on the respiratory tract. A member of this family, the VNN1 protein, was recently identified to be significantly increased in the airway mucus of horses with SEA compared with healthy controls.^8^ This suggests that the molecule may be relevant to the pathogenesis of EA. Basic knowledge of the *VNN* family is so far available for humans and mice, and data from murine asthma models and human asthma suggest that *VNN1* contributes to the pathophysiology of asthma.^10,19–23,42,43^ However, fundamental work is currently lacking for horses.

Three *eVNN* genes were identified in the equine genome. A comparison of the *VNN* family in different mammalian species on the genomic level reveals both conserved and species-specific features. The genomic location of this family between the flanking genes *SLC18B1* and *TAAR1* is a conserved feature of humans, mice, and horses. However, the number of functional proteins differs among the species. All three equine paralogs encode putatively functional proteins, whereas in humans, *VNN3* is a pseudogene, and mice lack a *Vnn2* ortholog.^20–23^

At the protein level, all homologs share similar structural and functional elements across all analyzed species, but their final protein localization appears to differ. In silico and in vitro analyses showed that eVNN1 is a secreted protein. In contrast, human and murine VNN1 are GPI-anchored proteins on the plasma membrane.^16,21,23^ This is a prominent difference between the species with a putative mechanistic, species-specific physiological or pathophysiological effect. For VNN2, a GPI-anchor motif is present in both eVNN2 and human VNN2.^44^ Our in vitro studies confirmed that this member of the equine VNN family is not released by the cells, whereas the human VNN2 had been detected in the culture medium of activated neutrophils and in synovial fluid of patients with rheumatoid arthritis.^45^ Future work must show if this is truly an interspecies difference or if the protein may be released from its GPI anchor under certain conditions, independent of the species. No interspecies differences were apparent for VNN3. In both mice and horses, the protein lacks a GPI anchor or TMD,^20^ and the secretory property was confirmed for equine VNN3.

The mRNA tissue expression patterns of the equine VNNs across different organs were distinct among the three paralogs, consistent with prior findings in human and murine VNNs (S7).^12,20,22^ In respiratory tissue, all *VNNs* across humans, mice, and horses were found to be expressed. In humans and mice, there is little detailed information about cell-specific localization in the respiratory tract. Human and murine *VNN1* are commonly described as an epithelial enzyme,^13,16,23,46,47^ but epithelial localization in the respiratory tract has only been confirmed in human nasal epithelial cells and, in vitro, in alveolar type II epithelial cells.^19,48^ Human VNN2 has been reported to be expressed in peripheral blood monocytes,^49,50^ but its expression in respiratory epithelial cells has not yet been analyzed. Murine *Vnn3* has been detected in alveolar type II epithelial cells (in vitro), nasal epithelial cells, peripheral monocytes, and granulocytes, but not in respiratory submucosal glandular cells.^20,48,51^

In horses, *eVNN1* and *eVNN2* were predominantly identified in the tracheal and bronchial epithelial cells, whereas *eVNN3* was largely restricted to airway submucosal glandular cells. Considering both their cellular expression and final protein localization, the three *eVNN* members seem to function in different microenvironments of the equine respiratory tract. As a secreted protein, *eVNN1* seems to perform its function within the airway lumen, whereas *eVNN2* acts on the interface between plasma membrane of respiratory epithelial cells and the airway lumen. *eVNN3* putatively functions within the lumen of airway submucosal glands. In addition to glandular cells, *eVNN2* was occasionally detected in another ontogenetically distinct cell type, blood monocytes, as previously reported for its human ortholog.^49,50^ These findings indicate that both stationary airway tissue cells and mobile immune cells appear to express *eVNNs*, which must be considered when interpreting their functions, particularly under pathophysiological conditions of the respiratory tract in horses. Although the horses used in this study showed no gross or histopathological changes indicative of respiratory disease, subclinical asthma or asthma in remission cannot be ruled out. Future studies should evaluate VNN expression in airway tissue in horses with MEA, SEA, and healthy controls to interpret the effects of VNN expression in disease pathophysiology.

Our expression analyses coincidentally revealed another interesting finding: While *eVNN1* and *eVNN3* displayed consistent expression profiles across all horses investigated, *eVNN2* expression was absent or very low in two (H2 and H9) out of nine horses in the analyzed tissues. The underlying mechanism responsible for this lack of expression in single individuals without any obvious pathological consequences for the horses is yet unclear. One explanation is the presence of naturally occurring genetic variants, such as deletion mutations, a phenomenon that has been reported in other gene families.^52^ Future studies should address whether specific genetic alterations are responsible for the absence of *eVNN2* expression and characterize their potential functional implications.

VNNs are pantetheinases, which catalyze the hydrolysis of pantetheine to cysteamine and pantothenic acid, a precursor of coenzyme A.^16,53–55^ In humans and mice, it is generally accepted that the role of the enzyme VNN1 is related to coenzyme A metabolism, lipid metabolism, and energy production, as well as regulation of oxidative stress and inflammation.^9,11,56^ Pantetheinase activity has also been demonstrated in equine kidney, however, without identifying the responsible enzyme.^53^ In this study, corresponding functional domains associated with pantetheinase activity were identified in silico in the equine VNNs, and pantetheinase activity was experimentally validated for eVNN1. These data suggest that eVNNs may also be linked to certain pantetheinase-associated metabolic pathways within their respective tissue microenvironment in the equine respiratory tract.

In the respiratory system, the enzymatic product of VNNs, cysteamine, has gained attention in the therapy of cystic fibrosis due to its mucolytic, antimicrobial, and anti-inflammatory properties.^57,58^ Its mucolytic activity has been experimentally demonstrated in cultured normal human bronchial epithelial cells.^58^ In murine asthma models, cysteamine prevented asthma development and reduced airway hyperresponsiveness, yielding results comparable to dexamethasone treatment.^18^ The same study reported that cysteamine decreased Th1, Th2, Th17, and effector T cells, along with eosinophil and neutrophil counts.^18^ In addition, treatment with cysteamine resulted in reduced airway hypersensitivity, inflammatory cell recruitment, and T helper cell differentiation by inhibition of transglutaminase 2.^59^ To conclude, cysteamine seems to offer a wide range of therapeutic effects and side effects, several of which may influence asthma pathogenesis also in horses. eVNN1 had been abundantly detected in horses affected by SEA.^8^ Due to the enzymatic activity of eVNN1, horses with SEA may therefore exhibit increased levels of cysteamine and pantothenic acid. These products may be involved in metabolic or inflammatory pathways underlying asthma pathogenesis. Future studies should therefore quantify and compare pantothenic acid and cysteamine concentrations in pulmonary tissue or airway fluids of asthmatic, including SEA and MEA, and healthy horses, and further characterize the role of eVNN1 and its enzymatic products in EA.

The presence of *VNN* genes in the respiratory tract, especially of functional *VNN1* in respiratory epithelium, underscores its potential involvement in airway diseases or regulatory mechanisms in the airways. Several findings from humans and murine models show that VNN1, in addition to its enzymatic function, has a role in a variety of pulmonary conditions, including asthma, chronic obstructive pulmonary disease, and sepsis-induced lung injury.^10,19,42,43^ In pediatric asthma, VNN1 has emerged as a potential biomarker for corticosteroid treatment responsiveness.^19^ This association is further supported by findings from a murine asthma model, in which *Vnn1*^−/−^ mice were resistant to dexamethasone treatment, indicating a functional role for VNN1 in steroid responsiveness.^19^ In humans, steroid resistance is often observed in non-T2, neutrophilic asthma.^60^ SEA is also characterized by increased neutrophilia, but typically responds well to corticosteroid treatment, with few reported exceptions.^2,61^ Further research is necessary to evaluate the potential role of VNN1 in the immune response to corticosteroid treatment in asthma in the respective species. Moreover, asthma development is frequently linked to intrauterine growth restriction (IUGR) in humans.^62^ In a murine IUGR model, *Vnn1* was significantly elevated in asthmatic IUGR mice and putatively induced asthma occurrence through activation of the PI3K/Akt/NFκB signaling pathway.^10^ Furthermore, a positive correlation between *VNN1* expression and DNA methylation at promoter CpG sites was observed in IUGR asthmatic mice as well as children with pediatric asthma, suggesting an epigenetic mechanism regulating *VNN1* expression.^10,19^ Evaluation of epigenetic mechanisms, such as DNA methylation, leading to *eVNN* expression and asthma occurrence in horses is currently unknown. However, a putative role in this context and in the context of asthma progression and steroid responsiveness should be addressed in horses in the future.

This study provides a detailed molecular characterization and spatial distribution in the airways of the eVNN family, revealing both conserved and species-specific features between horses, human, and mice. eVNN1 and eVNN3 are secreted glycoproteins with a predominant role in epithelial and glandular regions, respectively, while eVNN2 is a membrane-associated protein predominantly expressed in respiratory epithelium. The distinct expression patterns and cellular localization of the *eVNNs* suggest a functional diversification, particularly in the respiratory tract. Pantetheinase activity of eVNN1 was experimentally validated, implicating a putative role in asthma pathogenesis. These findings form the basis for future studies on the role of eVNNs in EA.

## Supplemental Material

sj-docx-1-jhc-10.1369_00221554261474565 – Supplemental material for Ortholog-specific Spatial Expression of Vanins in the Respiratory Tract of Horses Supplemental material, sj-docx-1-jhc-10.1369_00221554261474565 for Ortholog-specific Spatial Expression of Vanins in the Respiratory Tract of Horses by Katharina Landmann, Florian Bartenschlager, David Meierhofer, Andreas Spree, Caroline Pisch, Annette Zeyner, Christiane L. Schnabel and Lars Mundhenk in Journal of Histochemistry & Cytochemistry

sj-docx-2-jhc-10.1369_00221554261474565 – Supplemental material for Ortholog-specific Spatial Expression of Vanins in the Respiratory Tract of Horses Supplemental material, sj-docx-2-jhc-10.1369_00221554261474565 for Ortholog-specific Spatial Expression of Vanins in the Respiratory Tract of Horses by Katharina Landmann, Florian Bartenschlager, David Meierhofer, Andreas Spree, Caroline Pisch, Annette Zeyner, Christiane L. Schnabel and Lars Mundhenk in Journal of Histochemistry & Cytochemistry

sj-docx-3-jhc-10.1369_00221554261474565 – Supplemental material for Ortholog-specific Spatial Expression of Vanins in the Respiratory Tract of Horses Supplemental material, sj-docx-3-jhc-10.1369_00221554261474565 for Ortholog-specific Spatial Expression of Vanins in the Respiratory Tract of Horses by Katharina Landmann, Florian Bartenschlager, David Meierhofer, Andreas Spree, Caroline Pisch, Annette Zeyner, Christiane L. Schnabel and Lars Mundhenk in Journal of Histochemistry & Cytochemistry

sj-docx-4-jhc-10.1369_00221554261474565 – Supplemental material for Ortholog-specific Spatial Expression of Vanins in the Respiratory Tract of Horses Supplemental material, sj-docx-4-jhc-10.1369_00221554261474565 for Ortholog-specific Spatial Expression of Vanins in the Respiratory Tract of Horses by Katharina Landmann, Florian Bartenschlager, David Meierhofer, Andreas Spree, Caroline Pisch, Annette Zeyner, Christiane L. Schnabel and Lars Mundhenk in Journal of Histochemistry & Cytochemistry

sj-docx-5-jhc-10.1369_00221554261474565 – Supplemental material for Ortholog-specific Spatial Expression of Vanins in the Respiratory Tract of Horses Supplemental material, sj-docx-5-jhc-10.1369_00221554261474565 for Ortholog-specific Spatial Expression of Vanins in the Respiratory Tract of Horses by Katharina Landmann, Florian Bartenschlager, David Meierhofer, Andreas Spree, Caroline Pisch, Annette Zeyner, Christiane L. Schnabel and Lars Mundhenk in Journal of Histochemistry & Cytochemistry

sj-docx-6-jhc-10.1369_00221554261474565 – Supplemental material for Ortholog-specific Spatial Expression of Vanins in the Respiratory Tract of Horses Supplemental material, sj-docx-6-jhc-10.1369_00221554261474565 for Ortholog-specific Spatial Expression of Vanins in the Respiratory Tract of Horses by Katharina Landmann, Florian Bartenschlager, David Meierhofer, Andreas Spree, Caroline Pisch, Annette Zeyner, Christiane L. Schnabel and Lars Mundhenk in Journal of Histochemistry & Cytochemistry

sj-docx-7-jhc-10.1369_00221554261474565 – Supplemental material for Ortholog-specific Spatial Expression of Vanins in the Respiratory Tract of Horses Supplemental material, sj-docx-7-jhc-10.1369_00221554261474565 for Ortholog-specific Spatial Expression of Vanins in the Respiratory Tract of Horses by Katharina Landmann, Florian Bartenschlager, David Meierhofer, Andreas Spree, Caroline Pisch, Annette Zeyner, Christiane L. Schnabel and Lars Mundhenk in Journal of Histochemistry & Cytochemistry

sj-docx-8-jhc-10.1369_00221554261474565 – Supplemental material for Ortholog-specific Spatial Expression of Vanins in the Respiratory Tract of Horses Supplemental material, sj-docx-8-jhc-10.1369_00221554261474565 for Ortholog-specific Spatial Expression of Vanins in the Respiratory Tract of Horses by Katharina Landmann, Florian Bartenschlager, David Meierhofer, Andreas Spree, Caroline Pisch, Annette Zeyner, Christiane L. Schnabel and Lars Mundhenk in Journal of Histochemistry & Cytochemistry

## References

[1] Mims JW. Asthma: definitions and pathophysiology. Int Forum Allergy Rhinol. 2015;5(Suppl. 1):S2–6. doi:10.1002/alr.21609. 26335832

[2] Couetil L Cardwell JM Leguillette R Mazan M Richard E Bienzle D Bullone M Gerber V Ivester K Lavoie JP Martin J Moran G Niedźwiedź A Pusterla N Swiderski C. Equine asthma: current understanding and future directions. Front Vet Sci. 2020;7:450. doi:10.3389/fvets.2020.00450. 32903600 PMC7438831

[3] Wasko AJ Barkema HW Nicol J Fernandez N Logie N Léguillette R. Evaluation of a risk-screening questionnaire to detect equine lung inflammation: results of a large field study. Equine Vet J. 2011;43(2):145–52. doi:10.1111/j.2042-3306.2010.00150.x. 21592207

[4] Davis KU Sheats MK. Bronchoalveolar lavage cytology characteristics and seasonal changes in a herd of pastured teaching horses. Front Vet Sci. 2019;6:74. doi:10.3389/fvets.2019.00074. 30923711 PMC6426765

[5] Woodrow JS Sheats MK Cooper B Bayless R. Asthma: the use of animal models and their translational utility. Cells. 2023;12(7):1091. doi:10.3390/cells12071091. 37048164 PMC10093022

[6] Couëtil LL Cardwell JM Gerber V Lavoie JP Léguillette R Richard EA. Inflammatory airway disease of horses–revised consensus statement. J Vet Intern Med. 2016;30(2):503–15. doi:10.1111/jvim.13824. PMC4913592 26806374

[7] Bond S Léguillette R Richard EA Couetil L Lavoie JP Martin JG Pirie RS. Equine asthma: integrative biologic relevance of a recently proposed nomenclature. J Vet Intern Med. 2018;32(6):2088–98. doi:10.1111/jvim.15302. PMC6271326 30294851

[8] Bartenschlager F Kuropka B Schmitz P Dumke F Landmann K Gruber AD Weise C Schnabel CL Gehlen H Mundhenk L. Proteomic profiling of equine airway mucus reveals compositional changes in asthmatic phenotypes. Sci Rep. 2026;16(1):5880. doi:10.1038/s41598-026-38766-3. 41667845 PMC12894910

[9] Kaskow BJ Proffitt JM Blangero J Moses EK Abraham LJ. Diverse biological activities of the vascular non-inflammatory molecules–the Vanin pantetheinases. Biochem Biophys Res Commun. 2012;417(2):653–8. doi:10.1016/j.bbrc.2011.11.099. PMC3259148 22155241

[10] Xing Y Wei H Xiao X Chen Z Liu H Tong X Zhou W. Methylated Vnn1 at promoter regions induces asthma occurrence via the PI3K/Akt/NFkappaB-mediated inflammation in IUGR mice. Biol Open. 2020;9(4):bio049106. doi:10.1242/bio.049106. PMC7197710 32139393

[11] Bartucci R Salvati A Olinga P Boersma YL. Vanin 1: its physiological function and role in diseases. Int J Mol Sci. 2019;20(16):3891. doi:10.3390/ijms20163891. 31404995 PMC6719204

[12] Jansen PA Kamsteeg M Rodijk-Olthuis D van Vlijmen-Willems IM de Jongh GJ Bergers M Tjabringa GS Zeeuwen PL Schalkwijk J. Expression of the Vanin gene family in normal and inflamed human skin: induction by proinflammatory cytokines. J Invest Dermatol. 2009;129(9):2167–74. doi:10.1038/jid.2009.67. 19322213

[13] Berruyer C Martin FM Castellano R Macone A Malergue F Garrido-Urbani S Millet V Imbert J Duprè S Pitari G Naquet P Galland F. Vanin-1-/- mice exhibit a glutathione-mediated tissue resistance to oxidative stress. Mol Cell Biol. 2004;24(16):7214–24. doi:10.1128/MCB.24.16.7214-7224.2004. PMC479710 15282320

[14] Besouw M Masereeuw R van den Heuvel L Levtchenko E. Cysteamine: an old drug with new potential. Drug Discov Today. 2013;18(15–16):785–92. doi:10.1016/j.drudis.2013.02.003. 23416144

[15] Jeitner TM Lawrence DA. Mechanisms for the cytotoxicity of cysteamine. Toxicol Sci. 2001;63(1):57–64. doi:10.1093/toxsci/63.1.57. 11509744

[16] Pitari G Malergue F Martin F Philippe JM Massucci MT Chabret C Maras B Dupre S Naquet P Galland F. Pantetheinase activity of membrane-bound Vanin-1: lack of free cysteamine in tissues of Vanin-1 deficient mice. FEBS Lett. 2000;483(2–3):149–54. doi:10.1016/s0014-5793(00)02110-4. 11042271

[17] Lebo RV Kredich NM. Inactivation of human gamma-glutamylcysteine synthetase by cystamine. Demonstration and quantification of enzyme-ligand complexes. J Biol Chem. 1978;253(8):2615–3. doi:10.1016/S0021-9258(17)40865-9. 24639

[18] Bolcas PE Brandt EB Ruff BP Kalra M Khurana Hershey GK. Cysteamine prevents asthma development and reduces airway hyperresponsiveness in experimental asthma. Allergy. 2020;75(10):2675–7. doi:10.1111/all.14332. 32311100

[19] Xiao C Biagini Myers JM Ji H Metz K Martin LJ Lindsey M He H Powers R Ulm A Ruff B Ericksen MB Somineni HK Simmons J Strait RT Kercsmar CM Khurana Hershey GK. Vanin-1 expression and methylation discriminate pediatric asthma corticosteroid treatment response. J Allergy Clin Immunol. 2015;136(4):923–31. doi:10.1016/j.jaci.2015.01.045. PMC4586304 25910714

[20] Martin F Malergue F Pitari G Philippe JM Philips S Chabret C Granjeaud S Mattei MG Mungall AJ Naquet P Galland F. Vanin genes are clustered (human 6q22-24 and mouse 10A2B1) and encode isoforms of pantetheinase ectoenzymes. Immunogenetics. 2001;53(4):296–306. doi:10.1007/s002510100327. 11491533

[21] Granjeaud S Naquet P Galland F. An ESTs description of the new Vanin gene family conserved from fly to human. Immunogenetics. 1999;49(11–12):964–72. doi:10.1007/s002510050580. 10501839

[22] Galland F Malergue F Bazin H Mattei MG Aurrand-Lions M Theillet C Naquet P. Two human genes related to murine Vanin-1 are located on the long arm of human chromosome 6. Genomics. 1998;53(2):203–13. doi:10.1006/geno.1998.5481. 9790769

[23] Aurrand-Lions M Galland F Bazin H Zakharyev VM Imhof BA Naquet P. Vanin-1, a novel GPI-linked perivascular molecule involved in thymus homing. Immunity. 1996;5(5):391–405. doi:10.1016/s1074-7613(00)80496-3. 8934567

[24] Sayers EW Beck J Bolton EE Brister JR Chan J Connor R Feldgarden M Fine AM Funk K Hoffman J Kannan S Kelly C Klimke W Kim S Lathrop S Marchler-Bauer A Murphy TD O’Sullivan C Schmieder E Skripchenko Y Stine A Thibaud-Nissen F Wang J Ye J Zellers E Schneider VA Pruitt KD. Database resources of the National Center for Biotechnology Information in 2025. Nucleic Acids Res. 2025;53(D1):D20–9. doi:10.1093/nar/gkae979. PMC11701734 39526373

[25] Dyer SC Austine-Orimoloye O Azov AG Barba M Barnes I Barrera-Enriquez VP Becker A Bennett R Beracochea M Berry A Bhai J Bhurji SK Boddu S Branco Lins PR Brooks L Ramaraju SB Campbell LI Martinez MC Charkhchi M Cortes LA Davidson C Denni S Dodiya K Donaldson S El Houdaigui B El Naboulsi T Falola O Fatima R Genez T Martinez JG Gurbich T Hardy M Hollis Z Hunt T Kay M Kaykala V Lemos D Lodha D Mathlouthi N Merino GA Merritt R Mirabueno LP Mushtaq A Hossain SN Pérez-Silva JG Perry M Piližota I Poppleton D Prosovetskaia I Raj S Salam AIA Saraf S Saraiva-Agostinho N Sinha S Sipos B Sitnik V Steed E Suner M-M Surapaneni L Sutinen K Tricomi FF Tsang I Urbina-Gómez D Veidenberg A Walsh TA Willhoft NL Allen J Alvarez-Jarreta J Chakiachvili M Cheema J Da Rocha JB Silva NH de Giorgetti S Haggerty L Ilsley GR Keatley J Loveland JE Moore B Mudge JM Naamati G Tate J Trevanion SJ Winterbottom A Flint B Frankish A Hunt SE Finn RD Freeberg MA Harrison PW Martin FJ Yates AD. Ensembl 2025. Nucleic Acids Res. 2025;53(D1):D948–57. doi:10.1093/nar/gkae1071. PMC11701638 39656687

[26] Altschul SF Gish W Miller W Myers EW Lipman DJ. Basic local alignment search tool. J Mol Biol. 1990;215(3):403–10. doi:10.1016/S0022-2836(05)80360-2s. 2231712

[27] Madeira F Madhusoodanan N Lee J Eusebi A Niewielska A Tivey ARN Lopez R Butcher S. The EMBL-EBI Job Dispatcher sequence analysis tools framework in 2024. Nucleic Acids Res. 2024;52(W1):W521–5. doi:10.1093/nar/gkae241. PMC11223882 38597606

[28] Hirokawa T Boon-Chieng S Mitaku S. SOSUI: classification and secondary structure prediction system for membrane proteins. Bioinformatics. 1998;14(4):378–9. doi:10.1093/bioinformatics/14.4.378. 9632836

[29] Pierleoni A Martelli PL Casadio R. PredGPI: a GPI-anchor predictor. BMC Bioinformatics. 2008;9:392. doi:10.1186/1471-2105-9-392. 18811934 PMC2571997

[30] Gupta R Brunak S . Prediction of glycosylation across the human proteome and the correlation to protein function. Pac Symp Biocomput. 2002:310–22. 11928486

[31] Almagro Armenteros JJ Tsirigos KD Sønderby CK Petersen TN Winther O Brunak S Heijne G von Nielsen H . SignalP 5.0 improves signal peptide predictions using deep neural networks. Nat Biotechnol. 2019;37(4):420–3. doi:10.1038/s41587-019-0036-z. 30778233

[32] Edgar RC. MUSCLE: multiple sequence alignment with high accuracy and high throughput. Nucleic Acids Res. 2004;32(5):1792–7. doi:10.1093/nar/gkh340. PMC390337 15034147

[33] Tamura K Stecher G Kumar S. MEGA11: molecular evolutionary genetics analysis version 11. Mol Biol Evol. 2021;38(7):3022–7. doi:10.1093/molbev/msab120. PMC8233496 33892491

[34] Anton F Leverkoehne I Mundhenk L Thoreson WB Gruber AD. Overexpression of eCLCA1 in small airways of horses with recurrent airway obstruction. J Histochem Cytochem. 2005;53(8):1011–21. doi:10.1369/jhc.4A6599.2005. PMC1383431 15879574

[35] Ruan BH Cole DC Wu P Quazi A Page K Wright JF Huang N Stock JR Nocka K Aulabaugh A Krykbaev R Fitz LJ Wolfman NM Fleming ML. A fluorescent assay suitable for inhibitor screening and Vanin tissue quantification. Anal Biochem. 2010;399(2):284–92. doi:10.1016/j.ab.2009.12.010. 20018163

[36] Ye J Coulouris G Zaretskaya I Cutcutache I Rozen S Madden TL. Primer-BLAST: a tool to design target-specific primers for polymerase chain reaction. BMC Bioinformatics. 2012;13:134. doi:10.1186/1471-2105-13-134. 22708584 PMC3412702

[37] Bartenschlager F Klymiuk N Weise C Kuropka B Gruber AD Mundhenk L. Evolutionarily conserved properties of CLCA proteins 1, 3 and 4, as revealed by phylogenetic and biochemical studies in avian homologues. PLoS ONE. 2022;17(4):e0266937. doi:10.1371/journal.pone.0266937. PMC9007345 35417490

[38] Schmidbauer S-M Venner M Samson-Himmelstjerna G von Drommer W Gruber AD. Compensated overexpression of procollagens alpha 1(I) and alpha 1(III) following perilla mint ketone-induced acute pulmonary damage in horses. J Comp Pathol. 2004;131(2–3):186–98. doi:10.1016/j.jcpa.2004.03.005. 15276858

[39] Bankhead P Loughrey MB Fernández JA Dombrowski Y McArt DG Dunne PD McQuaid S Gray RT Murray LJ Coleman HG James JA Salto-Tellez M Hamilton PW. QuPath: open source software for digital pathology image analysis. Sci Rep. 2017;7(1):16878. doi:10.1038/s41598-017-17204-5. PMC5715110 29203879

[40] Udenfriend S Kodukula K. How glycosylphosphatidylinositol-anchored membrane proteins are made. Annu Rev Biochem. 1995;64:563–91. doi:10.1146/annurev.bi.64.070195.003023. 7574493

[41] Atallah C Charcosset C Greige-Gerges H. Challenges for cysteamine stabilization, quantification, and biological effects improvement. J Pharm Anal. 2020;10(6):499–516. doi:10.1016/j.jpha.2020.03.007. PMC7775854 33425447

[42] Zhang X Cong W Lu A. Vanin-1 as a novel biomarker for chronic obstructive pulmonary disease. Heart Lung. 2022;56:91–5. doi:10.1016/j.hrtlng.2022.06.022. 35810677

[43] Ling L Lu HT Wang HF Shen MJ Zhang HB. MicroRNA-203 Acts as a Potent Suppressor in Septic Shock by Alleviating Lung Injury via Inhibition of VNN1. Kidney Blood Press Res. 2019;44(4):565–82. doi:10.1159/000500484. 31340209

[44] Suzuki K Watanabe T Sakurai S Ohtake K Kinoshita T Araki A Fujita T Takei H Takeda Y Sato Y Yamashita T Araki Y Sendo F. A novel glycosylphosphatidyl inositol-anchored protein on human leukocytes: a possible role for regulation of neutrophil adherence and migration. J Immunol. 1999;162(7):4277–84. 10201959

[45] Huang J Takeda Y Watanabe T Sendo F. A sandwich ELISA for detection of soluble GPI-80, a Glycosylphosphatidyl-Inositol (GPI)-anchored protein on human leukocytes involved in regulation of neutrophil adherence and migration—its release from activated neutrophils and presence in synovial fluid. Microbiol Immunol. 2001;45(6):467–71. doi:10.1111/j.1348-0421.2001.tb02646.x. 11497222

[46] Gensollen T Bourges C Rihet P Rostan A Millet V Noguchi T Bourdon V Sobol H Dubuquoy L Bertin B Fumery M Desreumaux P Colombel JF Chamaillard M Hebuterne X Hofman P Naquet P Galland F. Functional polymorphisms in the regulatory regions of the VNN1 gene are associated with susceptibility to inflammatory bowel diseases. Inflamm Bowel Dis. 2013;19(11):2315–5. doi:10.1097/MIB.0b013e3182a32b03. 23949622

[47] Pouyet L Roisin-Bouffay C Clément A Millet V Garcia S Chasson L Issaly N Rostan A Hofman P Naquet P Galland F. Epithelial Vanin-1 controls inflammation-driven carcinogenesis in the colitis-associated colon cancer model. Inflamm Bowel Dis. 2010;16(1):96–104. doi:10.1002/ibd.21031. 19572375

[48] Xu Y Saegusa C Schehr A Grant S Whitsett JA Ikegami M. C/EBP{alpha} is required for pulmonary cytoprotection during hyperoxia. Am J Physiol Lung Cell Mol Physiol. 2009;297(2):L286–98. doi:10.1152/ajplung.00094.2009. PMC2742785 19465518

[49] Bahabayi A Alimu X Wang G Gao Y Chen Y Zhao J Lian X Li Q Xiong Z Zhang Z Wang P Liu C. VNN2-expressing circulating monocytes exhibit unique functional characteristics and are decreased in patients with primary Sjögren’s syndrome. J Autoimmun. 2024;147:103275. doi:10.1016/j.jaut.2024.103275. 38936146

[50] Bornhauser B Cario G Rinaldi A Risch T Rodriguez Martinez V Schutte M Warnatz HJ Scheidegger N Mirkowska P Temperli M Moller C Schumich A Dworzak M Attarbaschi A Bruggemann M Ritgen M Mejstrikova E Hofmann A Buldini B Scarparo P Basso G Maglia O Gaipa G Skroblyn TL Ngo Q Te Kronnie G Vendramini E Panzer-Grumayer R Barz MJ Marovca B Hauri-Hohl M Niggli F Eckert C Schrappe M Stanulla M Zimmermann M Wollscheid B Yaspo ML Bourquin JP. The hematopoietic stem cell marker VNN2 is associated with chemoresistance in pediatric B-cell precursor ALL. Blood Adv. 2020;4(17):4052–64. doi:10.1182/bloodadvances.2019000938. PMC7479947 32853382

[51] Huang S-Y Chen Y-Y Huang D Wu C-D Lin C-T. Abstract 5356: the role of VNN3 gene in nasopharyngeal carcinoma tumorigenesis. Cancer Res. 2014;74(Suppl. 19):5356. doi:10.1158/1538-7445.AM2014-5356.

[52] Plog S Mundhenk L Klymiuk N Gruber AD. Genomic, tissue expression, and protein characterization of pCLCA1, a putative modulator of cystic fibrosis in the pig. J Histochem Cytochem. 2009;57(12):1169–81. doi:10.1369/jhc.2009.954594. PMC2778090 19755716

[53] Duprè S Graziani MT Rosei MA Fabi A Del Grosso E. The enzymatic breakdown of pantethine to pantothenic acid and cystamine. Eur J Biochem. 1970;16(3):571–8. doi:10.1111/j.1432-1033.1970.tb01119.x. 5477303

[54] Wittwer CT Burkhard D Ririe K Rasmussen R Brown J Wyse BW Hansen RG. Purification and properties of a pantetheine-hydrolyzing enzyme from pig kidney. J Biol Chem. 1983;258(16):9733–8. doi:10.1016/S0021-9258(17)44559-5. 6885768

[55] Maras B Barra D Duprè S Pitari G. Is pantetheinase the actual identity of mouse and human Vanin-1 proteins? FEBS Letters. 1999;461(3):149–52. doi:10.1016/s0014-5793(99)01439-8. 10567687

[56] Nitto T Onodera K. Linkage between coenzyme a metabolism and inflammation: roles of pantetheinase. J Pharmacol Sci. 2013;123(1):1–8. doi:10.1254/jphs.13r01cp. 23978960

[57] Devereux G Steele S Griffiths K Devlin E Fraser-Pitt D Cotton S Norrie J Chrystyn H O’Neil D. An open-label investigation of the pharmacokinetics and tolerability of oral cysteamine in adults with cystic fibrosis. Clin Drug Investig. 2016;36(8):605–12. doi:10.1007/s40261-016-0405-z. PMC4951511 27153825

[58] Charrier C Rodger C Robertson J Kowalczuk A Shand N Fraser-Pitt D Mercer D O’Neil D. Cysteamine (Lynovex(R)), a novel mucoactive antimicrobial & antibiofilm agent for the treatment of cystic fibrosis. Orphanet J Rare Dis. 2014;9:189. doi:10.1186/s13023-014-0189-2. 25433388 PMC4260250

[59] Oh K Seo MW Lee GY Byoun OJ Kang HR Cho SH Lee DS. Airway epithelial cells initiate the allergen response through transglutaminase 2 by inducing IL-33 expression and a subsequent Th2 response. Respir Res. 2013;14(1):35. doi:10.1186/1465-9921-14-35. 23496815 PMC3602182

[60] Xie Y Abel PW Casale TB Tu Y. T(H)17 cells and corticosteroid insensitivity in severe asthma. J Allergy Clin Immunol. 2022;149(2):467–79. doi:10.1016/j.jaci.2021.12.769. PMC8821175 34953791

[61] Simoes J Batista M Tilley P. The immune mechanisms of severe equine asthma-current understanding and what is missing. Animals (Basel). 2022;12(6):744. doi:10.3390/ani12060744. 35327141 PMC8944511

[62] Wang KCW James AL Noble PB . Fetal growth restriction and asthma: is the damage done? Physiology (Bethesda). 2021;36(4):256–66. doi:10.1152/physiol.00042.2020. 34159809

