# Supplementary material for: Ortholog-specific Spatial Expression of Vanins in the Respiratory Tract of Horses
Source: J Histochem Cytochem. 2026 Aug 31:00221554261474565. Online ahead of print. doi: 10.1369/00221554261474565 (PMC13530036; doi:10.1369/00221554261474565)
Supplement: Supplementary file 1 [file sj-docx-1-jhc-10.1369_00221554261474565.docx]

## Animals, tissue samples and methods used

### Table 1) Metadata of Horses Included in the Study

| **Horse** | **ID-Number*** | **Age** | **Gender** | **Weight** | **Breed** |
| --- | --- | --- | --- | --- | --- |
| **H1** | P1 | 9 | Gelding | 443 kg | Warmblood |
| **H2** | P4 | 13 | Gelding | 622 kg | Warmblood |
| **H3** | P7 | 5 | Gelding | 493 kg | Warmblood |
| **H4** | P11 | 4 | Mare | 440 kg | Warmblood |
| **H5** | P14 | 6 | Mare | 455 kg | Warmblood |
| **H6** | P15 | 10 | Gelding | 485 kg | Warmblood |
| **H7** | S529/20 | 25 | Gelding | 600 kg | Warmblood |
| **H8** | S655/20 | 30 | Gelding | 250 kg | Pony |
| **H9** | S727/20 | 15 | Stallion | Unknown | Warmblood |
| **H10** | S760/20 | 22 | Mare | Unknown | Warmblood |

**Horses H1-H6 were sampled with permission from local authorities (State Office of Health and Social affairs, Saxony, Germany; approval number TVV63/21). Numbers in this row represent identification (ID) numbers assigned for the local authorities. Horses H7-H10 were sampled during routine pathological diagnostics in the Institute for Veterinary Pathology of the Freie Universität Berlin; State Office of Health and Social Affairs Berlin StN 010/24) Numbers in this row represent internal case numbers.*

### Table 2) Respiratory Tissue

|  | **Animals** | | | | | |
| --- | --- | --- | --- | --- | --- | --- |
| **Tissue Samples** | **H1** | **H2** | **H3** | **H4** | **H5** | **H6** |
| Trachea, cranial (T1) |  | qPCR | qPCR | qPCR | qPCR | qPCR |
| Trachea, middle (T2) |  | qPCR, ISH | qPCR, ISH | qPCR, ISH | qPCR | qPCR |
| Trachea, caudal (T3) |  | qPCR | qPCR | qPCR | qPCR | qPCR |
| Large bronchi (LB) |  | qPCR, ISH | qPCR, ISH | qPCR, ISH | qPCR | qPCR |
| Medium-sized bronchi (MB) |  | qPCR, ISH | qPCR, ISH | qPCR, ISH | qPCR, cloning | qPCR |
| Small bronchi (SB) |  | qPCR, ISH | qPCR, ISH | qPCR, ISH | qPCR | qPCR |
| Cranial pulmonary parenchyma (L1) |  | qPCR, ISH | qPCR, ISH | qPCR, ISH | qPCR | qPCR |
| Caudal pulmonary parenchyma (L2) | ORF-Sequencing (PCR-product) | qPCR | qPCR | qPCR | qPCR | qPCR |

| Tissue | Animals | | | |
| --- | --- | --- | --- | --- |
|  | **H7** | **H8** | **H9** | **H10** |
| Skin | qPCR | qPCR | qPCR | qPCR |
| Skeletal muscle | qPCR | qPCR | qPCR | qPCR |
| Conjunctiva | qPCR | qPCR | qPCR | qPCR |
| Liver | qPCR | qPCR | qPCR | qPCR |
| Spleen | qPCR | qPCR | qPCR | qPCR |
| Kidneys | qPCR | qPCR | qPCR | qPCR |
| Urinary bladder | qPCR | qPCR | qPCR | qPCR |
| Adrenal glands | qPCR | qPCR | qPCR | qPCR |
| Pancreas | qPCR | qPCR | qPCR | qPCR |
| Stomach | qPCR | qPCR | qPCR | qPCR |
| Duodenum | qPCR | qPCR | qPCR | qPCR |
| Jejunum | qPCR | qPCR | qPCR | qPCR |
| Caecum | qPCR | qPCR | qPCR | qPCR |
| Colon | qPCR | qPCR | qPCR | qPCR |
| Rectum | qPCR | qPCR | qPCR | qPCR |
| Aorta | qPCR | qPCR | qPCR | qPCR |
| Right heart ventricle | qPCR | qPCR | qPCR | qPCR |
| Right atrium | qPCR | qPCR | qPCR | qPCR |
| Thyroid glands | qPCR | qPCR | qPCR | qPCR |
| Cerebrum | qPCR | qPCR | qPCR | qPCR |
| Cerebellum | qPCR | qPCR | qPCR | qPCR |
| Pituitary gland | qPCR | qPCR | qPCR | qPCR |

### Table 3) Extra-Respiratory Tissue
