# Supplementary material for: Ortholog-specific Spatial Expression of Vanins in the Respiratory Tract of Horses
Source: J Histochem Cytochem. 2026 Aug 31:00221554261474565. Online ahead of print. doi: 10.1369/00221554261474565 (PMC13530036; doi:10.1369/00221554261474565)
Supplement: Supplementary file 2 [file sj-docx-2-jhc-10.1369_00221554261474565.docx]

## Primersequences

### Table 4) Primers Used for ORF Amplification and RT-qPCR

|  | **Forward (5´ - 3´)** | **Reverse (5´ - 3´)** |
| --- | --- | --- |
| **ORF amplification** | | |
| **eVNN1 (ORF)** | *cgg*GGTACCgccgccaccATGATTTCTTCTCAG*CTGCTCGC* | *tgc*TCTAGAATCTCCTAATCTGTCCTGGTC |
| **eVNN2 (ORF)** | *gg*GGTACCgccgccaccATGATTGCTTCCTCT | *ccgc*TCTAGACAGTATCTGCCAAGCTATGAT |
| **eVNN3 (ORF)** | *gg*GGTACCgccgccaccATGATTACATCATAT | *gc*TCTAGAATCTTGAAAAGGTGCGAGTGAAGG |
| **RTqPCR** | | |
| **eVNN1** | GGCACGCTACCATAAGCAAAA | CAGGGTAACAGCAGGGTTGT |
| **eVNN2** | GCGGCTGTTTTTGCCGTAAT | TGATTTGAGCACCCTGCTTTG |
| **eVNN3** | GGCGCGCTACCATAAGTACA | ACTCATTGACCACCACCACG |
| **EF1a** | CAAAAACGACCCACCAATGG | GGCCTGGATGGTTCAGGATA |

*Primers used for ORF amplification: lower case and cursive: linker sequence; underlined: restriction sites (KpnI: GGTACC, XbaI: TCTAGA); lowercase: Kozak sequence; uppercase: targeted gene sequence.*
