# Supplementary material for: Ortholog-specific Spatial Expression of Vanins in the Respiratory Tract of Horses
Source: J Histochem Cytochem. 2026 Aug 31:00221554261474565. Online ahead of print. doi: 10.1369/00221554261474565 (PMC13530036; doi:10.1369/00221554261474565)
Supplement: Supplementary file 3 [file sj-docx-3-jhc-10.1369_00221554261474565.docx]

# Cloned Sequences

## VNN1(X4) clone XM_023651108.2 pcDNA 3.1

GTTAGGCGTTTTGCGCTGCTTCGCGATGTACGGGCCAGATATACGCGTTGACATTGATTATTGACTAGTTATTAATAGTAATCAATTACGGGGTCATTAGTTCATAGCCCATATATGGAGTTCCGCGTTACATAACTTACGGTAAATGGCCCGCCTGGCTGACCGCCCAACGACCCCCGCCCATTGACGTCAATAATGACGTATGTTCCCATAGTAACGCCAATAGGGACTTTCCATTGACGTCAATGGGTGGAGTATTTACGGTAAACTGCCCACTTGGCAGTACATCAAGTGTATCATATGCCAAGTACGCCCCCTATTGACGTCAATGACGGTAAATGGCCCGCCTGGCATTATGCCCAGTACATGACCTTATGGGACTTTCCTACTTGGCAGTACATCTACGTATTAGTCATCGCTATTACCATGGTGATGCGGTTTTGGCAGTACATCAATGGGCGTGGATAGCGGTTTGACTCACGGGGATTTCCAAGTCTCCACCCCATTGACGTCAATGGGAGTTTGTTTTGGCACCAAAATCAACGGGACTTTCCAAAATGTCGTAACAACTCCGCCCCATTGACGCAAATGGGCGGTAGGCGTGTACGGTGGGAGGTCTATATAAGCAGAGCTCGTTTAGTGAACCGTCAGATCGCCTGGAGACGCCATCCACGCTGTTTTGACCTCCATAGAAGACACCGGGACCGATCCAGCCTCCGGACTCTAGAGGATCGAACCCTTAAGCTTGCTAGCGTTTAAACTCCTGCAAAAGGCACGTCGGTACCGGATCCCCATTTTTTTGGCTCGAGCGGCCGCCCCACGTGGAATTCAGCGCCGCCACCATGATTTCTTCTCAGCTGCTCGCTTATGTGGCAATTTTGGTTTTCTGCACCTTCCAAGCCCACTCCCTGGAGACCTTTATTGCAGCTGTTTACGAGCACGCAGTGATATTGCCCAATGCCACCCTAACGCCAGTGTCCCGGGAAGAGGCGTTGCCATTAATGAACCGCAATCTGGATCTTTTGGAGGGAGCAATCACCTCAGCAGCCAAGCAGGGTGCGCACATCATTGTGACTCCAGAAGATGGTGTTTACGGCTGGAACTTCAGCAGGGACACGCTCTATCCGTACTTGGAGGACATCCCAGACCCTGCAGTGAACTGGATCCCTTGCAACAATCCCGACAGATTTGGCCACACTCCAGTCCAAGAGAGGCTCAGCTGCCTGGCCAAGGACAACTCTATCTACGTCGTGGCAAATATCGGGGACAAGAAGCCATGCAATGCCAGTGACCCTGAGTGCCCCCCTGATGGCCGTTACCAGTACAACACTGATGTGGTGTTTGATGCTCAGGGAAAACTTGTGGCACGCTACCATAAGCAAAACCTTTTCATGGGTGAAGATCAATTCAATGCACCCAAGGAGCCTGAGGTCGTGACTTTCAACACCACCTTTGGAAAGTTTGGCATTTTCACGTGCTTCGATATTCTCTTCTACGACCCTGCTGTTACCCTGGTGACAGATTTCCAAGTGGACACCATCCTCTTCCCGACAGCCTGGATGAATGTTTTGCCACATTTGTCAGCTATTGAATTTCACTCGGCTTGGGCTATGGGCATGGGGGTCAATTTCCTTGCATCCAATATACATTACCCCTCAATGAAAATGACAGGAAGTGGCATCTATGCTCCTGATTCTCCAAGAGCATTTCATTATGATATGAAGACGGAGGAGGGAAAACTACTCCTCTCACAGCTGGACTCCCACCCGCACCACCCAGCAGTGGTGAACTGGACTTCTTTTGCCAGTGGTGTGAAAACACTCTCAACGGGAAACCAGGAATTTAACAGCACTGTCTTTTTTGATGAATTCACCTTCCTGGAGCTCACCGAAGTCACAGGAAATTACACAGTTTGTCAGAAAGCTCTCTGCTGTCACCTAAGCTACAAGATGTCTGAGAAGAGATCAGATGAAGTGTACGCCCTAGGGGCATTTGATGGCTTACATACTGTTGAAGGGAGTTATTATTTACAGGTTTGCACTCTGTTGAAGTGTAGAACCACGGACTTACACACTTGTGGTGACTCGGTGGAAACTGCTTCCACTAGGTTCGATACGTTCTCCCTCAGTGGCACTTTTGGGACCCAGCATGTCTTCCCTGAGGTGCTGCTGAGTGAAATTCAGCTCGCACCTGGAGAATTCCAGGTGTCAAGTGATGGACGCTTGTTCAGTCTGCAGCCCCCCTCGGGACCTGTCCTGACAGTGACTCTTTATGGGAGGCTGTACGAGAAGGACCAGGTATCCGACGCCTCATCAGACCTCACAGCACGAGCACTGAGAGACAGATTAGGAGATGCTGAGAACCTGTACTTCCAAGGCCACCACCACCATCACCACTGGTCCCATCCTCAGTTCGAGAAAGGCGGAGGATCTGGCGGTGGTTCTGGTGGATCTGCTTGGAGCCATCCACAGTTTGAAAAGTGAAGATCTCCGCGGGGCCGGCCGGGCCCAAAAGGGTTCGATCCCTACCGGTTAGTAATGAGTTTGATATCTCGACAATCAACCTCTGGATTACAAAATTTGTGAAAGATTGACTGGTATTCTTAACTATGTTGCTCCTTTTACGCTATGTGGATACGCTGCTTTAATGCCTTTGTATCATGCTATTGCTTCCCGTATGGCTTTCATTTTCTCCTCCTTGTATAAATCCTGGTTGCTGTCTCTTTATGAGGAGTTGTGGCCCGTTGTCAGGCAACGTGGCGTGGTGTGCACTGTGTTTGCTGACGCAACCCCCACTGGTTGGGGCATTGCCACCACCTGTCAGCTCCTTTCCGGGACTTTCGCTTTCCCCCTCCCTATTGCCACGGCGGAACTCATCGCCGCCTGCCTTGCCCGCTGCTGGACAGGGGCTCGGCTGTTGGGCACTGACAATTCCGTGGTGTTGTCGGGGAAGCTGACGTCCTTTCCATGGCTGCTCGCCTGTGTTGCCACCTGGATTCTGCGCGGGACGTCCTTCTGCTACGTCCCTTCGGCCCTCAATCCAGCGGACCTTCCTTCCCGCGGCCTGCTGCCGGCTCTGCGGCCTCTTCCGCGTCTTCGCCTTCGCCCTCAGACGAGTCGGATCTCCCTTTGGGCCGCCTCCCCGCCTGGAACGGGGGAGGCTAACTGAAACACGGAAGGAGACAATACCGGAAGGAACCCGCGCTATGACGGCAATAAAAAGACAGAATAAAACGCACGGGTGTTGGGTCGTTTGTTCATAAACGCGGGGTTCGGTCCCAGGGCTGGCACTCTGTCGATACCCCACCGAGACCCCATTGGGGCCAATACGCCCGCGTTTCTTCCTTTTCCCCACCCCACCCCCCAAGTTCGGGTGAAGGCCCAGGGCTCGCAGCCAACGTCGGGGCGGCAGGCCCTGCCATAGCAGATCTGCGCAGCTGGGGCTCTAGGGGGTATCCCCACGCGCCCTGTAGCGGCGCATTAAGCGCGGCGGGTGTGGTGGTTACGCGCAGCGTGACCGCTACACTTGCCAGCGCCCTAGCGCCCGCTCCTTTCGCTTTCTTCCCTTCCTTTCTCGCCACGTTCGCCGGCTTTCCCCGTCAAGCTCTAAATCGGGGGCTCCCTTTAGGGTTCCGATTTAGTGCTTTACGGCACCTCGACCCCAAAAAACTTGATTAGGGTGATGGTTCACGTAGTGGGCCATCGCCCTGATAGACGGTTTTTCGCCCTTTGACGTTGGAGTCCACGTTCTTTAATAGTGGACTCTTGTTCCAAACTGGAACAACACTCAACCCTATCTCGGTCTATTCTTTTGATTTATAAGGGATTTTGCCGATTTCGGCCTATTGGTTAAAAAATGAGCTGATTTAACAAAAATTTAACGCGAATTAATTCTGTGGAATGTGTGTCAGTTAGGGTGTGGAAAGTCCCCAGGCTCCCCAGCAGGCAGAAGTATGCAAAGCATGCATCTCAATTAGTCAGCAACCAGGTGTGGAAAGTCCCCAGGCTCCCCAGCAGGCAGAAGTATGCAAAGCATGCATCTCAATTAGTCAGCAACCATAGTCCCGCCCCTAACTCCGCCCATCCCGCCCCTAACTCCGCCCAGTTCCGCCCATTCTCCGCCCCATGGCTGACTAATTTTTTTTATTTATGCAGAGGCCGAGGCCGCCTCTGCCTCTGAGCTATTCCAGAAGTAGTGAGGAGGCTTTTTTGGAGGCCTAGGCTTTTGCAAAAAGCTCCCGGGAGCTTGTATATCCATTTTCGGATCTGATCAAGAGACAGGATGAGGATCGTTTCGCATGATTGAACAAGATGGATTGCACGCAGGTTCTCCGGCCGCTTGGGTGGAGAGGCTATTCGGCTATGACTGGGCACAACAGACAATCGGCTGCTCTGATGCCGCCGTGTTCCGGCTGTCAGCGCAGGGGCGCCCGGTTCTTTTTGTCAAGACCGACCTGTCCGGTGCCCTGAATGAACTGCAGGACGAGGCAGCGCGGCTATCGTGGCTGGCCACGACGGGCGTTCCTTGCGCAGCTGTGCTCGACGTTGTCACTGAAGCGGGAAGGGACTGGCTGCTATTGGGCGAAGTGCCGGGGCAGGATCTCCTGTCATCTCACCTTGCTCCTGCCGAGAAAGTATCCATCATGGCTGATGCAATGCGGCGGCTGCATACGCTTGATCCGGCTACCTGCCCATTCGACCACCAAGCGAAACATCGCATCGAGCGAGCACGTACTCGGATGGAAGCCGGTCTTGTCGATCAGGATGATCTGGACGAAGAGCATCAGGGGCTCGCGCCAGCCGAACTGTTCGCCAGGCTCAAGGCGCGCATGCCCGACGGCGAGGATCTCGTCGTGACCCATGGCGATGCCTGCTTGCCGAATATCATGGTGGAAAATGGCCGCTTTTCTGGATTCATCGACTGTGGCCGGCTGGGTGTGGCGGACCGCTATCAGGACATAGCGTTGGCTACCCGTGATATTGCTGAAGAGCTTGGCGGCGAATGGGCTGACCGCTTCCTCGTGCTTTACGGTATCGCCGCTCCCGATTCGCAGCGCATCGCCTTCTATCGCCTTCTTGACGAGTTCTTCTGAGCGGGACTCTGGGGTTCGCGAAATGACCGACCAAGCGACGCCCAACCTGCCATCACGAGATTTCGATTCCACCGCCGCCTTCTATGAAAGGTTGGGCTTCGGAATCGTTTTCCGGGACGCCGGCTGGATGATCCTCCAGCGCGGGGATCTCATGCTGGAGTTCTTCGCCCACCCCAACTTGTTTATTGCAGCTTATAATGGTTACAAATAAAGCAATAGCATCACAAATTTCACAAATAAAGCATTTTTTTCACTGCATTCTAGTTGTGGTTTGTCCAAACTCATCAATGTATCTTATCATGTCTGTATACCGTCGACCTCTAGCTAGAGCTTGGCGTAATCATGGTCATAGCTGTTTCCTGTGTGAAATTGTTATCCGCTCACAATTCCACACAACATACGAGCCGGAAGCATAAAGTGTAAAGCCTGGGGTGCCTAATGAGTGAGCTAACTCACATTAATTGCGTTGCGCTCACTGCCCGCTTTCCAGTCGGGAAACCTGTCGTGCCAGCTGCATTAATGAATCGGCCAACGCGCGGGGAGAGGCGGTTTGCGTATTGGGCGCTCTTCCGCTTCCTCGCTCACTGACTCGCTGCGCTCGGTCGTTCGGCTGCGGCGAGCGGTATCAGCTCACTCAAAGGCGGTAATACGGTTATCCACAGAATCAGGGGATAACGCAGGAAAGAACATGTGAGCAAAAGGCCAGCAAAAGGCCAGGAACCGTAAAAAGGCCGCGTTGCTGGCGTTTTTCCATAGGCTCCGCCCCCCTGACGAGCATCACAAAAATCGACGCTCAAGTCAGAGGTGGCGAAACCCGACAGGACTATAAAGATACCAGGCGTTTCCCCCTGGAAGCTCCCTCGTGCGCTCTCCTGTTCCGACCCTGCCGCTTACCGGATACCTGTCCGCCTTTCTCCCTTCGGGAAGCGTGGCGCTTTCTCATAGCTCACGCTGTAGGTATCTCAGTTCGGTGTAGGTCGTTCGCTCCAAGCTGGGCTGTGTGCACGAACCCCCCGTTCAGCCCGACCGCTGCGCCTTATCCGGTAACTATCGTCTTGAGTCCAACCCGGTAAGACACGACTTATCGCCACTGGCAGCAGCCACTGGTAACAGGATTAGCAGAGCGAGGTATGTAGGCGGTGCTACAGAGTTCTTGAAGTGGTGGCCTAACTACGGCTACACTAGAAGAACAGTATTTGGTATCTGCGCTCTGCTGAAGCCAGTTACCTTCGGAAAAAGAGTTGGTAGCTCTTGATCCGGCAAACAAACCACCGCTGGTAGCGGTGGTTTTTTTGTTTGCAAGCAGCAGATTACGCGCAGAAAAAAAGGATCTCAAGAAGATCCTTTGATCTTTTCTACGGGGTCTGACGCTCAGTGGAACGAAAACTCACGTTAAGGGATTTTGGTCATGAGATTATCAAAAAGGATCTTCACCTAGATCCTTTTAAATTAAAAATGAAGTTTTAAATCAATCTAAAGTATATATGAGTAAACTTGGTCTGACAGTTACCAATGCTTAATCAGTGAGGCACCTATCTCAGCGATCTGTCTATTTCGTTCATCCATAGTTGCCTGACTCCCCGTCGTGTAGATAACTACGATACGGGAGGGCTTACCATCTGGCCCCAGTGCTGCAATGATACCGCGAGACCCACGCTCACCGGCTCCAGATTTATCAGCAATAAACCAGCCAGCCGGAAGGGCCGAGCGCAGAAGTGGTCCTGCAACTTTATCCGCCTCCATCCAGTCTATTAATTGTTGCCGGGAAGCTAGAGTAAGTAGTTCGCCAGTTAATAGTTTGCGCAACGTTGTTGCCATTGCTACAGGCATCGTGGTGTCACGCTCGTCGTTTGGTATGGCTTCATTCAGCTCCGGTTCCCAACGATCAAGGCGAGTTACATGATCCCCCATGTTGTGCAAAAAAGCGGTTAGCTCCTTCGGTCCTCCGATCGTTGTCAGAAGTAAGTTGGCCGCAGTGTTATCACTCATGGTTATGGCAGCACTGCATAATTCTCTTACTGTCATGCCATCCGTAAGATGCTTTTCTGTGACTGGTGAGTACTCAACCAAGTCATTCTGAGAATAGTGTATGCGGCGACCGAGTTGCTCTTGCCCGGCGTCAATACGGGATAATACCGCGCCACATAGCAGAACTTTAAAAGTGCTCATCATTGGAAAACGTTCTTCGGGGCGAAAACTCTCAAGGATCTTACCGCTGTTGAGATCCAGTTCGATGTAACCCACTCGTGCACCCAACTGATCTTCAGCATCTTTTACTTTCACCAGCGTTTCTGGGTGAGCAAAAACAGGAAGGCAAAATGCCGCAAAAAAGGGAATAAGGGCGACACGGAAATGTTGAATACTCATACTCTTCCTTTTTCAATATTATTGAAGCATTTATCAGGGTTATTGTCTCATGAGCGGATACATATTTGAATGTATTTAGAAAAATAAACAAATAGGGGTTCCGCGCACATTTCCCCGAAAAGTGCCACCTGACGTCGACGGATCGGGAGATCTCCCGATCCCCTATGGTGCACTCTCAGTACAATCTGCTCTGATGCCGCATAGTTAAGCCAGTATCTGCTCCCTGCTTGTGTGTTGGAGGTCGCTGAGTAGTGCGCGAGCAAAATTTAAGCTACAACAAGGCAAGGCTTGACCGACAATTGCATGAAGAATCTGCTTAGG

## aa sequence of recombinant eVNN1 with hexahistidine–Twin-Strep tag

Translation 546 aa, MW=60617.112 Da:

MISSQLLAYVAILVFCTFQAHSLETFIAAVYEHAVILPNATLTPVSREEALPLMNRNLDLLEGAITSAAKQGAHIIVTPEDGVYGWNFSRDTLYPYLEDIPDPAVNWIPCNNPDRFGHTPVQERLSCLAKDNSIYVVANIGDKKPCNASDPECPPDGRYQYNTDVVFDAQGKLVARYHKQNLFMGEDQFNAPKEPEVVTFNTTFGKFGIFTCFDILFYDPAVTLVTDFQVDTILFPTAWMNVLPHLSAIEFHSAWAMGMGVNFLASNIHYPSMKMTGSGIYAPDSPRAFHYDMKTEEGKLLLSQLDSHPHHPAVVNWTSFASGVKTLSTGNQEFNSTVFFDEFTFLELTEVTGNYTVCQKALCCHLSYKMSEKRSDEVYALGAFDGLHTVEGSYYLQVCTLLKCRTTDLHTCGDSVETASTRFDTFSLSGTFGTQHVFPEVLLSEIQLAPGEFQVSSDGRLFSLQPPSGPVLTVTLYGRLYEKDQVSDASSDLTARALRDRLGDAENLYFQGHHHHHHWSHPQFEKGGGSGGGSGGSAWSHPQFEK

## VNN2 clone XM_070223887.1 pcDNA 3.4

GACGGATCGGGAGATCTCCCGATCCCCTATGGTGCACTCTCAGTACAATCTGCTCTGATGCCGCATAGTTAAGCCAGTATCTGCTCCCTGCTTGTGTGTTGGAGGTCGCTGAGTAGTGCGCGAGCAAAATTTAAGCTACAACAAGGCAAGGCTTGACCGACAATTGCATGAAGAATCTGCTTAGGGTTAGGCGTTTTGCGCTGCTTCGCGATGTACGGGCCAGATATACGCGTTGACATTGATTATTGACTAGTTATTAATAGTAATCAATTACGGGGTCATTAGTTCATAGCCCATATATGGAGTTCCGCGTTACATAACTTACGGTAAATGGCCCGCCTGGCTGACCGCCCAACGACCCCCGCCCATTGACGTCAATAATGACGTATGTTCCCATAGTAACGCCAATAGGGACTTTCCATTGACGTCAATGGGTGGAGTATTTACGGTAAACTGCCCACTTGGCAGTACATCAAGTGTATCATATGCCAAGTACGCCCCCTATTGACGTCAATGACGGTAAATGGCCCGCCTGGCATTATGCCCAGTACATGACCTTATGGGACTTTCCTACTTGGCAGTACATCTACGTATTAGTCATCGCTATTACCATGGTGATGCGGTTTTGGCAGTACATCAATGGGCGTGGATAGCGGTTTGACTCACGGGGATTTCCAAGTCTCCACCCCATTGACGTCAATGGGAGTTTGTTTTGGCACCAAAATCAACGGGACTTTCCAAAATGTCGTAACAACTCCGCCCCATTGACGCAAATGGGCGGTAGGCGTGTACGGTGGGAGGTCTATATAAGCAGAGCTCTCTGGCTAACTAGAGAACCCACTGCTTACTGGCTTATCGAAATTAATACGACTCACTATAGGGAGACCCAAGCTGGCTAGCGTTTAAACTTAAGCTTCACGTCAGCGCTTCCGGAGCGGCCGCCCTAAAAAAGGGGTACCgccgccaccATGATTGCTTCCTCTTTTCGAACCTCTGCGGCTGTTTTTGCCGTAATTATCCTGCATGTTGGGGCCCTGGATACTTTCATAGCTGCAGTTTATGAACACGCCGTCATACTGCCAAAAACAACAGTGACACCTGTCTCTCAGGACGATGCCTTGCTCCTCATGAACAAGAATATAGATATTCTGGAGAGAGAGATTAAGCAGGCAGCAAAGCAGGGTGCTCAAATCATTGTGACTCCAGAAGATGCCCTTTATGGTTGGACATTTACCAGGGAAACCATTTTCCCTTATCTGGAGGACGTCCCGGACCCTCAGGCGAACTGGATTCCGTGTCAGGACTCCAACAGATTTGGTTACACACCAGTACAAGTAAGACTCAGCTGCCTGGCCAAGAACAACTCAATCTATGTCTTGGCAAATATGGGGGACAAGAAGCCATGTAATCGCCATAACTCCATGTGTCCTCCCAATGGCTATTATCAATACAACACCAATGTGGTATATGATGCGGAGGGGAAACTGGTAGCACGCTACCATAAGTACCACCTCTACTCTGAGCCTCAGTTTGATGTCCCTGAAAAGCCCGAGTTGGTGACTTTCAACACCACCTTTGGAAGGTTTGGCATTTTCACGTGCTTTGATATTTTCTTCCACGATCCTGCTGTCACCCTGGTGAAAGATTTCCATGTGGATACCATCCTGTTTCCCACAGCTTGGATGAATGTCTTGCCCCTTCTGACAGCTATTGAATTCCACTCGGCTTGGGCTGTGGGCATGAGAGTTAATCTTCTTGCGGCCAACACCCACAATATCCACATGAATATGACAGGCAGCGGTATCTATGCACCACAGTCTCCCAAAGTATACCACCATGACATGGAGACAGAGTCGGGAAAGCTCCTCCTTGCGGAAGTGGATTCGCATCCCCGAGACTCTCCTGCCTACCCAACAGCTGTGAACTGGAGTGCCCATGCCACAACCATCAGACCATTCCCAGGAAAGAAAAACACCTTCCGGGCATTTATTTCCCGGGACGCTTTCAACTTCACAGAACTTTTTGAAAATGCAGGAAACCTTACAGTCTGTCAAAAAGATCTCTGCTGTCATTTAAGCTACAGGATGTTAGGAAAGGAAGAGGATGAAGTGTATGCTCTGGGAGCTTTTTCAGGACTTCACGGCCGAAGGAGAAAAGAGTACTGGCAGGTGTGCACAATGCTGAAATGCAAAACTACTAATTTGACAACTTGTGGGCGGCCGGTAGAAACTGCTTCTACAAGATTTGAAATGTTCTCCTTAAGTGGCACATTTGGAACAGAGTATGTCATTCCAGAAGTGCTACTTACTAAAATTCACCTGTCACCTGGAAAATTTGAGGTACTACAAGATGGGCGTTTGGTGAACAAGAACGGACCATCCGAGCCTATCCTGACAGTGTCTCTCTTTGGGAGATGGTATGCTAAGGATTCATCTTCCATCCCAGGCGGAACCAGCAATTCAGCAACAACTTACCTGCTGATACCCATTTTATTGATGATCATAGCTTGGCAGATACTGTCTAGAGAGAACCTGTACTTCCAAGGCCACCACCACCATCACCACTGGTCCCATCCTCAGTTCGAGAAAGGCGGAGGATCTGGCGGTGGTTCTGGTGGATCTGCTTGGAGCCATCCACAGTTTGAAAAGTGAAGATCTCCGCGGGGCCGGCCGGGCCCGTTTAAACCCGCTGATCAGCCTCGACTGTGCCTTCTAGTTGCCAGCCATCTGTTGTTTGCCCCTCCCCCGTGCCTTCCTTGACCCTGGAAGGTGCCACTCCCACTGTCCTTTCCTAATAAAATGAGGAAATTGCATCGCATTGTCTGAGTAGGTGTCATTCTATTCTGGGGGGTGGGGTGGGGCAGGACAGCAAGGGGGAGGATTGGGAAGACAATAGCAGGCATGCTGGGGATGCGGTGGGCTCTATGGCTTCTGAGGCGGAAAGAACCAGCTGGGGCTCTAGGGGGTATCCCCACGCGCCCTGTAGCGGCGCATTAAGCGCGGCGGGTGTGGTGGTTACGCGCAGCGTGACCGCTACACTTGCCAGCGCCCTAGCGCCCGCTCCTTTCGCTTTCTTCCCTTCCTTTCTCGCCACGTTCGCCGGCTTTCCCCGTCAAGCTCTAAATCGGGGGCTCCCTTTAGGGTTCCGATTTAGTGCTTTACGGCACCTCGACCCCAAAAAACTTGATTAGGGTGATGGTTCACGTAGTGGGCCATCGCCCTGATAGACGGTTTTTCGCCCTTTGACGTTGGAGTCCACGTTCTTTAATAGTGGACTCTTGTTCCAAACTGGAACAACACTCAACCCTATCTCGGTCTATTCTTTTGATTTATAAGGGATTTTGCCGATTTCGGCCTATTGGTTAAAAAATGAGCTGATTTAACAAAAATTTAACGCGAATTAATTCTGTGGAATGTGTGTCAGTTAGGGTGTGGAAAGTCCCCAGGCTCCCCAGCAGGCAGAAGTATGCAAAGCATGCATCTCAATTAGTCAGCAACCAGGTGTGGAAAGTCCCCAGGCTCCCCAGCAGGCAGAAGTATGCAAAGCATGCATCTCAATTAGTCAGCAACCATAGTCCCGCCCCTAACTCCGCCCATCCCGCCCCTAACTCCGCCCAGTTCCGCCCATTCTCCGCCCCATGGCTGACTAATTTTTTTTATTTATGCAGAGGCCGAGGCCGCCTCTGCCTCTGAGCTATTCCAGAAGTAGTGAGGAGGCTTTTTTGGAGGCCTAGGCTTTTGCAAAAAGCTCCCGGGAGCTTGTATATCCATTTTCGGATCTGATCAAGAGACAGGATGAGGATCGTTTCGCATGATTGAACAAGATGGATTGCACGCAGGTTCTCCGGCCGCTTGGGTGGAGAGGCTATTCGGCTATGACTGGGCACAACAGACAATCGGCTGCTCTGATGCCGCCGTGTTCCGGCTGTCAGCGCAGGGGCGCCCGGTTCTTTTTGTCAAGACCGACCTGTCCGGTGCCCTGAATGAACTGCAGGACGAGGCAGCGCGGCTATCGTGGCTGGCCACGACGGGCGTTCCTTGCGCAGCTGTGCTCGACGTTGTCACTGAAGCGGGAAGGGACTGGCTGCTATTGGGCGAAGTGCCGGGGCAGGATCTCCTGTCATCTCACCTTGCTCCTGCCGAGAAAGTATCCATCATGGCTGATGCAATGCGGCGGCTGCATACGCTTGATCCGGCTACCTGCCCATTCGACCACCAAGCGAAACATCGCATCGAGCGAGCACGTACTCGGATGGAAGCCGGTCTTGTCGATCAGGATGATCTGGACGAAGAGCATCAGGGGCTCGCGCCAGCCGAACTGTTCGCCAGGCTCAAGGCGCGCATGCCCGACGGCGAGGATCTCGTCGTGACCCATGGCGATGCCTGCTTGCCGAATATCATGGTGGAAAATGGCCGCTTTTCTGGATTCATCGACTGTGGCCGGCTGGGTGTGGCGGACCGCTATCAGGACATAGCGTTGGCTACCCGTGATATTGCTGAAGAGCTTGGCGGCGAATGGGCTGACCGCTTCCTCGTGCTTTACGGTATCGCCGCTCCCGATTCGCAGCGCATCGCCTTCTATCGCCTTCTTGACGAGTTCTTCTGAGCGGGACTCTGGGGTTCGAAATGACCGACCAAGCGACGCCCAACCTGCCATCACGAGATTTCGATTCCACCGCCGCCTTCTATGAAAGGTTGGGCTTCGGAATCGTTTTCCGGGACGCCGGCTGGATGATCCTCCAGCGCGGGGATCTCATGCTGGAGTTCTTCGCCCACCCCAACTTGTTTATTGCAGCTTATAATGGTTACAAATAAAGCAATAGCATCACAAATTTCACAAATAAAGCATTTTTTTCACTGCATTCTAGTTGTGGTTTGTCCAAACTCATCAATGTATCTTATCATGTCTGTATACCGTCGACCTCTAGCTAGAGCTTGGCGTAATCATGGTCATAGCTGTTTCCTGTGTGAAATTGTTATCCGCTCACAATTCCACACAACATACGAGCCGGAAGCATAAAGTGTAAAGCCTGGGGTGCCTAATGAGTGAGCTAACTCACATTAATTGCGTTGCGCTCACTGCCCGCTTTCCAGTCGGGAAACCTGTCGTGCCAGCTGCATTAATGAATCGGCCAACGCGCGGGGAGAGGCGGTTTGCGTATTGGGCGCTCTTCCGCTTCCTCGCTCACTGACTCGCTGCGCTCGGTCGTTCGGCTGCGGCGAGCGGTATCAGCTCACTCAAAGGCGGTAATACGGTTATCCACAGAATCAGGGGATAACGCAGGAAAGAACATGTGAGCAAAAGGCCAGCAAAAGGCCAGGAACCGTAAAAAGGCCGCGTTGCTGGCGTTTTTCCATAGGCTCCGCCCCCCTGACGAGCATCACAAAAATCGACGCTCAAGTCAGAGGTGGCGAAACCCGACAGGACTATAAAGATACCAGGCGTTTCCCCCTGGAAGCTCCCTCGTGCGCTCTCCTGTTCCGACCCTGCCGCTTACCGGATACCTGTCCGCCTTTCTCCCTTCGGGAAGCGTGGCGCTTTCTCATAGCTCACGCTGTAGGTATCTCAGTTCGGTGTAGGTCGTTCGCTCCAAGCTGGGCTGTGTGCACGAACCCCCCGTTCAGCCCGACCGCTGCGCCTTATCCGGTAACTATCGTCTTGAGTCCAACCCGGTAAGACACGACTTATCGCCACTGGCAGCAGCCACTGGTAACAGGATTAGCAGAGCGAGGTATGTAGGCGGTGCTACAGAGTTCTTGAAGTGGTGGCCTAACTACGGCTACACTAGAAGAACAGTATTTGGTATCTGCGCTCTGCTGAAGCCAGTTACCTTCGGAAAAAGAGTTGGTAGCTCTTGATCCGGCAAACAAACCACCGCTGGTAGCGGTGGTTTTTTTGTTTGCAAGCAGCAGATTACGCGCAGAAAAAAAGGATCTCAAGAAGATCCTTTGATCTTTTCTACGGGGTCTGACGCTCAGTGGAACGAAAACTCACGTTAAGGGATTTTGGTCATGAGATTATCAAAAAGGATCTTCACCTAGATCCTTTTAAATTAAAAATGAAGTTTTAAATCAATCTAAAGTATATATGAGTAAACTTGGTCTGACAGTTACCAATGCTTAATCAGTGAGGCACCTATCTCAGCGATCTGTCTATTTCGTTCATCCATAGTTGCCTGACTCCCCGTCGTGTAGATAACTACGATACGGGAGGGCTTACCATCTGGCCCCAGTGCTGCAATGATACCGCGAGACCCACGCTCACCGGCTCCAGATTTATCAGCAATAAACCAGCCAGCCGGAAGGGCCGAGCGCAGAAGTGGTCCTGCAACTTTATCCGCCTCCATCCAGTCTATTAATTGTTGCCGGGAAGCTAGAGTAAGTAGTTCGCCAGTTAATAGTTTGCGCAACGTTGTTGCCATTGCTACAGGCATCGTGGTGTCACGCTCGTCGTTTGGTATGGCTTCATTCAGCTCCGGTTCCCAACGATCAAGGCGAGTTACATGATCCCCCATGTTGTGCAAAAAAGCGGTTAGCTCCTTCGGTCCTCCGATCGTTGTCAGAAGTAAGTTGGCCGCAGTGTTATCACTCATGGTTATGGCAGCACTGCATAATTCTCTTACTGTCATGCCATCCGTAAGATGCTTTTCTGTGACTGGTGAGTACTCAACCAAGTCATTCTGAGAATAGTGTATGCGGCGACCGAGTTGCTCTTGCCCGGCGTCAATACGGGATAATACCGCGCCACATAGCAGAACTTTAAAAGTGCTCATCATTGGAAAACGTTCTTCGGGGCGAAAACTCTCAAGGATCTTACCGCTGTTGAGATCCAGTTCGATGTAACCCACTCGTGCACCCAACTGATCTTCAGCATCTTTTACTTTCACCAGCGTTTCTGGGTGAGCAAAAACAGGAAGGCAAAATGCCGCAAAAAAGGGAATAAGGGCGACACGGAAATGTTGAATACTCATACTCTTCCTTTTTCAATATTATTGAAGCATTTATCAGGGTTATTGTCTCATGAGCGGATACATATTTGAATGTATTTAGAAAAATAAACAAATAGGGGTTCCGCGCACATTTCCCCGAAAAGTGCCACCTGACGTC

## aa sequence of recombinant eVNN2 with hexahistidine–Twin-Strep tag

Translation 560 aa, MW=62888.7063 Da:

MIASSFRTSAAVFAVIILHVGALDTFIAAVYEHAVILPKTTVTPVSQDDALLLMNKNIDILEREIKQAAKQGAQIIVTPEDALYGWTFTRETIFPYLEDVPDPQANWIPCQDSNRFGYTPVQVRLSCLAKNNSIYVLANMGDKKPCNRHNSMCPPNGYYQYNTNVVYDAEGKLVARYHKYHLYSEPQFDVPEKPELVTFNTTFGRFGIFTCFDIFFHDPAVTLVKDFHVDTILFPTAWMNVLPLLTAIEFHSAWAVGMRVNLLAANTHNIHMNMTGSGIYAPQSPKVYHHDMETESGKLLLAEVDSHPRDSPAYPTAVNWSAHATTIRPFPGKKNTFRAFISRDAFNFTELFENAGNLTVCQKDLCCHLSYRMLGKEEDEVYALGAFSGLHGRRRKEYWQVCTMLKCKTTNLTTCGRPVETASTRFEMFSLSGTFGTEYVIPEVLLTKIHLSPGKFEVLQDGRLVNKNGPSEPILTVSLFGRWYAKDSSSIPGGTSNSATTYLLIPILLMIIAWQILSRENLYFQGHHHHHHWSHPQFEKGGGSGGGSGGSAWSHPQFEK

## VNN3 clone XM_001504346.5 pcDNA 3.4

GACGGATCGGGAGATCTCCCGATCCCCTATGGTGCACTCTCAGTACAATCTGCTCTGATGCCGCATAGTTAAGCCAGTATCTGCTCCCTGCTTGTGTGTTGGAGGTCGCTGAGTAGTGCGCGAGCAAAATTTAAGCTACAACAAGGCAAGGCTTGACCGACAATTGCATGAAGAATCTGCTTAGGGTTAGGCGTTTTGCGCTGCTTCGCGATGTACGGGCCAGATATACGCGTTGACATTGATTATTGACTAGTTATTAATAGTAATCAATTACGGGGTCATTAGTTCATAGCCCATATATGGAGTTCCGCGTTACATAACTTACGGTAAATGGCCCGCCTGGCTGACCGCCCAACGACCCCCGCCCATTGACGTCAATAATGACGTATGTTCCCATAGTAACGCCAATAGGGACTTTCCATTGACGTCAATGGGTGGAGTATTTACGGTAAACTGCCCACTTGGCAGTACATCAAGTGTATCATATGCCAAGTACGCCCCCTATTGACGTCAATGACGGTAAATGGCCCGCCTGGCATTATGCCCAGTACATGACCTTATGGGACTTTCCTACTTGGCAGTACATCTACGTATTAGTCATCGCTATTACCATGGTGATGCGGTTTTGGCAGTACATCAATGGGCGTGGATAGCGGTTTGACTCACGGGGATTTCCAAGTCTCCACCCCATTGACGTCAATGGGAGTTTGTTTTGGCACCAAAATCAACGGGACTTTCCAAAATGTCGTAACAACTCCGCCCCATTGACGCAAATGGGCGGTAGGCGTGTACGGTGGGAGGTCTATATAAGCAGAGCTCTCTGGCTAACTAGAGAACCCACTGCTTACTGGCTTATCGAAATTAATACGACTCACTATAGGGAGACCCAAGCTGGCTAGCGTTTAAACTTAAGCTTCACGTCAGCGCTTCCGGAGCGGCCGCCCTAAAAAAGGGGTACCGCCGCCACCATGATTACATCATATTTTCCAAAATGTGTGGCCGTTTTTGCCCTCTCTGTCCTGAGTGTCGGTGCGCTGGACACTTTTATTGCTGCAGTGTACGAGCATGCTGTTATATTACCAAACGGAACACAAACACCTGTTCCAAAAGAAGAGGCTTTGCTCCTGATGAACAAGAACATAGATGTTTTGGAGAAAGCAGTTAAACTGGCAGCCAGGCAGGGTGCACATATCATTGTGACCCCAGAAGATGGAATTTATGGCTGGGTCTTCACAAGGGAGACAATTTACCCCTACCTAGAGGATATCCCACACCCTGAAGTGAACTGGATTCCATGTAGAGACCCCCAGAGGTTTGGCCACACCCCAGTACAAGAGAGGCTCAGCTGCATGGCCAAGGACAACTCTATCTACGTCGTGGCAAATATTGGGGACAAGAAGCCATGCAATGCCAGTGACCCTGAGTGTCCCCCTGACGGTCGTTACCAGTACAACACTGACGTGGTGTTTGATTCTGAGGGCAGGCTGGTGGCGCGCTACCATAAGTACAATCTTTTTGCGCCTGAAATTCAATTTGATTTCCCCAAGGATTCAGAGTTCGTGACTTTTGACACTCCCTTTGGGAAGTTTGGCATTTTCACCTGCTTTGACATTTTTTCTCACGACCCGGCCGTGGTGGTGGTCAATGAGTTTCAGGTGGACAGCATCCTCTACCCGACGGCCTGGTACAACACGCTGCCCCTCCTCTCTGCTGTTCCCTTCCATTCGGCGTGGGCCAGAGCCATGAGGGTCAACCTGCTTGCCGCCAACACCCACAACACCAGCATGCACATGACCGGGAGTGGAATCTACGCACCGGAAGCAGTCAAGGTGTACTACTATGACATGGAAACGGAGAGTGGCCAGCTGATGCTCTCAGAGTTGAAGTCCCGGCCTCGGCGTGAAACCACCTACCCTGCAGCCGTTGACTGGAGTGCTTATGCCAGAGGCGTCAAGCCGTTCCCGTCTGAGCAACCGGATTTTCCAGGGATGATCTATTTTGATGAGTTCACCTTCACTGAGCTTCAGAAGAACACAGGAAATTACACAGTTTGTCAGAAAGACCTGTGTTGTCACTTAACTTACCAGATGTCTGAGAAGCGAACAGATGAGGTGTATGCGCTTGGGGCTTTTGACGGCCTGCACACAGTGGAAGGCCAGTACTACTTGCAGATATGCACATTACTCAAGTGTCAAACCACCGAGCTGAGAACTTGCGGGGAGCCTGCGGGGTCAGCGTTTACCAGGTTTGAAGAATTTTCCCTCAGCGGCACTTTCGTAACAAGTTACGTTTTCCCACAGCTCATTCTAAGTGGGAGTCAGCTTGCCCCGGAGAGACATTACGAGGTTTCACAAGACGGACGTCTGAGGAGCCGAGGCGGAGCCCCTTTGCCTGTCTTAGTGATGGCCCTGTATGGAAGAGTGTTTGAGAAGGACCCTCCGCGCTTAGGGCAGGGACCTGAGAAATTACAACGACCCCCTTCACTCGCACCTTTTCAAGATTCTAGAGAGAACCTGTACTTCCAAGGCCACCACCACCATCACCACTGGTCCCATCCTCAGTTCGAGAAAGGCGGAGGATCTGGCGGTGGTTCTGGTGGATCTGCTTGGAGCCATCCACAGTTTGAAAAGTGAAGATCTCCGCGGGGCCGGCCGGGCCCGTTTAAACCCGCTGATCAGCCTCGACTGTGCCTTCTAGTTGCCAGCCATCTGTTGTTTGCCCCTCCCCCGTGCCTTCCTTGACCCTGGAAGGTGCCACTCCCACTGTCCTTTCCTAATAAAATGAGGAAATTGCATCGCATTGTCTGAGTAGGTGTCATTCTATTCTGGGGGGTGGGGTGGGGCAGGACAGCAAGGGGGAGGATTGGGAAGACAATAGCAGGCATGCTGGGGATGCGGTGGGCTCTATGGCTTCTGAGGCGGAAAGAACCAGCTGGGGCTCTAGGGGGTATCCCCACGCGCCCTGTAGCGGCGCATTAAGCGCGGCGGGTGTGGTGGTTACGCGCAGCGTGACCGCTACACTTGCCAGCGCCCTAGCGCCCGCTCCTTTCGCTTTCTTCCCTTCCTTTCTCGCCACGTTCGCCGGCTTTCCCCGTCAAGCTCTAAATCGGGGGCTCCCTTTAGGGTTCCGATTTAGTGCTTTACGGCACCTCGACCCCAAAAAACTTGATTAGGGTGATGGTTCACGTAGTGGGCCATCGCCCTGATAGACGGTTTTTCGCCCTTTGACGTTGGAGTCCACGTTCTTTAATAGTGGACTCTTGTTCCAAACTGGAACAACACTCAACCCTATCTCGGTCTATTCTTTTGATTTATAAGGGATTTTGCCGATTTCGGCCTATTGGTTAAAAAATGAGCTGATTTAACAAAAATTTAACGCGAATTAATTCTGTGGAATGTGTGTCAGTTAGGGTGTGGAAAGTCCCCAGGCTCCCCAGCAGGCAGAAGTATGCAAAGCATGCATCTCAATTAGTCAGCAACCAGGTGTGGAAAGTCCCCAGGCTCCCCAGCAGGCAGAAGTATGCAAAGCATGCATCTCAATTAGTCAGCAACCATAGTCCCGCCCCTAACTCCGCCCATCCCGCCCCTAACTCCGCCCAGTTCCGCCCATTCTCCGCCCCATGGCTGACTAATTTTTTTTATTTATGCAGAGGCCGAGGCCGCCTCTGCCTCTGAGCTATTCCAGAAGTAGTGAGGAGGCTTTTTTGGAGGCCTAGGCTTTTGCAAAAAGCTCCCGGGAGCTTGTATATCCATTTTCGGATCTGATCAAGAGACAGGATGAGGATCGTTTCGCATGATTGAACAAGATGGATTGCACGCAGGTTCTCCGGCCGCTTGGGTGGAGAGGCTATTCGGCTATGACTGGGCACAACAGACAATCGGCTGCTCTGATGCCGCCGTGTTCCGGCTGTCAGCGCAGGGGCGCCCGGTTCTTTTTGTCAAGACCGACCTGTCCGGTGCCCTGAATGAACTGCAGGACGAGGCAGCGCGGCTATCGTGGCTGGCCACGACGGGCGTTCCTTGCGCAGCTGTGCTCGACGTTGTCACTGAAGCGGGAAGGGACTGGCTGCTATTGGGCGAAGTGCCGGGGCAGGATCTCCTGTCATCTCACCTTGCTCCTGCCGAGAAAGTATCCATCATGGCTGATGCAATGCGGCGGCTGCATACGCTTGATCCGGCTACCTGCCCATTCGACCACCAAGCGAAACATCGCATCGAGCGAGCACGTACTCGGATGGAAGCCGGTCTTGTCGATCAGGATGATCTGGACGAAGAGCATCAGGGGCTCGCGCCAGCCGAACTGTTCGCCAGGCTCAAGGCGCGCATGCCCGACGGCGAGGATCTCGTCGTGACCCATGGCGATGCCTGCTTGCCGAATATCATGGTGGAAAATGGCCGCTTTTCTGGATTCATCGACTGTGGCCGGCTGGGTGTGGCGGACCGCTATCAGGACATAGCGTTGGCTACCCGTGATATTGCTGAAGAGCTTGGCGGCGAATGGGCTGACCGCTTCCTCGTGCTTTACGGTATCGCCGCTCCCGATTCGCAGCGCATCGCCTTCTATCGCCTTCTTGACGAGTTCTTCTGAGCGGGACTCTGGGGTTCGAAATGACCGACCAAGCGACGCCCAACCTGCCATCACGAGATTTCGATTCCACCGCCGCCTTCTATGAAAGGTTGGGCTTCGGAATCGTTTTCCGGGACGCCGGCTGGATGATCCTCCAGCGCGGGGATCTCATGCTGGAGTTCTTCGCCCACCCCAACTTGTTTATTGCAGCTTATAATGGTTACAAATAAAGCAATAGCATCACAAATTTCACAAATAAAGCATTTTTTTCACTGCATTCTAGTTGTGGTTTGTCCAAACTCATCAATGTATCTTATCATGTCTGTATACCGTCGACCTCTAGCTAGAGCTTGGCGTAATCATGGTCATAGCTGTTTCCTGTGTGAAATTGTTATCCGCTCACAATTCCACACAACATACGAGCCGGAAGCATAAAGTGTAAAGCCTGGGGTGCCTAATGAGTGAGCTAACTCACATTAATTGCGTTGCGCTCACTGCCCGCTTTCCAGTCGGGAAACCTGTCGTGCCAGCTGCATTAATGAATCGGCCAACGCGCGGGGAGAGGCGGTTTGCGTATTGGGCGCTCTTCCGCTTCCTCGCTCACTGACTCGCTGCGCTCGGTCGTTCGGCTGCGGCGAGCGGTATCAGCTCACTCAAAGGCGGTAATACGGTTATCCACAGAATCAGGGGATAACGCAGGAAAGAACATGTGAGCAAAAGGCCAGCAAAAGGCCAGGAACCGTAAAAAGGCCGCGTTGCTGGCGTTTTTCCATAGGCTCCGCCCCCCTGACGAGCATCACAAAAATCGACGCTCAAGTCAGAGGTGGCGAAACCCGACAGGACTATAAAGATACCAGGCGTTTCCCCCTGGAAGCTCCCTCGTGCGCTCTCCTGTTCCGACCCTGCCGCTTACCGGATACCTGTCCGCCTTTCTCCCTTCGGGAAGCGTGGCGCTTTCTCATAGCTCACGCTGTAGGTATCTCAGTTCGGTGTAGGTCGTTCGCTCCAAGCTGGGCTGTGTGCACGAACCCCCCGTTCAGCCCGACCGCTGCGCCTTATCCGGTAACTATCGTCTTGAGTCCAACCCGGTAAGACACGACTTATCGCCACTGGCAGCAGCCACTGGTAACAGGATTAGCAGAGCGAGGTATGTAGGCGGTGCTACAGAGTTCTTGAAGTGGTGGCCTAACTACGGCTACACTAGAAGAACAGTATTTGGTATCTGCGCTCTGCTGAAGCCAGTTACCTTCGGAAAAAGAGTTGGTAGCTCTTGATCCGGCAAACAAACCACCGCTGGTAGCGGTGGTTTTTTTGTTTGCAAGCAGCAGATTACGCGCAGAAAAAAAGGATCTCAAGAAGATCCTTTGATCTTTTCTACGGGGTCTGACGCTCAGTGGAACGAAAACTCACGTTAAGGGATTTTGGTCATGAGATTATCAAAAAGGATCTTCACCTAGATCCTTTTAAATTAAAAATGAAGTTTTAAATCAATCTAAAGTATATATGAGTAAACTTGGTCTGACAGTTACCAATGCTTAATCAGTGAGGCACCTATCTCAGCGATCTGTCTATTTCGTTCATCCATAGTTGCCTGACTCCCCGTCGTGTAGATAACTACGATACGGGAGGGCTTACCATCTGGCCCCAGTGCTGCAATGATACCGCGAGACCCACGCTCACCGGCTCCAGATTTATCAGCAATAAACCAGCCAGCCGGAAGGGCCGAGCGCAGAAGTGGTCCTGCAACTTTATCCGCCTCCATCCAGTCTATTAATTGTTGCCGGGAAGCTAGAGTAAGTAGTTCGCCAGTTAATAGTTTGCGCAACGTTGTTGCCATTGCTACAGGCATCGTGGTGTCACGCTCGTCGTTTGGTATGGCTTCATTCAGCTCCGGTTCCCAACGATCAAGGCGAGTTACATGATCCCCCATGTTGTGCAAAAAAGCGGTTAGCTCCTTCGGTCCTCCGATCGTTGTCAGAAGTAAGTTGGCCGCAGTGTTATCACTCATGGTTATGGCAGCACTGCATAATTCTCTTACTGTCATGCCATCCGTAAGATGCTTTTCTGTGACTGGTGAGTACTCAACCAAGTCATTCTGAGAATAGTGTATGCGGCGACCGAGTTGCTCTTGCCCGGCGTCAATACGGGATAATACCGCGCCACATAGCAGAACTTTAAAAGTGCTCATCATTGGAAAACGTTCTTCGGGGCGAAAACTCTCAAGGATCTTACCGCTGTTGAGATCCAGTTCGATGTAACCCACTCGTGCACCCAACTGATCTTCAGCATCTTTTACTTTCACCAGCGTTTCTGGGTGAGCAAAAACAGGAAGGCAAAATGCCGCAAAAAAGGGAATAAGGGCGACACGGAAATGTTGAATACTCATACTCTTCCTTTTTCAATATTATTGAAGCATTTATCAGGGTTATTGTCTCATGAGCGGATACATATTTGAATGTATTTAGAAAAATAAACAAATAGGGGTTCCGCGCACATTTCCCCGAAAAGTGCCACCTGACGTC

## aa sequence of recombinant eVNN3 with hexahistidine–Twin-Strep tag

Translation 554 aa, MW= 62369.543Da:

MITSYFPKCVAVFALSVLSVGALDTFIAAVYEHAVILPNGTQTPVPKEEALLLMNKNIDVLEKAVKLAARQGAHIIVTPEDGIYGWVFTRETIYPYLEDIPHPEVNWIPCRDPQRFGHTPVQERLSCMAKDNSIYVVANIGDKKPCNASDPECPPDGRYQYNTDVVFDSEGRLVARYHKYNLFAPEIQFDFPKDSEFVTFDTPFGKFGIFTCFDIFSHDPAVVVVNEFQVDSILYPTAWYNTLPLLSAVPFHSAWARAMRVNLLAANTHNTSMHMTGSGIYAPEAVKVYYYDMETESGQLMLSELKSRPRRETTYPAAVDWSAYARGVKPFPSEQPDFPGMIYFDEFTFTELQKNTGNYTVCQKDLCCHLTYQMSEKRTDEVYALGAFDGLHTVEGQYYLQICTLLKCQTTELRTCGEPAGSAFTRFEEFSLSGTFVTSYVFPQLILSGSQLAPERHYEVSQDGRLRSRGGAPLPVLVMALYGRVFEKDPPRLGQGPEKLQRPPSLAPFQDSRENLYFQGHHHHHHWSHPQFEKGGGSGGGSGGSAWSHPQFEK

## VNN1(X4) clone XM_023651108.2 pDSG102

GTTGCCAGCCATCTGTTGTTTGCCCCTCCCCCGTGCCTTCCTTGACCCTGGAAGGTGCCACTCCCACTGTCCTTTCCTAATAAAATGAGGAAATTGCATCGCATTGTCTGAGTAGGTGTCATTCTATTCTGGGGGGTGGGGTGGGGCAGGACAGCAAGGGGGAGGATTGGGAAGACAATAGCAGGCATGCTGGGGATGCGGTGGGCTCTATGGAGCTTGGCCGCGTTGCTGGCGTTTTTCCATAGGCTCCGCCCCCCTGACGAGCATCACAAAAATCGACGCTCAAGTCAGAGGTGGCGAAACCCGACAGGACTATAAAGATACCAGGCGTTTCCCCCTGGAAGCTCCCTCGTGCGCTCTCCTGTTCCGACCCTGCCGCTTACCGGATACCTGTCCGCCTTTCTCCCTTCGGGAAGCGTGGCGCTTTCTCATAGCTCACGCTGTAGGTATCTCAGTTCGGTGTAGGTCGTTCGCTCCAAGCTGGGCTGTGTGCACGAACCCCCCGTTCAGCCCGACCGCTGCGCCTTATCCGGTAACTATCGTCTTGAGTCCAACCCGGTAAGACACGACTTATCGCCACTGGCAGCAGCCACTGGTAACAGGATTAGCAGAGCGAGGTATGTAGGCGGTGCTACAGAGTTCTTGAAGTGGTGGCCTAACTACGGCTACACTAGAAGAACAGTATTTGGTATCTGCGCTCTGCTGAAGCCAGTTACCTTCGGAAAAAGAGTTGGTAGCTCTTGATCCGGCAAACAAACCACCGCTGGTAGCGGTGGTTTTTTTGTTTGCAAGCAGCAGATTACGCGCAGAAAAAAAGGATCTCAAGAAGATCCTTTGATCTTTTCTACGGGGTCTGACGCTCAGTGGAACGAAAACTCACGTTAAGGGATTTTGGTCATGAGATTATCAAAAAGGATCTTCACCTAGATCCTTTTAAATTAAAAATGAAGTTTTAAATCAATCTAAAGTATATATGAGTAAACTTGGTCTGACAGTTACCAATGCTTAATCAGTGAGGCACCTATCTCAGCGATCTGTCTATTTCGTTCATCCATAGTTGCCTGACTCCCCGTCGTGTAGATAACTACGATACGGGAGGGCTTACCATCTGGCCCCAGTGCTGCAATGATACCGCGAGACCCACGCTCACCGGCTCCAGATTTATCAGCAATAAACCAGCCAGCCGGAAGGGCCGAGCGCAGAAGTGGTCCTGCAACTTTATCCGCCTCCATCCAGTCTATTAATTGTTGCCGGGAAGCTAGAGTAAGTAGTTCGCCAGTTAATAGTTTGCGCAACGTTGTTGCCATTGCTACAGGCATCGTGGTGTCACGCTCGTCGTTTGGTATGGCTTCATTCAGCTCCGGTTCCCAACGATCAAGGCGAGTTACATGATCCCCCATGTTGTGCAAAAAAGCGGTTAGCTCCTTCGGTCCTCCGATCGTTGTCAGAAGTAAGTTGGCCGCAGTGTTATCACTCATGGTTATGGCAGCACTGCATAATTCTCTTACTGTCATGCCATCCGTAAGATGCTTTTCTGTGACTGGTGAGTACTCAACCAAGTCATTCTGAGAATAGTGTATGCGGCGACCGAGTTGCTCTTGCCCGGCGTCAATACGGGATAATACCGCGCCACATAGCAGAACTTTAAAAGTGCTCATCATTGGAAAACGTTCTTCGGGGCGAAAACTCTCAAGGATCTTACCGCTGTTGAGATCCAGTTCGATGTAACCCACTCGTGCACCCAACTGATCTTCAGCATCTTTTACTTTCACCAGCGTTTCTGGGTGAGCAAAAACAGGAAGGCAAAATGCCGCAAAAAAGGGAATAAGGGCGACACGGAAATGTTGAATACTCATACTCTTCCTTTTTCAATATTATTGAAGCATTTATCAGGGTTATTGTCTCATGAGCGGATACATATTTGAATGTATTTAGAAAAATAAACAAATAGGGGTTCCGCGCACATTTCCCCGAAAAGTGCCACCTGACGTCTAGGTTCACCTAAGAATGGGAGCAACCAGCAGGAAAAGGACAAGCAGCGAAAATTCACGCCCCCTTGGGAGGTGGCGGCATATGCAAAGGATAGCACTCCCACTCTACTACTGGGTATCATATGCTGACTGTATATGCATGAGGATAGCATATGCTACCCGGATACAGATTAGGATAGCATATACTACCCAGATATAGATTAGGATAGCATATGCTACCCAGATATAGATTAGGATAGCCTATGCTACCCAGATATAAATTAGGATAGCATATACTACCCAGATATAGATTAGGATAGCATATGCTACCCAGATATAGATTAGGATAGCCTATGCTACCCAGATATAGATTAGGATAGCATATGCTACCCAGATATAGATTAGGATAGCATATGCTATCCAGATATTTGGGTAGTATATGCTACCCAGATATAAATTAGGATAGCATATACTACCCTAATCTCTATTAGGATAGCATATGCTACCCGGATACAGATTAGGATAGCATATACTACCCAGATATAGATTAGGATAGCATATGCTACCCAGATATAGATTAGGATAGCCTATGCTACCCAGATATAAATTAGGATAGCATATACTACCCAGATATAGATTAGGATAGCATATGCTACCCAGATATAGATTAGGATAGCCTATGCTACCCAGATATAGATTAGGATAGCATATGCTATCCAGATATTTGGGTAGTATATGCTACCCATGGCAACATTAGCCCACCGTGCTCTCAGCGACCTCGTGAATATGAGGACCAACAACCCTGTGCTTGGCGCTCAGGCGCAAGTGTGTGTAATTTGTCCTCCAGATCGCAGCAATCGCGCCCCTATCTTGGCCCGCCCACCTACTTATGCAGGTATTCCCCGGGGTGCCATTAGTGGTTTTGTGGGCAAGTGGTTTGACCGCAGTGGTTAGCGGGGTTACAATCAGCCAAGTTATTACACCCTTATTTTACAGTCCAAAACCGCAGGGCGGCGTGTGGGGGCTGACGCGTGCCCCCACTCCACAATTTCAAAAAAAAGAGTGGCCACTTGTCTTTGTTTATGGGCCCCATTGGCGTGGAGCCCCGTTTAATTTTCGGGGGTGTTAGAGACAACCAGTGGAGTCCGCTGCTGTCGGCGTCCACTCTCTTTCCCCTTGTTACAAATAGAGTGTAACAACATGGTTCACCTGTCTTGGTCCCTGCCTGGGACACATCTTAATAACCCCAGTATCATATTGCACTAGGATTATGTGTTGCCCATAGCCATAAATTCGTGTGAGATGGACATCCAGTCTTTACGGCTTGTCCCCACCCCATGGATTTCTATTGTTAAAGATATTCAGAATGTTTCATTCCTACACTAGTATTTATTGCCCAAGGGGTTTGTGAGGGTTATATTGGTGTCATAGCACAATGCCACCACTGAACCCCCCGTCCAAATTTTATTCTGGGGGCGTCACCTGAAACCTTGTTTTCGAGCACCTCACATACACCTTACTGTTCACAACTCAGCAGTTATTCTATTAGCTAAACGAAGGAGAATGAAGAAGCAGGCGAAGATTCAGGAGAGTTCACTGCCCGCTCCTTGATCTTCAGCCACTGCCCTTGTGACTAAAATGGTTCACTACCCTCGTGGAATCCTGACCCCATGTAAATAAAACCGTGACAGCTCATGGGGTGGGAGATATCGCTGTTCCTTAGGACCCTTTTACTAACCCTAATTCGATAGCATATGCTTCCCGTTGGGTAACATATGCTATTGAATTAGGGTTAGTCTGGATAGTATATACTACTACCCGGGAAGCATATGCTACCCGTTTAGGGTTAACAAGGGGGCCTTATAAACACTATTGCTAATGCCCTCTTGAGGGTCCGCTTATCGGTAGCTACACAGGCCCCTCTGATTGACGTTGGTGTAGCCTCCCGTAGTCTTCCTGGGCCCCTGGGAGGTACATGTCCCCCAGCATTGGTGTAAGAGCTTCAGCCAAGAGTTACACATAAAGGCAATGTTGTGTTGCAGTCCACAGACTGCAAAGTCTGCTCCAGGATGAAAGCCACTCAGTGTTGGCAAATGTGCACATCCATTTATAAGGATGTCAACTACAGTCAGAGAACCCCTTTGTGTTTGGTCCCCCCCCGTGTCACATGTGGAACAGGGCCCAGTTGGCAAGTTGTACCAACCAACTGAAGGGATTACATGCACTGCCCCGCATTAATTGCATGAAGAATCTGCTTAGGGTTAGGCGTTTTGCGCTGCTTCGCGATGTACGGGCCAGATATACGCGTTGACATTGATTATTGACTAGTTATTAATAGTAATCAATTACGGGGTCATTAGTTCATAGCCCATATATGGAGTTCCGCGTTACATAACTTACGGTAAATGGCCCGCCTGGCTGACCGCCCAACGACCCCCGCCCATTGACGTCAATAATGACGTATGTTCCCATAGTAACGCCAATAGGGACTTTCCATTGACGTCAATGGGTGGAGTATTTACGGTAAACTGCCCACTTGGCAGTACATCAAGTGTATCATATGCCAAGTACGCCCCCTATTGACGTCAATGACGGTAAATGGCCCGCCTGGCATTATGCCCAGTACATGACCTTATGGGACTTTCCTACTTGGCAGTACATCTACGTATTAGTCATCGCTATTACCATGGTGATGCGGTTTTGGCAGTACATCAATGGGCGTGGATAGCGGTTTGACTCACGGGGATTTCCAAGTCTCCACCCCATTGACGTCAATGGGAGTTTGTTTTGGCACCAAAATCAACGGGACTTTCCAAAATGTCGTAACAACTCCGCCCCATTGACGCAAATGGGCGGTAGGCGTGTACGGTGGGAGGTCTATATAAGCAGAGCTCTCTGGCTAACTAGAGAACCCACTGCTTACTGGCTTATCGAAATTAATACGACTCACTATAGGGTCTAGACCCACAATGAGGGCCTGGATCTTCTTTCTCCTTTGCCTGGCCGGGAGGGCTCTGGCAGCAATGCTGGAGACCTTTATTGCAGCTGTTTACGAGCACGCAGTGATATTGCCCAATGCCACCCTAACGCCAGTGTCCCGGGAAGAGGCGTTGCCATTAATGAACCGCAATCTGGATCTTTTGGAGGGAGCAATCACCTCAGCAGCCAAGCAGGGTGCGCACATCATTGTGACTCCAGAAGATGGTGTTTACGGCTGGAACTTCAGCAGGGACACGCTCTATCCGTACTTGGAGGACATCCCAGACCCTGCAGTGAACTGGATCCCTTGCAACAATCCCGACAGATTTGGCCACACTCCAGTCCAAGAGAGGCTCAGCTGCCTGGCCAAGGACAACTCTATCTACGTCGTGGCAAATATCGGGGACAAGAAGCCATGCAATGCCAGTGACCCTGAGTGCCCCCCTGATGGCCGTTACCAGTACAACACTGATGTGGTGTTTGATGCTCAGGGAAAACTTGTGGCACGCTACCATAAGCAAAACCTTTTCATGGGTGAAGATCAATTCAATGCACCCAAGGAGCCTGAGGTCGTGACTTTCAACACCACCTTTGGAAAGTTTGGCATTTTCACGTGCTTCGATATTCTCTTCTACGACCCTGCTGTTACCCTGGTGACAGATTTCCAAGTGGACACCATCCTCTTCCCGACAGCCTGGATGAATGTTTTGCCACATTTGTCAGCTATTGAATTTCACTCGGCTTGGGCTATGGGCATGGGGGTCAATTTCCTTGCATCCAATATACATTACCCCTCAATGAAAATGACAGGAAGTGGCATCTATGCTCCTGATTCTCCAAGAGCATTTCATTATGATATGAAGACGGAGGAGGGAAAACTACTCCTCTCACAGCTGGACTCCCACCCGCACCACCCAGCAGTGGTGAACTGGACTTCTTTTGCCAGTGGTGTGAAAACACTCTCAACGGGAAACCAGGAATTTAACAGCACTGTCTTTTTTGATGAATTCACCTTCCTGGAGCTCACCGAAGTCACAGGAAATTACACAGTTTGTCAGAAAGCTCTCTGCTGTCACCTAAGCTACAAGATGTCTGAGAAGAGATCAGATGAAGTGTACGCCCTAGGGGCATTTGATGGCTTACATACTGTTGAAGGGAGTTATTATTTACAGGTTTGCACTCTGTTGAAGTGTAGAACCACGGACTTACACACTTGTGGTGACTCGGTGGAAACTGCTTCCACTAGGTTCGATACGTTCTCCCTCAGTGGCACTTTTGGGACCCAGCATGTCTTCCCTGAGGTGCTGCTGAGTGAAATTCAGCTCGCACCTGGAGAATTCCAGGTGTCAAGTGATGGACGCTTGTTCAGTCTGCAGCCCCCCTCGGGACCTGTCCTGACAGTGACTCTTTATGGGAGGCTGTACGAGAAGGACCAGGTATCCGACGCCTCATCAGACCTCACAGCACGAGCACTGAGAGACAGATTAGGAGATGCTGAGAACCTGTACTTCCAAGGCCACCACCACCATCACCACGGGAGCGCTTGGAGCCACCCGCAGTTCGAAAAAGGTGGAGGTTCTGGCGGTGGATCGGGAGGTTCAGCGTGGAGCCACCCGCAGTTCGAGAAATAAGGGAGCCACCCGCAAGCTTGCGGCCGCAGATCTAGCTTAAGTTTAAACCGCTGATCAGCCTCGACTGTGCCTTCTA

## aa sequence of recombinant eVNN1 with hexahistidine–Twin-Strep tag

Translation 546 aa, MW=60545.9 Da:

MRAWIFFLLCLAGRALAAMLETFIAAVYEHAVILPNATLTPVSREEALPLMNRNLDLLEGAITSAAKQGAHIIVTPEDGVYGWNFSRDTLYPYLEDIPDPAVNWIPCNNPDRFGHTPVQERLSCLAKDNSIYVVANIGDKKPCNASDPECPPDGRYQYNTDVVFDAQGKLVARYHKQNLFMGEDQFNAPKEPEVVTFNTTFGKFGIFTCFDILFYDPAVTLVTDFQVDTILFPTAWMNVLPHLSAIEFHSAWAMGMGVNFLASNIHYPSMKMTGSGIYAPDSPRAFHYDMKTEEGKLLLSQLDSHPHHPAVVNWTSFASGVKTLSTGNQEFNSTVFFDEFTFLELTEVTGNYTVCQKALCCHLSYKMSEKRSDEVYALGAFDGLHTVEGSYYLQVCTLLKCRTTDLHTCGDSVETASTRFDTFSLSGTFGTQHVFPEVLLSEIQLAPGEFQVSSDGRLFSLQPPSGPVLTVTLYGRLYEKDQVSDASSDLTARALRDRLGDAENLYFQGHHHHHHGSAWSHPQFEKGGGSGGGSGGSAWSHPQFEK

## eCLCA1 clone pDSG102

GTTGCCAGCCATCTGTTGTTTGCCCCTCCCCCGTGCCTTCCTTGACCCTGGAAGGTGCCACTCCCACTGTCCTTTCCTAATAAAATGAGGAAATTGCATCGCATTGTCTGAGTAGGTGTCATTCTATTCTGGGGGGTGGGGTGGGGCAGGACAGCAAGGGGGAGGATTGGGAAGACAATAGCAGGCATGCTGGGGATGCGGTGGGCTCTATGGAGCTTGGCCGCGTTGCTGGCGTTTTTCCATAGGCTCCGCCCCCCTGACGAGCATCACAAAAATCGACGCTCAAGTCAGAGGTGGCGAAACCCGACAGGACTATAAAGATACCAGGCGTTTCCCCCTGGAAGCTCCCTCGTGCGCTCTCCTGTTCCGACCCTGCCGCTTACCGGATACCTGTCCGCCTTTCTCCCTTCGGGAAGCGTGGCGCTTTCTCATAGCTCACGCTGTAGGTATCTCAGTTCGGTGTAGGTCGTTCGCTCCAAGCTGGGCTGTGTGCACGAACCCCCCGTTCAGCCCGACCGCTGCGCCTTATCCGGTAACTATCGTCTTGAGTCCAACCCGGTAAGACACGACTTATCGCCACTGGCAGCAGCCACTGGTAACAGGATTAGCAGAGCGAGGTATGTAGGCGGTGCTACAGAGTTCTTGAAGTGGTGGCCTAACTACGGCTACACTAGAAGAACAGTATTTGGTATCTGCGCTCTGCTGAAGCCAGTTACCTTCGGAAAAAGAGTTGGTAGCTCTTGATCCGGCAAACAAACCACCGCTGGTAGCGGTGGTTTTTTTGTTTGCAAGCAGCAGATTACGCGCAGAAAAAAAGGATCTCAAGAAGATCCTTTGATCTTTTCTACGGGGTCTGACGCTCAGTGGAACGAAAACTCACGTTAAGGGATTTTGGTCATGAGATTATCAAAAAGGATCTTCACCTAGATCCTTTTAAATTAAAAATGAAGTTTTAAATCAATCTAAAGTATATATGAGTAAACTTGGTCTGACAGTTACCAATGCTTAATCAGTGAGGCACCTATCTCAGCGATCTGTCTATTTCGTTCATCCATAGTTGCCTGACTCCCCGTCGTGTAGATAACTACGATACGGGAGGGCTTACCATCTGGCCCCAGTGCTGCAATGATACCGCGAGACCCACGCTCACCGGCTCCAGATTTATCAGCAATAAACCAGCCAGCCGGAAGGGCCGAGCGCAGAAGTGGTCCTGCAACTTTATCCGCCTCCATCCAGTCTATTAATTGTTGCCGGGAAGCTAGAGTAAGTAGTTCGCCAGTTAATAGTTTGCGCAACGTTGTTGCCATTGCTACAGGCATCGTGGTGTCACGCTCGTCGTTTGGTATGGCTTCATTCAGCTCCGGTTCCCAACGATCAAGGCGAGTTACATGATCCCCCATGTTGTGCAAAAAAGCGGTTAGCTCCTTCGGTCCTCCGATCGTTGTCAGAAGTAAGTTGGCCGCAGTGTTATCACTCATGGTTATGGCAGCACTGCATAATTCTCTTACTGTCATGCCATCCGTAAGATGCTTTTCTGTGACTGGTGAGTACTCAACCAAGTCATTCTGAGAATAGTGTATGCGGCGACCGAGTTGCTCTTGCCCGGCGTCAATACGGGATAATACCGCGCCACATAGCAGAACTTTAAAAGTGCTCATCATTGGAAAACGTTCTTCGGGGCGAAAACTCTCAAGGATCTTACCGCTGTTGAGATCCAGTTCGATGTAACCCACTCGTGCACCCAACTGATCTTCAGCATCTTTTACTTTCACCAGCGTTTCTGGGTGAGCAAAAACAGGAAGGCAAAATGCCGCAAAAAAGGGAATAAGGGCGACACGGAAATGTTGAATACTCATACTCTTCCTTTTTCAATATTATTGAAGCATTTATCAGGGTTATTGTCTCATGAGCGGATACATATTTGAATGTATTTAGAAAAATAAACAAATAGGGGTTCCGCGCACATTTCCCCGAAAAGTGCCACCTGACGTCTAGGTTCACCTAAGAATGGGAGCAACCAGCAGGAAAAGGACAAGCAGCGAAAATTCACGCCCCCTTGGGAGGTGGCGGCATATGCAAAGGATAGCACTCCCACTCTACTACTGGGTATCATATGCTGACTGTATATGCATGAGGATAGCATATGCTACCCGGATACAGATTAGGATAGCATATACTACCCAGATATAGATTAGGATAGCATATGCTACCCAGATATAGATTAGGATAGCCTATGCTACCCAGATATAAATTAGGATAGCATATACTACCCAGATATAGATTAGGATAGCATATGCTACCCAGATATAGATTAGGATAGCCTATGCTACCCAGATATAGATTAGGATAGCATATGCTACCCAGATATAGATTAGGATAGCATATGCTATCCAGATATTTGGGTAGTATATGCTACCCAGATATAAATTAGGATAGCATATACTACCCTAATCTCTATTAGGATAGCATATGCTACCCGGATACAGATTAGGATAGCATATACTACCCAGATATAGATTAGGATAGCATATGCTACCCAGATATAGATTAGGATAGCCTATGCTACCCAGATATAAATTAGGATAGCATATACTACCCAGATATAGATTAGGATAGCATATGCTACCCAGATATAGATTAGGATAGCCTATGCTACCCAGATATAGATTAGGATAGCATATGCTATCCAGATATTTGGGTAGTATATGCTACCCATGGCAACATTAGCCCACCGTGCTCTCAGCGACCTCGTGAATATGAGGACCAACAACCCTGTGCTTGGCGCTCAGGCGCAAGTGTGTGTAATTTGTCCTCCAGATCGCAGCAATCGCGCCCCTATCTTGGCCCGCCCACCTACTTATGCAGGTATTCCCCGGGGTGCCATTAGTGGTTTTGTGGGCAAGTGGTTTGACCGCAGTGGTTAGCGGGGTTACAATCAGCCAAGTTATTACACCCTTATTTTACAGTCCAAAACCGCAGGGCGGCGTGTGGGGGCTGACGCGTGCCCCCACTCCACAATTTCAAAAAAAAGAGTGGCCACTTGTCTTTGTTTATGGGCCCCATTGGCGTGGAGCCCCGTTTAATTTTCGGGGGTGTTAGAGACAACCAGTGGAGTCCGCTGCTGTCGGCGTCCACTCTCTTTCCCCTTGTTACAAATAGAGTGTAACAACATGGTTCACCTGTCTTGGTCCCTGCCTGGGACACATCTTAATAACCCCAGTATCATATTGCACTAGGATTATGTGTTGCCCATAGCCATAAATTCGTGTGAGATGGACATCCAGTCTTTACGGCTTGTCCCCACCCCATGGATTTCTATTGTTAAAGATATTCAGAATGTTTCATTCCTACACTAGTATTTATTGCCCAAGGGGTTTGTGAGGGTTATATTGGTGTCATAGCACAATGCCACCACTGAACCCCCCGTCCAAATTTTATTCTGGGGGCGTCACCTGAAACCTTGTTTTCGAGCACCTCACATACACCTTACTGTTCACAACTCAGCAGTTATTCTATTAGCTAAACGAAGGAGAATGAAGAAGCAGGCGAAGATTCAGGAGAGTTCACTGCCCGCTCCTTGATCTTCAGCCACTGCCCTTGTGACTAAAATGGTTCACTACCCTCGTGGAATCCTGACCCCATGTAAATAAAACCGTGACAGCTCATGGGGTGGGAGATATCGCTGTTCCTTAGGACCCTTTTACTAACCCTAATTCGATAGCATATGCTTCCCGTTGGGTAACATATGCTATTGAATTAGGGTTAGTCTGGATAGTATATACTACTACCCGGGAAGCATATGCTACCCGTTTAGGGTTAACAAGGGGGCCTTATAAACACTATTGCTAATGCCCTCTTGAGGGTCCGCTTATCGGTAGCTACACAGGCCCCTCTGATTGACGTTGGTGTAGCCTCCCGTAGTCTTCCTGGGCCCCTGGGAGGTACATGTCCCCCAGCATTGGTGTAAGAGCTTCAGCCAAGAGTTACACATAAAGGCAATGTTGTGTTGCAGTCCACAGACTGCAAAGTCTGCTCCAGGATGAAAGCCACTCAGTGTTGGCAAATGTGCACATCCATTTATAAGGATGTCAACTACAGTCAGAGAACCCCTTTGTGTTTGGTCCCCCCCCGTGTCACATGTGGAACAGGGCCCAGTTGGCAAGTTGTACCAACCAACTGAAGGGATTACATGCACTGCCCCGCATTAATTGCATGAAGAATCTGCTTAGGGTTAGGCGTTTTGCGCTGCTTCGCGATGTACGGGCCAGATATACGCGTTGACATTGATTATTGACTAGTTATTAATAGTAATCAATTACGGGGTCATTAGTTCATAGCCCATATATGGAGTTCCGCGTTACATAACTTACGGTAAATGGCCCGCCTGGCTGACCGCCCAACGACCCCCGCCCATTGACGTCAATAATGACGTATGTTCCCATAGTAACGCCAATAGGGACTTTCCATTGACGTCAATGGGTGGAGTATTTACGGTAAACTGCCCACTTGGCAGTACATCAAGTGTATCATATGCCAAGTACGCCCCCTATTGACGTCAATGACGGTAAATGGCCCGCCTGGCATTATGCCCAGTACATGACCTTATGGGACTTTCCTACTTGGCAGTACATCTACGTATTAGTCATCGCTATTACCATGGTGATGCGGTTTTGGCAGTACATCAATGGGCGTGGATAGCGGTTTGACTCACGGGGATTTCCAAGTCTCCACCCCATTGACGTCAATGGGAGTTTGTTTTGGCACCAAAATCAACGGGACTTTCCAAAATGTCGTAACAACTCCGCCCCATTGACGCAAATGGGCGGTAGGCGTGTACGGTGGGAGGTCTATATAAGCAGAGCTCTCTGGCTAACTAGAGAACCCACTGCTTACTGGCTTATCGAAATTAATACGACTCACTATAGGGTCTAGACCCACAATGAGGGCCTGGATCTTCTTTCTCCTTTGCCTGGCCGGGAGGGCTCTGGCAGCAATGAATTCACTCATTCATCTGAACAATAATGGTTATGAAGGCATTGTCATTGCAATTGACCCTAATGTGCCAGAAGATGAAACACTCATTCAACAAATAAAGGACATGGTGACCCAGGCATCTCCATATCTCTTTGAAGCTACAGAAAAAAGATTCTATTTCAAAAACGTTGCCATTTTGGTTCCTGAAAATTGGAAGACAAAACCTGAGTATGAGAGACCAAAACTCGAGACCTACAAAAATGCTGATGTTCTAGTTGCTGAACCTAATCCTCCAGGCAATGATCAACCCTACACTGAACAGATGGGAAAATGCGGAGAAAAGGGCGAAAGAATTTATTTCACTCCTGACTTCTTAGCAGGAAAAAGGTTGGATGAATATGGACCACAAGGAAGGGTATTTGTCCACGAATGGGCTCATCTACGATGGGGACTATTTAATGAGTACAATGATGACCAGAAATTCTACTTATCCAATAAAGAAATCAAACCAGTAAAATGTTCAGCAGATATTGCTGGTAAAAATGTGGTAAACCACTGTCAGGGAGGCAGCTGTGCCACCAAACCATGCAGACGCGACAGAGTAACAGGGCTGTATGCGCAAGAATGTGAGTTCATACCAGATGAACAGCAGACTGAGAAGGCTTCCATAATGTTTTCACAAAGTATTGATTCTGTGGTTGAATTCTGTACAGAAGAAAACCACAATAGAGAAGCCCCAAACATGCAAAACCAGAGATGCAATCTCCGAAGTACATGGGAAGTGATTCGTGATTCTGAGGACTTTAAGAAAACCACTCCTATGACGGCACAGCCACCCCAGCCCACTTTCTCACTGCTGCAGATTGGACAAAGAATTGTGTGTTTAGTCCTTGATAAATCTGGAAGCATGGCGATTGGTGACCGCCTTAAACGACTGACTCAAGCAGGCAAGCTTTTCCTGCTGCAGACGGTTGAGCAAGGGTCCTGGGTTGGAATGGTGACGTTTGACAGTGCTGCCTATGTACAAAGTGCACTCAGACAGATAAAAGGTGGCACTGATAGGGACGCGCTCACCAAAAGCTTACCCACAGTGGCCTCAGGAGGAACGTCCATCTGCTCCGGGCTTCGATCAGCCTTTACTGTAATTAGGAAGAAATACAAAACAGATGGATCTGAAATCGTGCTCTTGACTGATGGAGAGGACAACACTATAAGCTCATGCTTTAACGAGGTGAAGCAAAGTGGAGCCATCATCCACACAGTGGCCCTGGGGCCCTCTGCAGCAGCAGAACTGGAGGAGCTGTCTAAAATGACAGGAGGGTTACAGACATATGCTTCGGATCAGGCTCAGAACAATGGCCTCATTGATGCTTTCGGAGCCCTTTCGTCAGGAAACGGAGCTGTCTCTCAGCGCTCCATCCAGCTTCTGAGTAGGGGATCAACCCTCCAGAACAGTCAGTGGATGAACGGAACAGTGATTGTGGATAGCACTGTGGGAAATGACACTCTGTTTCTCATCACCTGGACATCACAACCTCCTCAAATCCTTCTCTGGGATCCCAGTGGAAAGAAACAAGATGGCTTTGTAGTAGACACAAATACCAAAATGGCCTACCTCCAGGTCCCAGGCACTGCCAAGGTTGGCACCTGGACTTACAGTCTGCAAGCAAGCTCCCAAACCCTGACTCTGACTGTCACCTCCCGTGCATCAAGTGCTACGTTGCCTCCAGTTACAGTGACCTCGAAAGTGAACAAAGACACAGGCAAATTCCCTAGCCCTGTGGTGGTTTATGCAAAGATTCACCAAGGAGGCTTACCAATTCTCAGGGCCACTGTCACAGCCCTGATAGAATCAGTGGATGGAAAAACAGTTACCTTGGAATTATTGGACAATGGAGCAGGTGCTGATGCTACTAAGGATGACGGTATCTATTCAAGGTATTTTACGGCTTACAATACAAATGGTAGATACAGTATAAAAGTGTGGGCTCTTGGAGGAGTGAACGCAGCCAGACAAATGGGGATATCCCAGCAGAATGGAGCCATGTACAGAGCTGGCAGGATGAAGAATGGTGAAATAATATGGAATCCACCGAGACCTAAAATCAATATCGATGACCTTCTGAGCAAGCAAGTGCCTTTCAGCAGAACATCCTCGGGAGGTTCATTTGTGGCCTCCAACGTCCCAAACGCTCCCATTCCTGATCTCTTCCCACCTTGTCAAATCACCGACCTGAAGGCAAAAATCCAAGGAGACAGTCTCATTAATCTGACTTGGACAGCTCCTGGGGACGATTACGATCATGGAAGGGCTGACAGGTACATCATTAGAATAAGTACAAATATTCTTGATCTCAGAGACAAGTTCAATGATTCGCTTCAAGTGAACACTACTGATCTCATCCCAAAGGAGGCCACCTCTGAGGAAGTCTTTGTCTTCAAACCAGAAAACATTGCTTTTGAAAATGGCACGGATCTTTTCATTGCTATTCAGGCTGTTGATGAGGTCGACCTGGAGTCAGAAATCTCCAACATTGCACAAGTATCATTGTTTCTTCCCCCACAGACTCCTCCAGAGACACCTAGTCCTTCTCTTCCTTGTCCTGATAGTAATATCACCAGCGCCATTCCTGGCATTCACATTTTAAAAGTTATGTGGAAGTGGCTAGGGGAATTGCAGCTGTCAGTAGCCTTGGGCGGGAGCGCTTGGAGCCACCCGCAGTTCGAAAAAGGTGGAGGTTCTGGCGGTGGATCGGGAGGTTCAGCGTGGAGCCACCCGCAGTTCGAGAAATAAGGGAGCCACCCGCAAGCTTGCGGCCGCAGATCTAGCTTAAGTTTAAACCGCTGATCAGCCTCGACTGTGCCTTCTA

## aa sequence of recombinant eCLCA with Twin-Strep tag

Translation 942 aa, MW=103160.63 Da.

MRAWIFFLLCLAGRALAAMNSLIHLNNNGYEGIVIAIDPNVPEDETLIQQIKDMVTQASPYLFEATEKRFYFKNVAILVPENWKTKPEYERPKLETYKNADVLVAEPNPPGNDQPYTEQMGKCGEKGERIYFTPDFLAGKRLDEYGPQGRVFVHEWAHLRWGLFNEYNDDQKFYLSNKEIKPVKCSADIAGKNVVNHCQGGSCATKPCRRDRVTGLYAQECEFIPDEQQTEKASIMFSQSIDSVVEFCTEENHNREAPNMQNQRCNLRSTWEVIRDSEDFKKTTPMTAQPPQPTFSLLQIGQRIVCLVLDKSGSMAIGDRLKRLTQAGKLFLLQTVEQGSWVGMVTFDSAAYVQSALRQIKGGTDRDALTKSLPTVASGGTSICSGLRSAFTVIRKKYKTDGSEIVLLTDGEDNTISSCFNEVKQSGAIIHTVALGPSAAAELEELSKMTGGLQTYASDQAQNNGLIDAFGALSSGNGAVSQRSIQLLSRGSTLQNSQWMNGTVIVDSTVGNDTLFLITWTSQPPQILLWDPSGKKQDGFVVDTNTKMAYLQVPGTAKVGTWTYSLQASSQTLTLTVTSRASSATLPPVTVTSKVNKDTGKFPSPVVVYAKIHQGGLPILRATVTALIESVDGKTVTLELLDNGAGADATKDDGIYSRYFTAYNTNGRYSIKVWALGGVNAARQMGISQQNGAMYRAGRMKNGEIIWNPPRPKINIDDLLSKQVPFSRTSSGGSFVASNVPNAPIPDLFPPCQITDLKAKIQGDSLINLTWTAPGDDYDHGRADRYIIRISTNILDLRDKFNDSLQVNTTDLIPKEATSEEVFVFKPENIAFENGTDLFIAIQAVDEVDLESEISNIAQVSLFLPPQTPPETPSPSLPCPDSNITSAIPGIHILKVMWKWLGELQLSVALGGSAWSHPQFEKGGGSGGGSGGSAWSHPQFEK

## SNPs in cloned eVNN1 sequence

Sequence Alignment of coding sequence of cloned eVNN1 with reference sequence. SNPs are marked in red letters and non-synonymous variant is highlighted yellow:

```
Reference/1-1515        ATGATTTCTTCTCAGCTGCTCGCTTATGTGGCAATTCTGGTTTTCTGCACCTTCCAAGCC	60
eVNN1_Clone/1-1512      ATGATTTCTTCTCAGCTGCTCGCTTATGTGGCAATTTTGGTTTTCTGCACCTTCCAAGCC	60
                        ************************************ ***********************

Reference/1-1515        CACTCCCTGGAGACCTTTATTGCAGCTGTTTACGAGCACGCAGTGATATTGCCCAATGCC	120
eVNN1_Clone/1-1512      CACTCCCTGGAGACCTTTATTGCAGCTGTTTACGAGCACGCAGTGATATTGCCCAATGCC	120
                        ************************************************************

Reference/1-1515        ACCCTAACGCCAGTGTCCCGGGAAGAGGCGTTGCCATTAATGAACCGCAATCTGGATCTT	180
eVNN1_Clone/1-1512      ACCCTAACGCCAGTGTCCCGGGAAGAGGCGTTGCCATTAATGAACCGCAATCTGGATCTT	180
                        ************************************************************

Reference/1-1515        TTGGAGGGAGCAATCACCTCAGCAGCCAAGCAGGGTGCGCACATCATCGTGACTCCAGAA	240
eVNN1_Clone/1-1512      TTGGAGGGAGCAATCACCTCAGCAGCCAAGCAGGGTGCGCACATCATTGTGACTCCAGAA	240
                        *********************************************** ************

Reference/1-1515        GATGGTGTTTACGGCTGGAACTTCAGCAGGGACACGCTCTATCCGTACTTGGAGGACATC	300
eVNN1_Clone/1-1512      GATGGTGTTTACGGCTGGAACTTCAGCAGGGACACGCTCTATCCGTACTTGGAGGACATC	300
                        ************************************************************

Reference/1-1515        CCAGACCCTGCAGTGAACTGGATCCCTTGCAACAATCCCGACAGATTTGGCCACACTCCA	360
eVNN1_Clone/1-1512      CCAGACCCTGCAGTGAACTGGATCCCTTGCAACAATCCCGACAGATTTGGCCACACTCCA	360
                        ************************************************************

Reference/1-1515        GTCCAAGAGAGGCTCAGCTGCCTGGCCAAGGACAACTCTATCTACGTCGTGGCAAATATC	420
eVNN1_Clone/1-1512      GTCCAAGAGAGGCTCAGCTGCCTGGCCAAGGACAACTCTATCTACGTCGTGGCAAATATC	420
                        ************************************************************

Reference/1-1515        GGGGACAAGAAGCCATGCAATGCCAGTGACCCTGAGTGCCCCCCTGATGGCCGTTACCAG	480
eVNN1_Clone/1-1512      GGGGACAAGAAGCCATGCAATGCCAGTGACCCTGAGTGCCCCCCTGATGGCCGTTACCAG	480
                        ************************************************************

Reference/1-1515        TACAACACTGATGTGGTGTTTGATGCTCAGGGAAAACTTGTGGCACGCTACCATAAGCAA	540
eVNN1_Clone/1-1512      TACAACACTGATGTGGTGTTTGATGCTCAGGGAAAACTTGTGGCACGCTACCATAAGCAA	540
                        ************************************************************

Reference/1-1515        AACCTTTTCATGGGTGAAGATCAATTCAATGCACCCAAGGAGCCTGAGGTCGTGACTTTC	600
eVNN1_Clone/1-1512      AACCTTTTCATGGGTGAAGATCAATTCAATGCACCCAAGGAGCCTGAGGTCGTGACTTTC	600
                        ************************************************************

Reference/1-1515        AACACCACCTTTGGAAAGTTTGGCATTTTCACGTGCTTCGATATTCTCTTCTACAACCCT	660
eVNN1_Clone/1-1512      AACACCACCTTTGGAAAGTTTGGCATTTTCACGTGCTTCGATATTCTCTTCTACGACCCT	660
                        ****************************************************** *****

Reference/1-1515        GCTGTTACCCTGGTGACAGATTTCCAAGTGGACACCATCCTCTTCCCGACAGCCTGGATG	720
eVNN1_Clone/1-1512      GCTGTTACCCTGGTGACAGATTTCCAAGTGGACACCATCCTCTTCCCGACAGCCTGGATG	720
                        ************************************************************

Reference/1-1515        AATGTTTTGCCACATTTGTCAGCTATTGAATTTCACTCGGCTTGGGCTATGGGCATGGGG	780
eVNN1_Clone/1-1512      AATGTTTTGCCACATTTGTCAGCTATTGAATTTCACTCGGCTTGGGCTATGGGCATGGGG	780
                        ************************************************************

Reference/1-1515        GTCAATTTCCTTGCATCCAATATACATTACCCCTCAATGAAAATGACAGGAAGTGGCATC	840
eVNN1_Clone/1-1512      GTCAATTTCCTTGCATCCAATATACATTACCCCTCAATGAAAATGACAGGAAGTGGCATC	840
                        ************************************************************
```

```
Reference/1-1515        TATGCTCCTGATTCTCCAAGAGCATTTCATTATGATATGAAGACGGAGGAGGGAAAACTA	900
eVNN1_Clone/1-1512      TATGCTCCTGATTCTCCAAGAGCATTTCATTATGATATGAAGACGGAGGAGGGAAAACTA	900
                        ************************************************************

Reference/1-1515        CTCCTCTCACAGCTGGACTCCCACCCGCACCACCCAGCAGTGGTGAACTGGACTTCTTTT	960
eVNN1_Clone/1-1512      CTCCTCTCACAGCTGGACTCCCACCCGCACCACCCAGCAGTGGTGAACTGGACTTCTTTT	960
                        ************************************************************

Reference/1-1515        GCCAGTGGTGTGAAAACACTCTCAACGGGAAACCAGGAATTTAACAGCACTGTCTTTTTT	1020
eVNN1_Clone/1-1512      GCCAGTGGTGTGAAAACACTCTCAACGGGAAACCAGGAATTTAACAGCACTGTCTTTTTT	1020
                        ************************************************************

Reference/1-1515        GATGAATTCACCTTCCTGGAGCTCACCGAAGTCACAGGAAATTACACAGTTTGTCAGAAA	1080
eVNN1_Clone/1-1512      GATGAATTCACCTTCCTGGAGCTCACCGAAGTCACAGGAAATTACACAGTTTGTCAGAAA	1080
                        ************************************************************

Reference/1-1515        GCTCTCTGCTGTCACCTAAGCTACAAGATGTCTGAGAAGAGATCAGATGAAGTGTACGCC	1140
eVNN1_Clone/1-1512      GCTCTCTGCTGTCACCTAAGCTACAAGATGTCTGAGAAGAGATCAGATGAAGTGTACGCC	1140
                        ************************************************************

Reference/1-1515        CTAGGGGCATTTGATGGCTTACATACTGTTGAAGGGAGTTATTATTTACAGGTTTGCACT	1200
eVNN1_Clone/1-1512      CTAGGGGCATTTGATGGCTTACATACTGTTGAAGGGAGTTATTATTTACAGGTTTGCACT	1200
                        ************************************************************

Reference/1-1515        CTGTTGAAGTGTAGAACCACGGACTTACACACTTGTGGTGACTCGGTGGAAACTGCTTCC	1260
eVNN1_Clone/1-1512      CTGTTGAAGTGTAGAACCACGGACTTACACACTTGTGGTGACTCGGTGGAAACTGCTTCC	1260
                        ************************************************************

Reference/1-1515        ACTAGGTTCGATACGTTCTCCCTCAGTGGCACTTTTGGGACCCAGCATGTCTTCCCTGAG	1320
eVNN1_Clone/1-1512      ACTAGGTTCGATACGTTCTCCCTCAGTGGCACTTTTGGGACCCAGCATGTCTTCCCTGAG	1320
                        ************************************************************

Reference/1-1515        GTGCTGCTGAGTGAAATTCAGCTCGCACCTGGAGAATTCCAGGTGTCAAGTGATGGACGC	1380
eVNN1_Clone/1-1512      GTGCTGCTGAGTGAAATTCAGCTCGCACCTGGAGAATTCCAGGTGTCAAGTGATGGACGC	1380
                        ************************************************************

Reference/1-1515        TTGTTCAGTCTGCAGCCCCCCTCGGGACCTGTCCTGACAGTGACTCTTTATGGGAGGCTG	1440
eVNN1_Clone/1-1512      TTGTTCAGTCTGCAGCCCCCCTCGGGACCTGTCCTGACAGTGACTCTTTATGGGAGGCTG	1440
                        ************************************************************

Reference/1-1515        TACGAGAAGGACCAGGTATCCGACGCCTCATCAGACCTCACAGCACGAGCACTGAGAGAC	1500
eVNN1_Clone/1-1512      TACGAGAAGGACCAGGTATCCGACGCCTCATCAGACCTCACAGCACGAGCACTGAGAGAC	1500
                        ************************************************************

Reference/1-1515        AGATTAGGAGATTAG	1515
eVNN1_Clone/1-1512      AGATTAGGAGAT---	1512
                        ************
```
