# Supplementary material for: Ortholog-specific Spatial Expression of Vanins in the Respiratory Tract of Horses
Source: J Histochem Cytochem. 2026 Aug 31:00221554261474565. Online ahead of print. doi: 10.1369/00221554261474565 (PMC13530036; doi:10.1369/00221554261474565)
Supplement: Supplementary file 4 [file sj-docx-4-jhc-10.1369_00221554261474565.docx]

QuPath parameters and script used for cellular expression analysis

Groovy script for color deconvolution, cell detection and pixel classification:

**// Set image type and perform color deconvolution**

setImageType('BRIGHTFIELD_OTHER')

setColorDeconvolutionStains('{"Name" : "H-DAB modified", "Stain 1" : "Hematoxylin", "Values 1" : "0.60509 0.6411 0.47207", "Stain 2" : "Probe Red", "Values 2" : "0.41096 0.73893 0.53395", "Background" : "255 255 255"}')

**// Cell detection for epithelium and glands**

runPlugin('qupath.imagej.detect.cells.WatershedCellDetection', '{"detectionImageBrightfield":"Hematoxylin OD","requestedPixelSizeMicrons":0.25,"backgroundRadiusMicrons":9.0,"backgroundByReconstruction":true,"medianRadiusMicrons":0.0,"sigmaMicrons":1.5,"minAreaMicrons":10.0,"maxAreaMicrons":200.0,"threshold":0.2,"maxBackground":2.0,"watershedPostProcess":true,"cellExpansionMicrons":6.0,"includeNuclei":true,"smoothBoundaries":true,"makeMeasurements":true}')

**// Cell detection for lungs**

runPlugin('qupath.imagej.detect.cells.WatershedCellDetection', '{"detectionImageBrightfield":"Hematoxylin OD","requestedPixelSizeMicrons":0.25,"backgroundRadiusMicrons":9.0,"backgroundByReconstruction":true,"medianRadiusMicrons":0.0,"sigmaMicrons":1.5,"minAreaMicrons":10.0,"maxAreaMicrons":200.0,"threshold":0.4,"maxBackground":2.0,"watershedPostProcess":true,"cellExpansionMicrons":2.0,"includeNuclei":true,"smoothBoundaries":true,"makeMeasurements":true}')

**// Add measurements from trained pixel classifier**

addPixelClassifierMeasurements("Vanin detector1", "Vanin detector1")
