# Supplementary material for: Ortholog-specific Spatial Expression of Vanins in the Respiratory Tract of Horses
Source: J Histochem Cytochem. 2026 Aug 31:00221554261474565. Online ahead of print. doi: 10.1369/00221554261474565 (PMC13530036; doi:10.1369/00221554261474565)
Supplement: Supplementary file 6 [file sj-docx-6-jhc-10.1369_00221554261474565.docx]

# Absolute Values Log10AUC LCMSMS

| Group | Substrate (Pantetheine) Concentration (µm) | Mean log_10_(AUC) Pantothenic Acid | Mean log_10_(AUC) Pantetheine |
| --- | --- | --- | --- |
| eVNN1 | 200 | 8.29 | 3.33 |
| eCLCA | 200 | 6.64 | 9.02 |
| No Enzyme | 200 | 6.61 | 9.03 |
| eVNN1 | 500 | 8.56 | 3.9 |
| eCLCA | 500 | 6.66 | 9.33 |
| No Enzyme | 500 | 6.84 | 9.23 |
| eVNN1 | 1000 | 8.98 | 8.26 |
| eCLCA | 1000 | 6.95 | 9.57 |
| No Enzyme | 1000 | 7.32 | 9.47 |
