# Supplementary material for: Ortholog-specific Spatial Expression of Vanins in the Respiratory Tract of Horses
Source: J Histochem Cytochem. 2026 Aug 31:00221554261474565. Online ahead of print. doi: 10.1369/00221554261474565 (PMC13530036; doi:10.1369/00221554261474565)
Supplement: Supplementary file 7 [file sj-docx-7-jhc-10.1369_00221554261474565.docx]

VNN tissue expression in human, mice and horses

| **Tissue** | **Human VNN1** | **Human VNN2** | **Human VNN3** | **Murine VNN1** | **Murine VNN3** | **Equine VNN1** | **Equine VNN2** | **Equine VNN3** |
| --- | --- | --- | --- | --- | --- | --- | --- | --- |
| Brain | n.a. | n.a. | X^2^ | n.a. | n.a. | *See below* | *See below* | *See below* |
| Cerebrum | n.a. | n.a. | n.a. | n.a. | n.a. | X | qPCR | qPCR |
| Cerebellum | n.a. | n.a. | n.a. | n.a. | n.a. | X | qPCR | qPCR |
| Pituitary gland | n.a. | n.a. | n.a. | n.a. | n.a. | X | qPCR | qPCR |
| Conjunctiva | n.a. | n.a. | n.a. | n.a. | n.a. | qPCR | qPCR | qPCR |
| Nasal mucosa | n.a. | n.a. | n.a. | n.a. | n.a. | qPCR | qPCR | qPCR |
| Tongue | qPCR^4^ | qPCR^4^ | qPCR^4^ | n.a. | n.a. | n.a. | n.a. | n.a. |
| Pharynx | qPCR^4^ | qPCR^4^ | qPCR^4^ | n.a. | n.a. | n.a. | n.a. | n.a. |
| Epiglottis | X^4^ | X^4^ | qPCR^4^ | n.a. | n.a. | n.a. | n.a. | n.a. |
| Gingiva | X^4^ | qPCR^4^ | qPCR^4^ | n.a. | n.a. | n.a. | n.a. | n.a. |
| Vocal folds | X^4^ | X^4^ | qPCR^4^ | n.a. | n.a. | n.a. | n.a. | n.a. |
| Thyroid glands | n.a. | n.a. | n.a. | n.a. | n.a. | qPCR | qPCR | qPCR |
| Trachea | X^4^ | qPCR^4^ | qPCR^4^ | n.a. | n.a. | *See below* | *See below* | *See below* |
| Trachea: cranial | n.a. | n.a. | n.a. | n.a. | n.a. | qPCR | qPCR | qPCR |
| Trachea: middle | n.a. | n.a. | n.a. | n.a. | n.a. | qPCR | qPCR | qPCR |
| Trachea: caudal | n.a. | n.a. | n.a. | n.a. | n.a. | qPCR | qPCR | qPCR |
| Bronchi | qPCR^4^ | qPCR^4^ | qPCR^4^ | n.a. | n.a. | *See below* | *See below* | *See below* |
| Large bronchi | n.a. | n.a. | n.a. | n.a. | n.a. | qPCR | qPCR | qPCR |
| Middle bronchi | n.a. | n.a. | n.a. | n.a. | n.a. | qPCR | qPCR | qPCR |
| Small bronchi | n.a. | n.a. | n.a. | n.a. | n.a. | qPCR | qPCR | qPCR |
| Lung | qPCR^4^ | qPCR^4^ | X^2^, qPCR^4^ | qPCR^5^ | n.a. | *See below* | *See below* | *See below* |
| Cranial lung | n.a. | n.a. | n.a. | n.a. | n.a. | qPCR | qPCR | qPCR |
| Caudal lung | n.a. | n.a. | n.a. | n.a. | n.a. | qPCR | qPCR | qPCR |
| Heart | qPCR^4^ | qPCR^4^ | X^2,4^ | NB^3^ | qPCR^2^ | *See below* | *See below* | *See below* |
| Right atrium | n.a. | n.a. | n.a. | n.a. | n.a. | qPCR | X | qPCR |
| Right ventricle | n.a. | n.a. | n.a. | n.a. | n.a. | X | X | qPCR |
| Aorta | X^4^ | qPCR^4^ | n.a. | n.a. | n.a. | X | X | qPCR |
| Thymus | NB^1^ | NB^1^ | X^2^ | n.a. | qPCR^2^ | n.a. | n.a. | n.a. |
| Esophagus | qPCR^4^ | qPCR^4^ | qPCR^4^ | n.a. | n.a. | qPCR | X | qPCR |
| Stomach | qPCR^4^ | qPCR^4^ | qPCR^4^ | n.a. | n.a. | X | X | X |
| Intestine (not specified) | n.a. | n.a. | n.a. | NB^3^ | qPCR^2^ | *See below* | *See below* | *See below* |
| Small Intestine (not specified) | NB^1^ | *See below* | X^2^ | n.a. | n.a. | *See below* | *See below* | *See below* |
| Duodenum | n.a. | n.a. | n.a. | n.a. | n.a. | qPCR | qPCR | qPCR |
| Jejunum | n.a. | n.a. | n.a. | n.a. | n.a. | qPCR | qPCR | qPCR |
| Ileum | qPCR^4^ | qPCR^4^ | qPCR^4^ | n.a. | n.a. | qPCR | qPCR | qPCR |
| Caecum | n.a. | n.a. | n.a. | n.a. | n.a. | qPCR | qPCR | qPCR |
| Colon | X^4^ | X^4^ | X^2,4^ | n.a. | n.a. | qPCR | qPCR | qPCR |
| Rectum | n.a. | n.a. | n.a. | n.a. | n.a. | X | qPCR | X |
| Liver | qPCR^4^ | qPCR^4^ | NB^2^, qPCR^4^ | NB^3^ | NB^2^, qPCR^2^ | qPCR | qPCR | qPCR |
| Pancreas | X^4^ | qPCR^4^ | X^4^ | n.a. | n.a. | qPCR | X | qPCR |
| spleen | NB^1^, qPCR^4^ | NB^1^, qPCR^4^ | X^2,4^ | n.a. | qPCR^2^ | X | qPCR | qPCR |
| Ureter | qPCR^4^ | qPCR^4^ | qPCR^4^ | n.a. | n.a. | n.a. | n.a. | n.a. |
| Kidneys | qPCR^4^ | NB^1^, qPCR^4^ | X^2^ | NB^3^ | qPCR^2^ | qPCR | qPCR | qPCR |
| Adrenal glands | n.a. | n.a. | n.a. | n.a. | n.a. | X | qPCR | qPCR |
| Urinary bladder | X^4^ | qPCR^4^ | X^4^ | n.a. | n.a. | X | qPCR | qPCR |
| Ureter | qPCR^4^ | qPCR^4^ | qPCR^4^ | n.a. | n.a. | n.a. | n.a. | n.a. |
| Mammary glands | qPCR^4^ | X^4^ | X^4^ | n.a. | n.a. | n.a. | n.a. | n.a. |
| Testis | qPCR^4^ | qPCR^4^ | X^4^ | NB^3^ | qPCR^2^ | n.a. | n.a. | n.a. |
| Skin | qPCR^4^ | X^4^ | qPCR^4^ | n.a. | n.a. | X | qPCR | qPCR |
| Lymphnodes | qPCR^4^ | qPCR^4^ | qPCR^4^ | n.a. | n.a. | n.a. | n.a. | n.a. |
| Muscle | X^4^ | qPCR^4^ | X^2, 4^ | n.a. | n.a. | X | qPCR | qPCR |
| Blood | qPCR^4^ | qPCR^4^ | qPCR^4^ | n.a. | n.a. | n.a. | n.a. | n.a. |

*Method of detection is listed in the table: quantitative real time PCR (qPCR) or Northern Blot (NB). X = no detection; n.a. = data not available (^1^Galland et al. 1998; ^2^Martin et al. 2001; ^3^Pitari et al. 2000; ^4^Jansen et al. 2009; ^5^Xing et al. 2020)*
