# Supplementary material for: Ortholog-specific Spatial Expression of Vanins in the Respiratory Tract of Horses
Source: J Histochem Cytochem. 2026 Aug 31:00221554261474565. Online ahead of print. doi: 10.1369/00221554261474565 (PMC13530036; doi:10.1369/00221554261474565)
Supplement: Supplementary file 8 [file sj-docx-5-jhc-10.1369_00221554261474565.docx]

## eVNN2 expression in blood monocytes
